# Supplementary material for: Keratinocytes drive the epithelial hyperplasia key to sea lice resistance in coho salmon
Source: BMC Biol. 2024 Jul 29;22:160. doi: 10.1186/s12915-024-01952-8 (PMC11287951; doi:10.1186/s12915-024-01952-8)
Supplement: Supplementary file 1 — Additional file 1: Figs. S1–S55 and Tables S1–S9. Figs. S1–S55: Fig. S1 Expression of marker genes within 23 identified cell clusters within Atlantic salmon fin and skin samples. Fig. S2 Expression of marker genes within 23 identified cell clusters within coho salmon fin and skin samples. Fig. S3 Violin plots of the expression of marker genes from Fig. 1c for each cell type detected within Atlantic salmon samples split by tissue type. Fig. S4 Violin plots of the expression of marker genes from Fig. 1d for each cell type detected within coho salmon samples split by tissue type. Fig. S5 Violin plots of expression levels for the top 20 significant marker genes for the mucous (1) cluster of the coho salmon dataset. Fig. S6 Violin plots of expression levels for the top 20 significant marker genes for the mucous (2) cluster of the coho salmon dataset. Fig. S7 Violin plots of expression levels for the top 20 significant marker genes for the undifferentiated cluster of the Atlantic salmon dataset. Fig. S8 Expression of CD45 (ptprc) in Atlantic salmon (a, b) and coho salmon (c, d). Fig. S9 Violin plots of expression levels for the top 20 significant marker genes for the T cells (1) cluster of the Atlantic salmon immune cells only data subset. Fig. S10 Violin plots of expression levels for the top 20 significant marker genes for the T cells (2) cluster of the Atlantic salmon immune cells only data subset. Fig. S11 Violin plots of expression levels for the top 20 significant marker genes for the T cells (3) cluster of the Atlantic salmon immune cells only data subset. Fig. S12 Violin plots of expression levels for the top 20 significant marker genes for the T cells (4) cluster of the Atlantic salmon immune cells only data subset. Fig. S13 Violin plots of expression levels for the top 20 significant marker genes for the T cells (5) cluster of the Atlantic salmon immune cells only data subset. Fig. S14 Violin plots of expression levels for the top 20 significant marker genes for [file 12915_2024_1952_MOESM1_ESM.pdf]

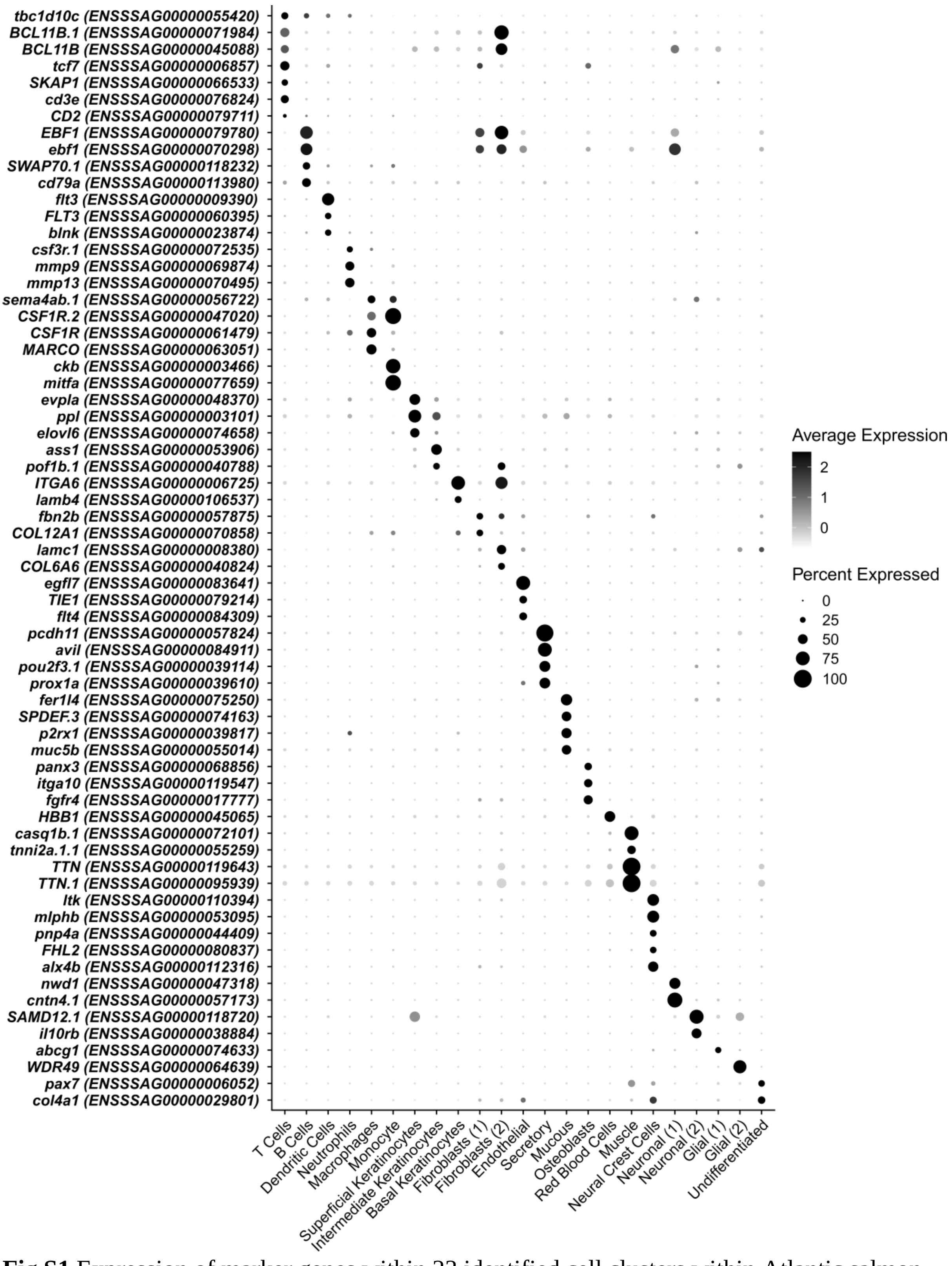

Fig.S1 Expression of marker genes within 23 identified cell clusters within Atlantic salmon fin and skin samples.

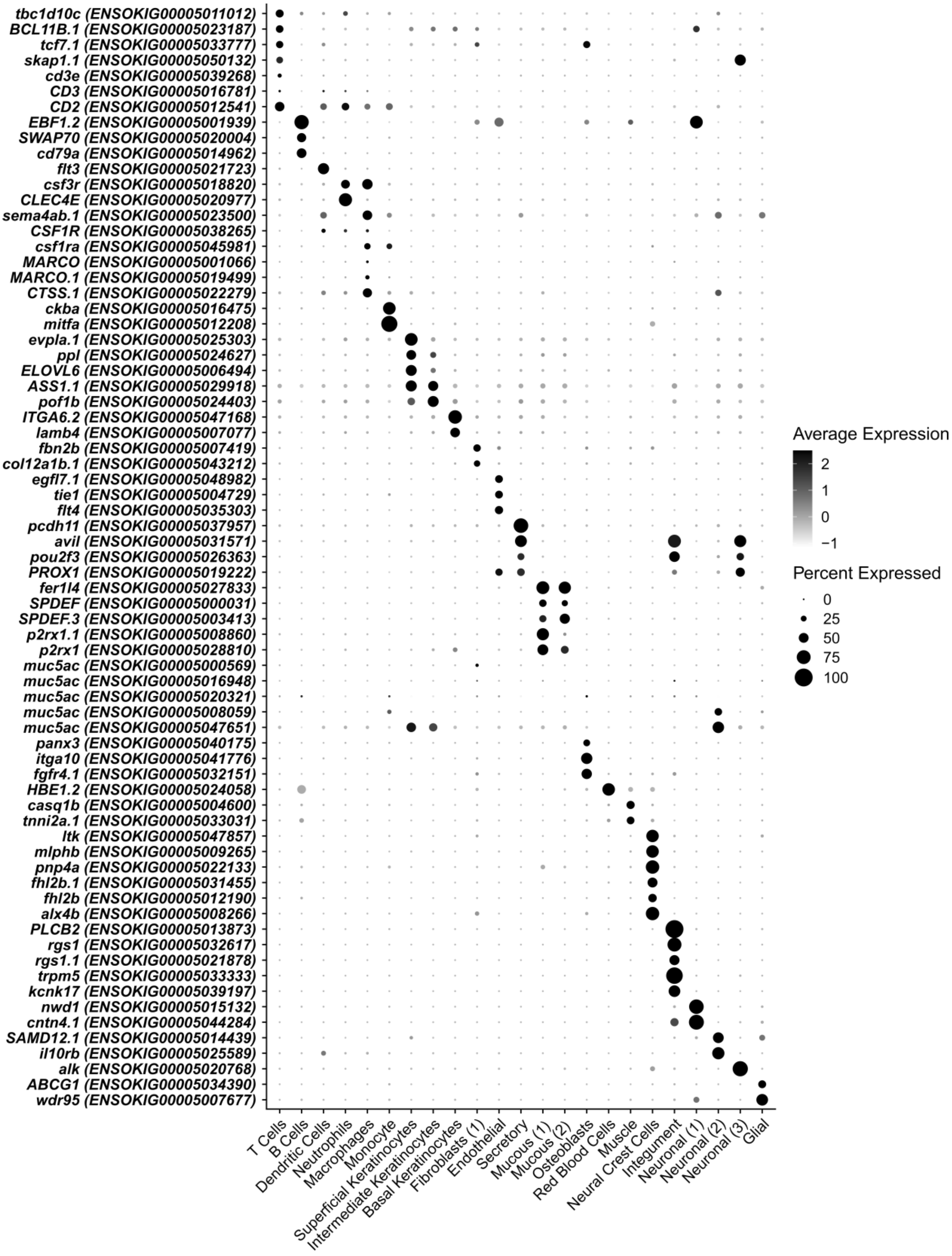

Fig.S2 Expression of marker genes within 23 identified cell clusters within coho salmon fin and skin samples.

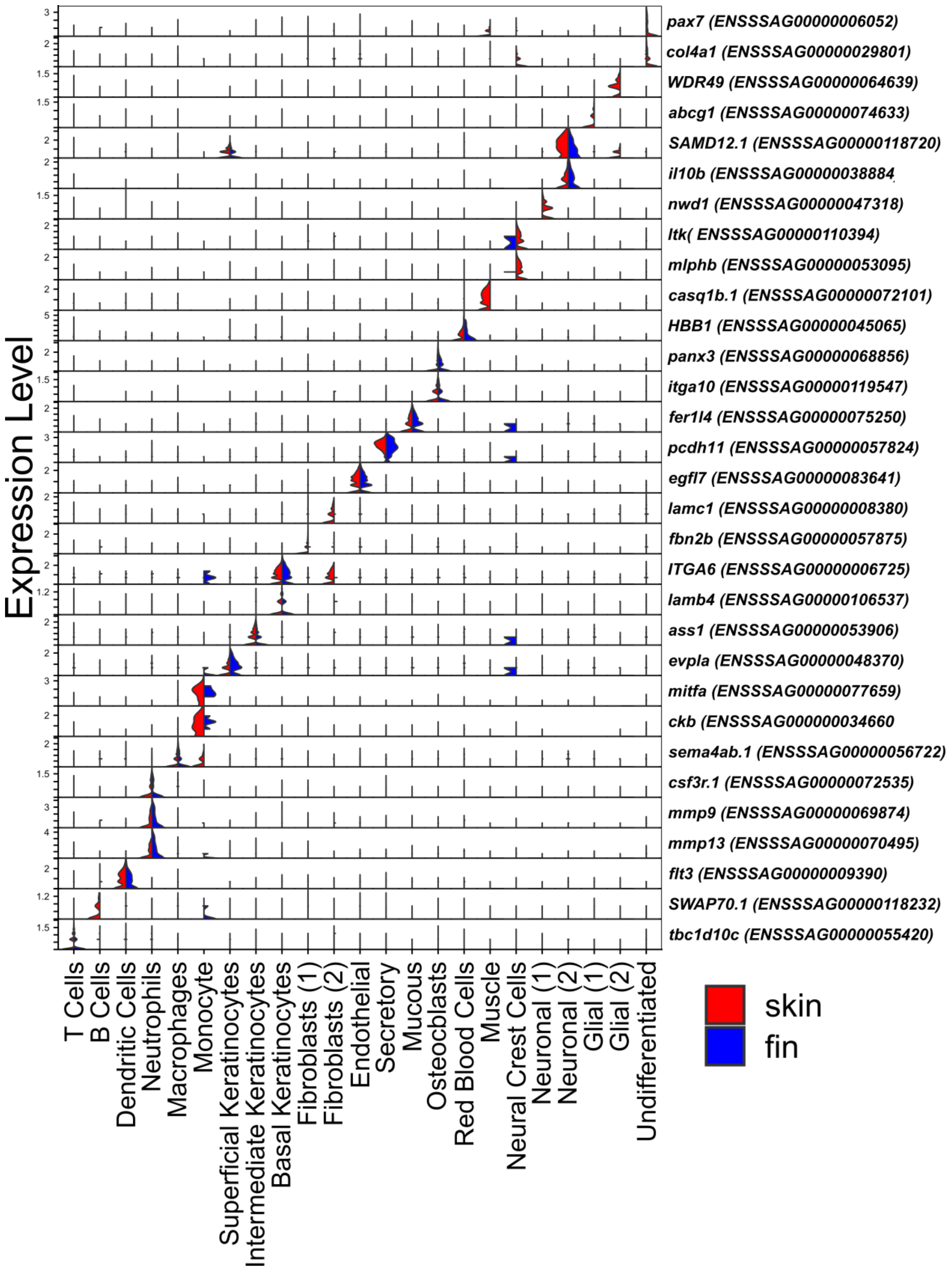

Fig.S3 Violin plots of the expression of marker genes from Fig.1c for each cell type detected within Atlantic salmon samples split by tissue type (skin or pelvic fin). Note that the cell types: red blood cells, muscle, neural crest cells, neuronal (1), and glial cell clusters were almost exclusively observed in skin samples.

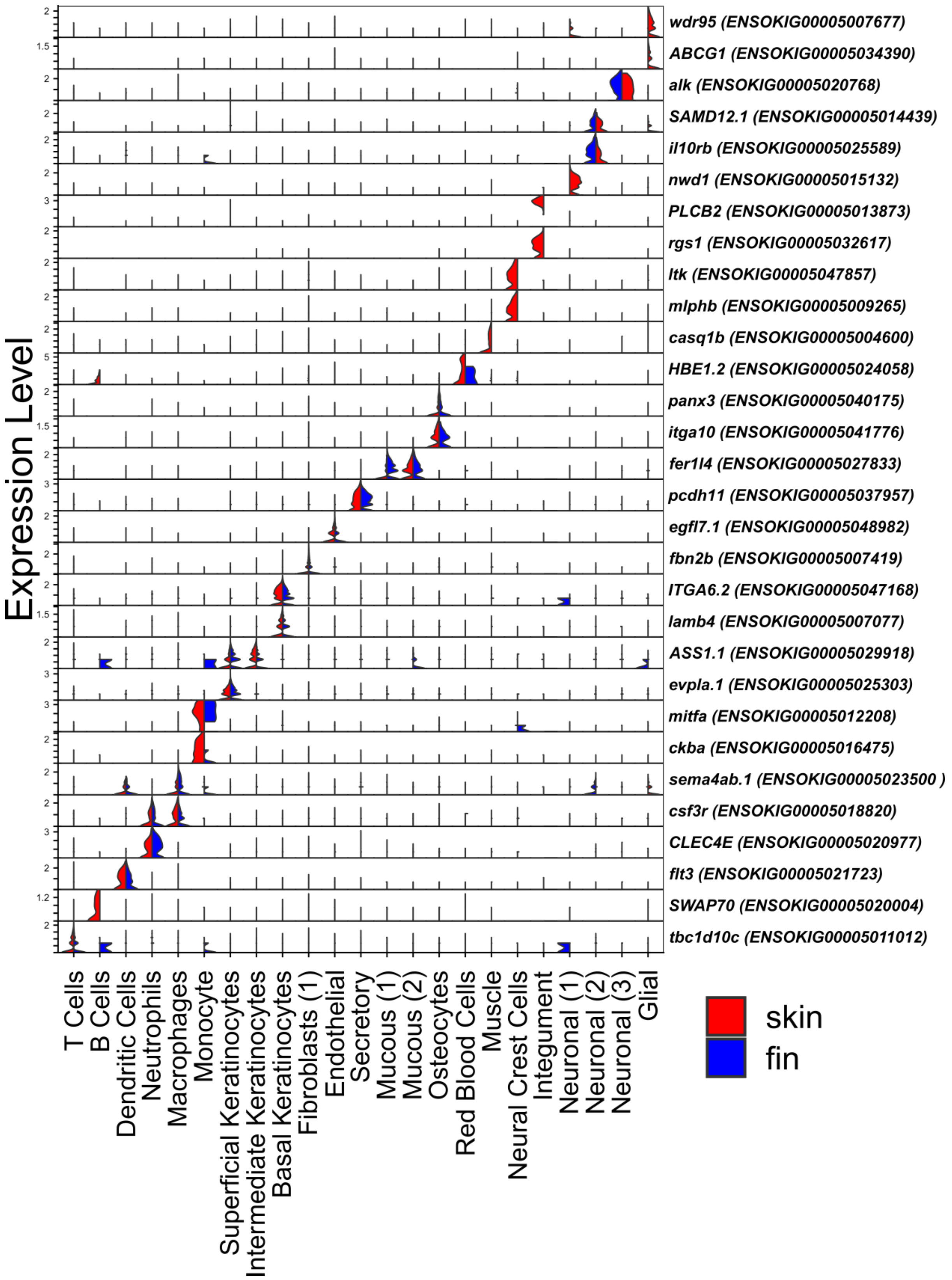

Fig.S4 Violin plots of the expression of marker genes from Fig.1d for each cell type detected within coho salmon samples split by tissue type (skin or pelvic fin). Note that the cell types: red blood cells, muscle, neural crest cells, neuronal (1), and glial cell clusters were almost exclusively observed in skin samples.

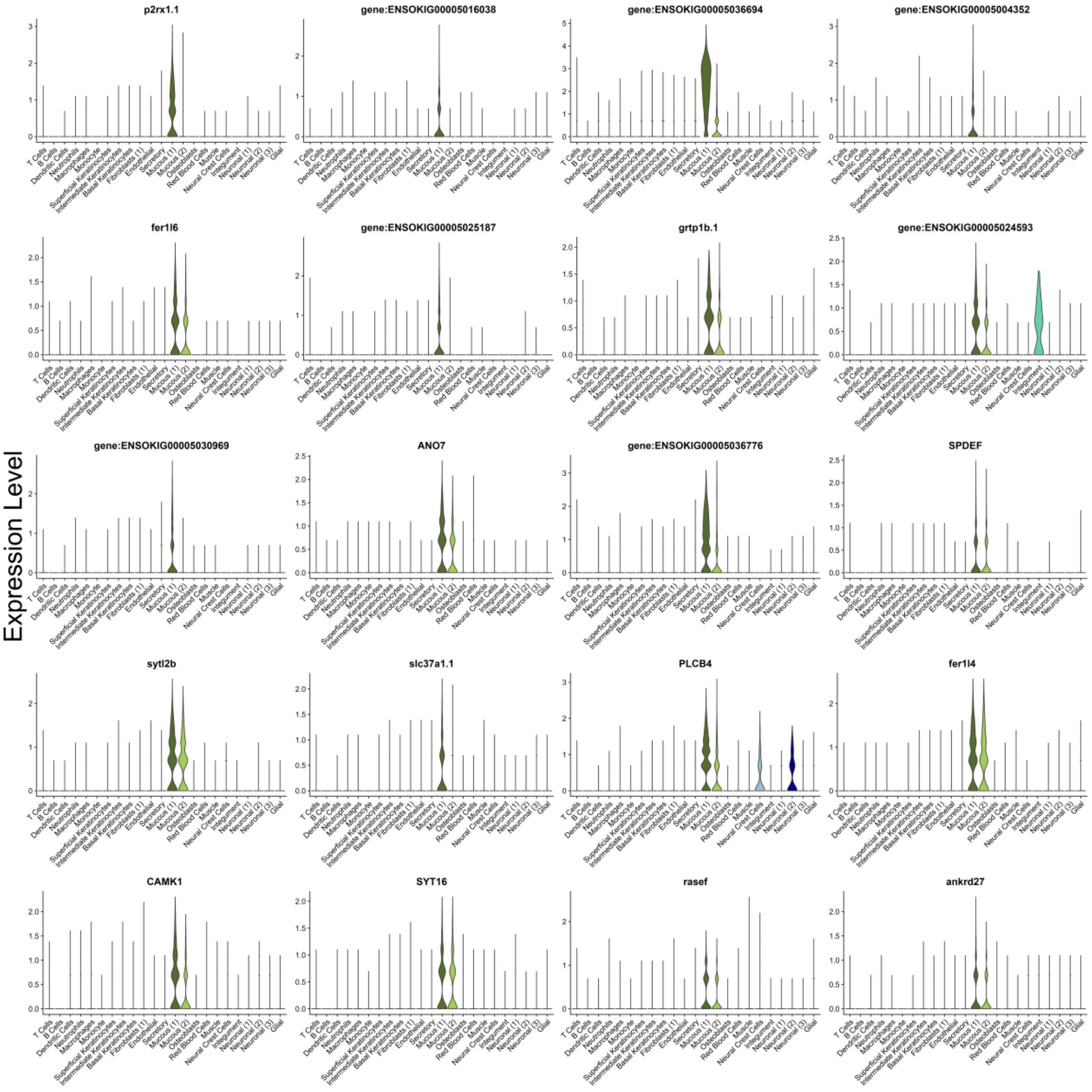

Fig.S5 Violin plots of expression levels (based on the SCT assay) for the top 20 significant (adjusted p-value < 0.05) marker genes for the mucous (1) cluster (based on log-scale two-fold change in expression) of the coho salmon dataset (for UMAP see Fig.1).

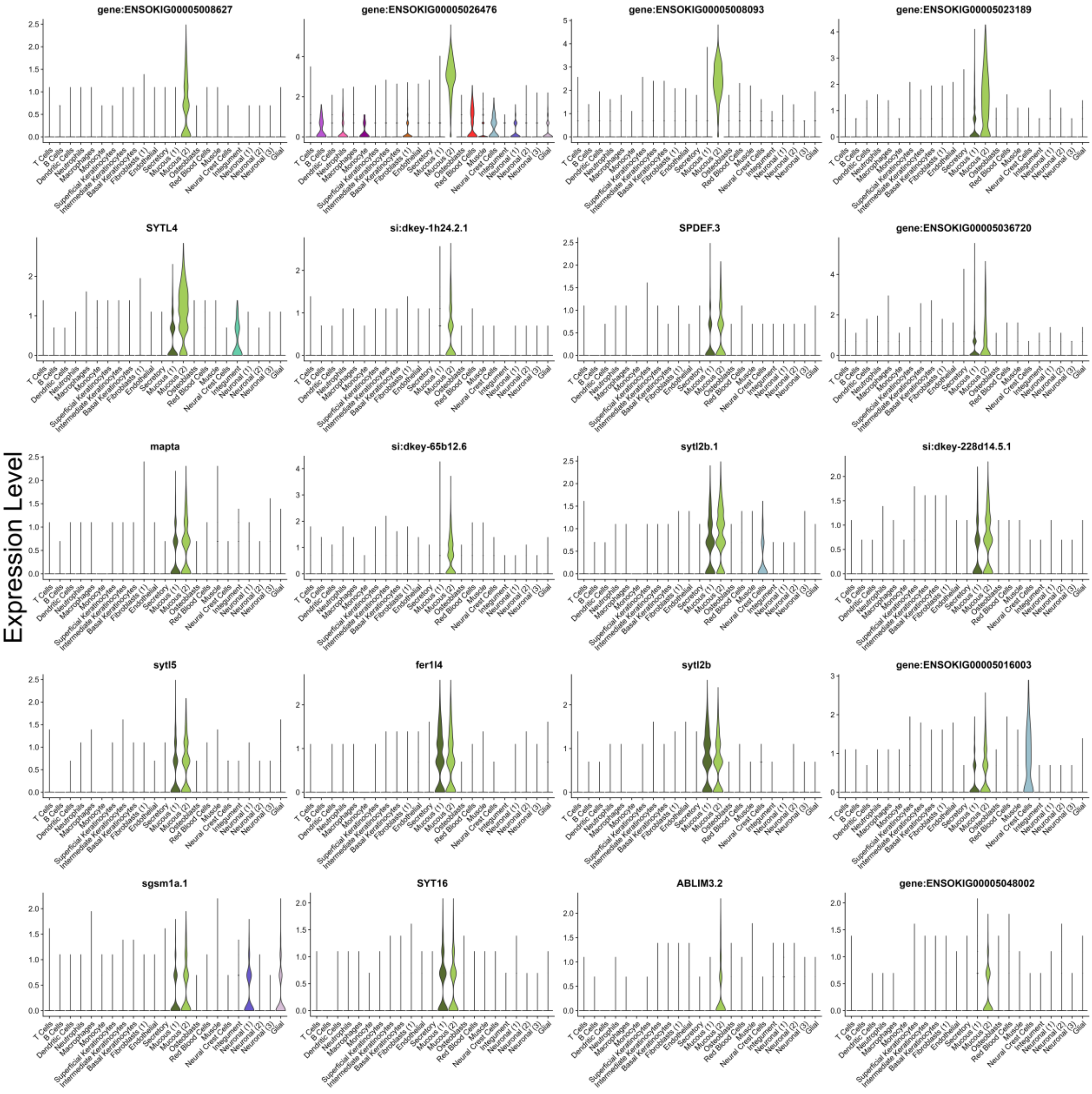

Fig.S6 Violin plots of expression levels (based on the SCT assay) for the top 20 significant (adjusted p-value < 0.05) marker genes for the mucous (2) cluster (based on log-scale two-fold change in expression) of the coho salmon dataset (for UMAP see Fig.1).

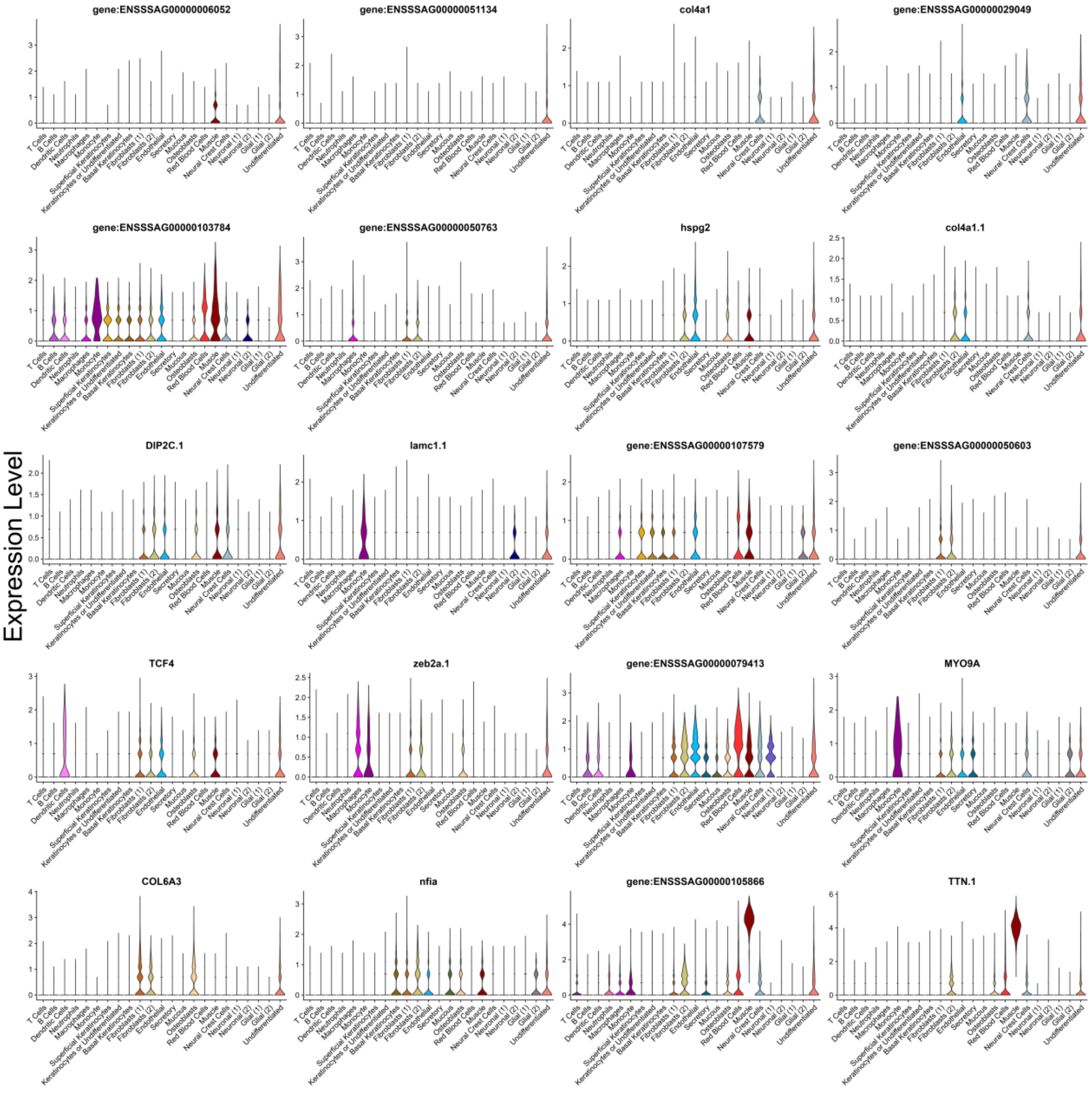

Fig.S7 Violin plots of expression levels (based on the SCT assay) for the top 20 significant (adjusted p-value < 0.05) marker genes for the undifferentiated cluster (based on log-scale two-fold change in expression) of the Atlantic salmon dataset (for UMAP see Fig.1).

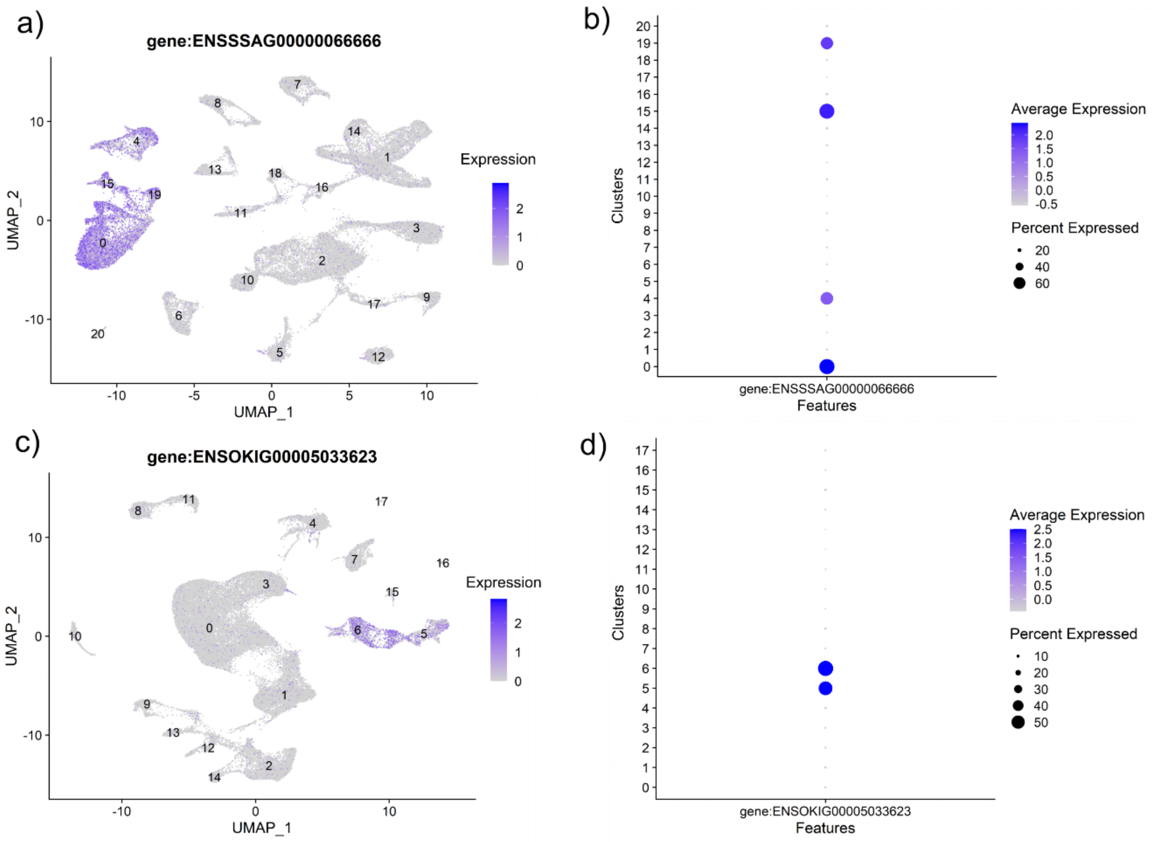

Fig.S8 Expression of *CD45* (*ptprc*) in Atlantic salmon (a, b) and coho salmon (c, d). Feature Plots (a, c) indicate expression (based on the SCT assay) of *CD45*. Dotplots (b, d) indicate average expression (based on the SCT assay) of *CD45* for each cluster.

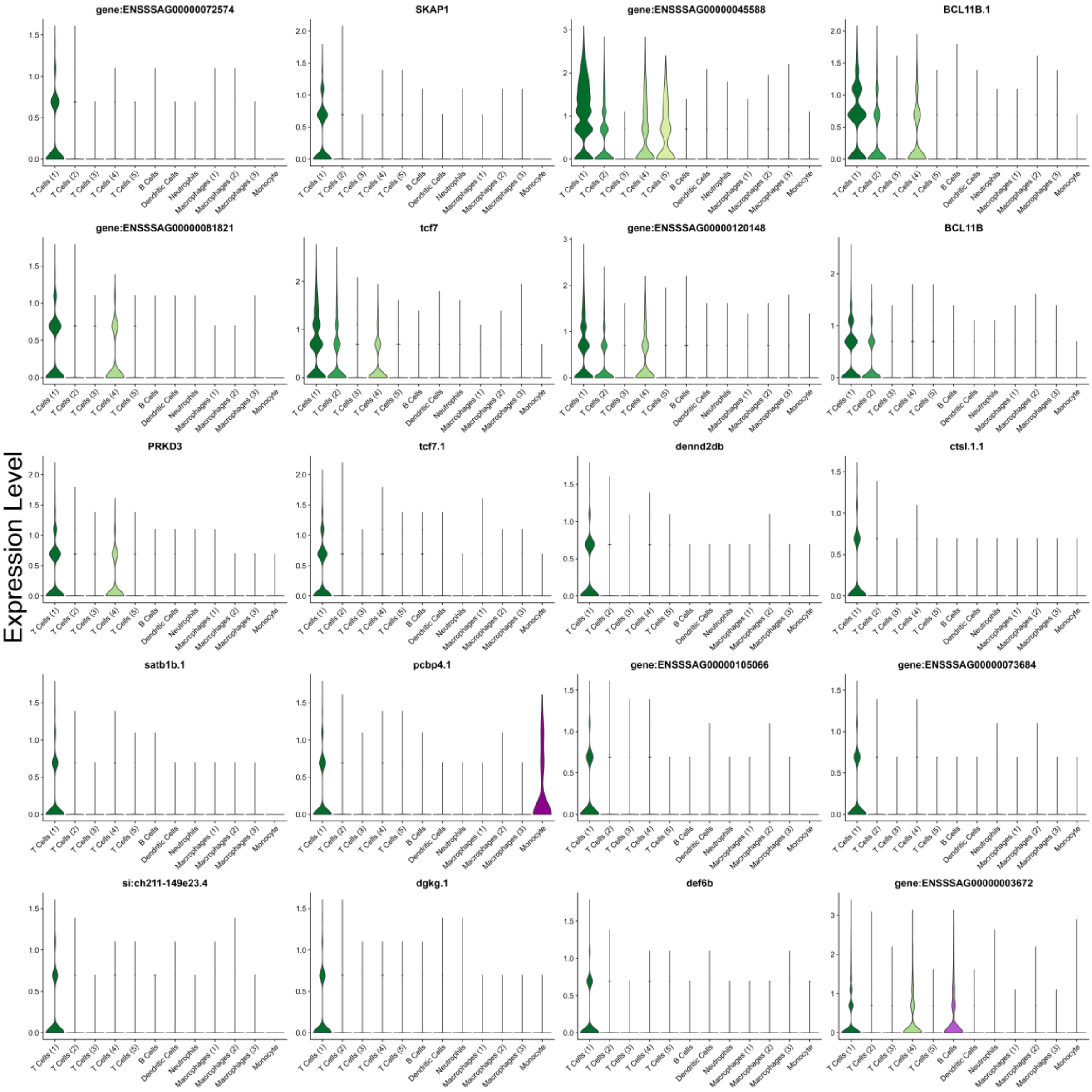

Fig.S9 Violin plots of expression levels (based on the SCT assay) for the top 20 significant (adjusted p-value < 0.05) marker genes for the T cells (1) cluster (based on log-scale two-fold change in expression) of the Atlantic salmon immune cells only data subset (for UMAP see Fig.3a).

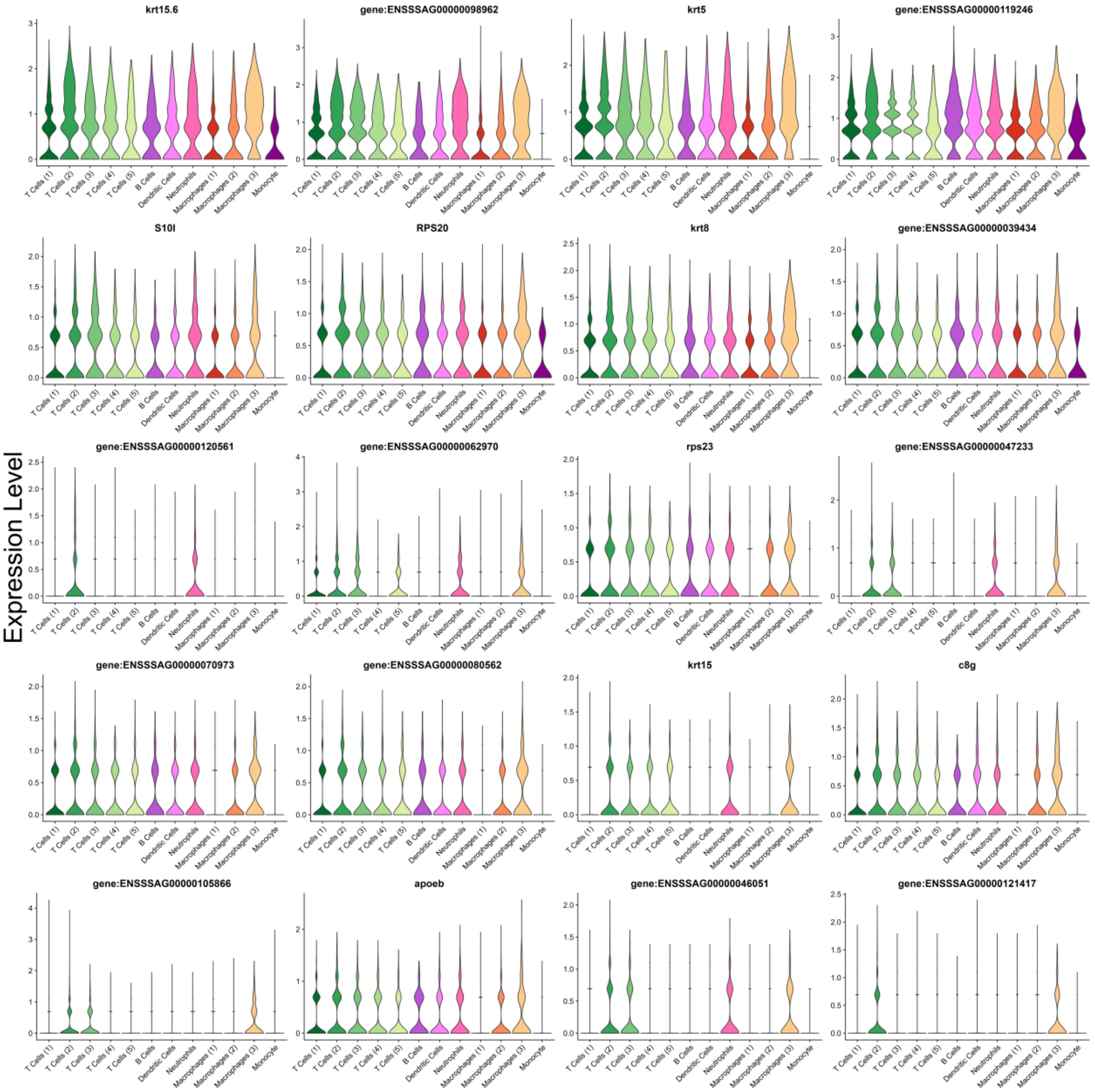

Fig.S10 Violin plots of expression levels (based on the SCT assay) for the top 20 significant (adjusted p-value < 0.05) marker genes for the T cells (2) cluster (based on log-scale two-fold change in expression) of the Atlantic salmon immune cells only data subset (for UMAP see Fig.3a).

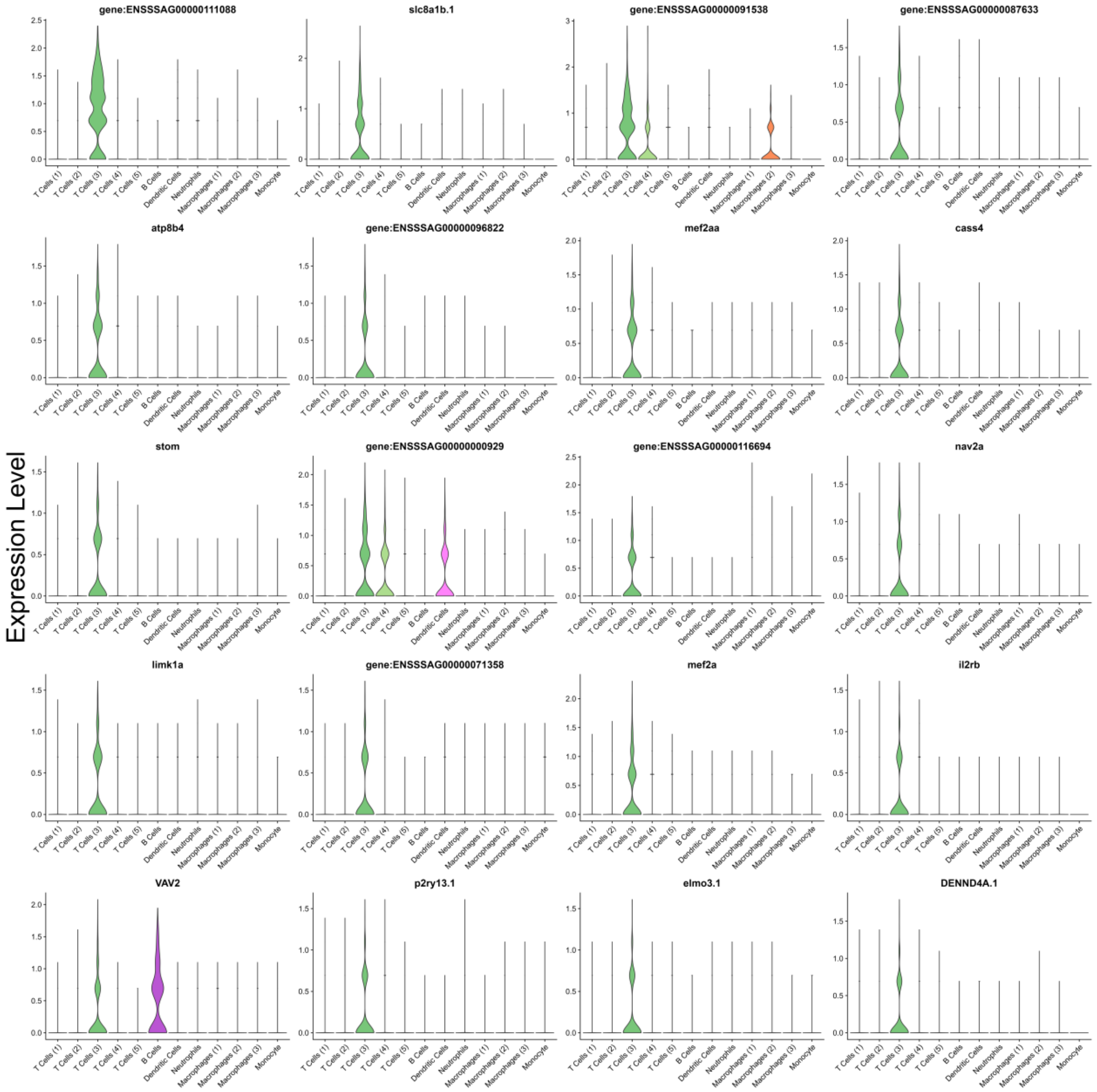

Fig.S11 Violin plots of expression levels (based on the SCT assay) for the top 20 significant (adjusted p-value < 0.05) marker genes for the T cells (3) cluster (based on log-scale two-fold change in expression) of the Atlantic salmon immune cells only data subset (for UMAP see Fig.3a).

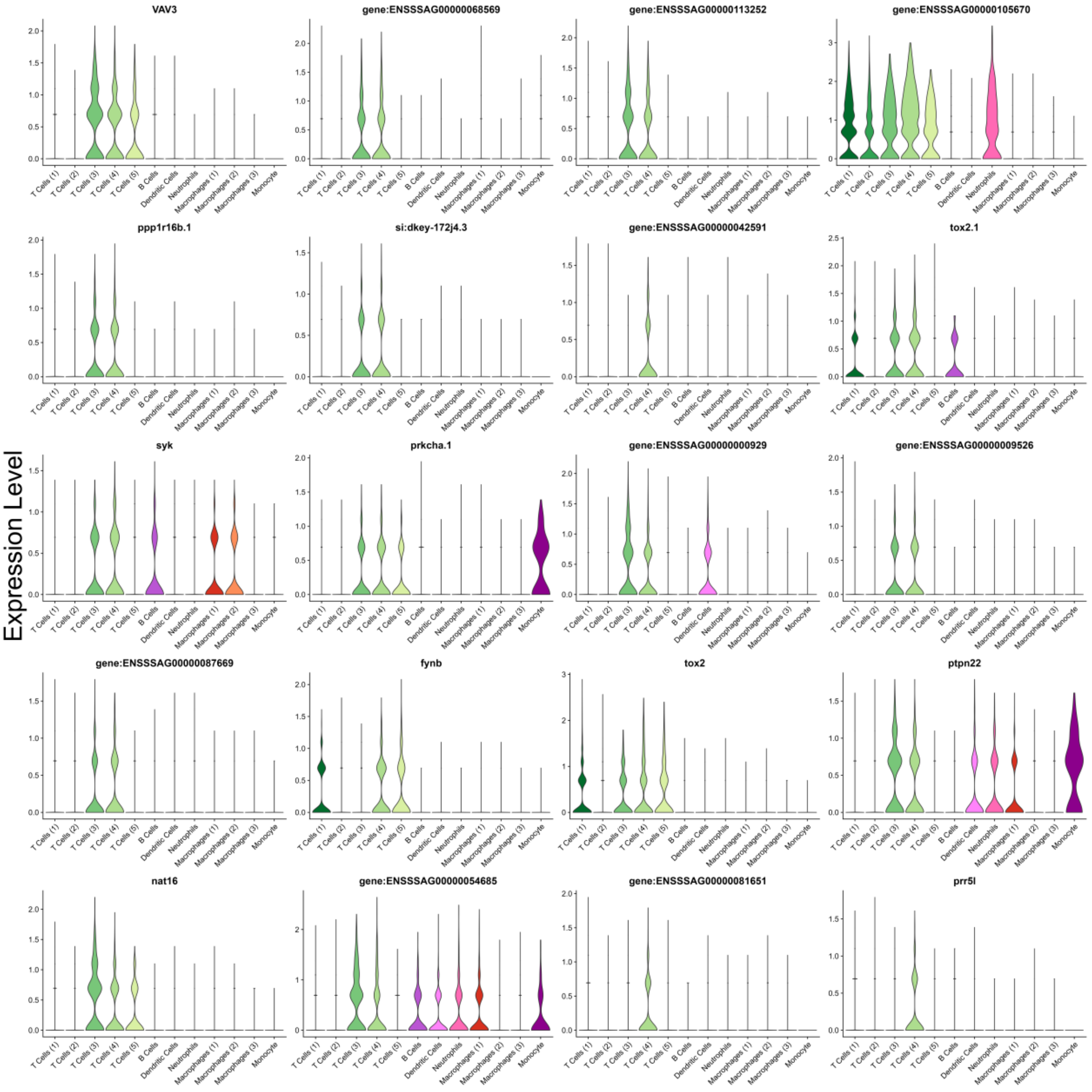

Fig.S12 Violin plots of expression levels (based on the SCT assay) for the top 20 significant (adjusted p-value < 0.05) marker genes for the T cells (4) cluster (based on log-scale two-fold change in expression) of the Atlantic salmon immune cells only data subset (for UMAP see Fig.3a).

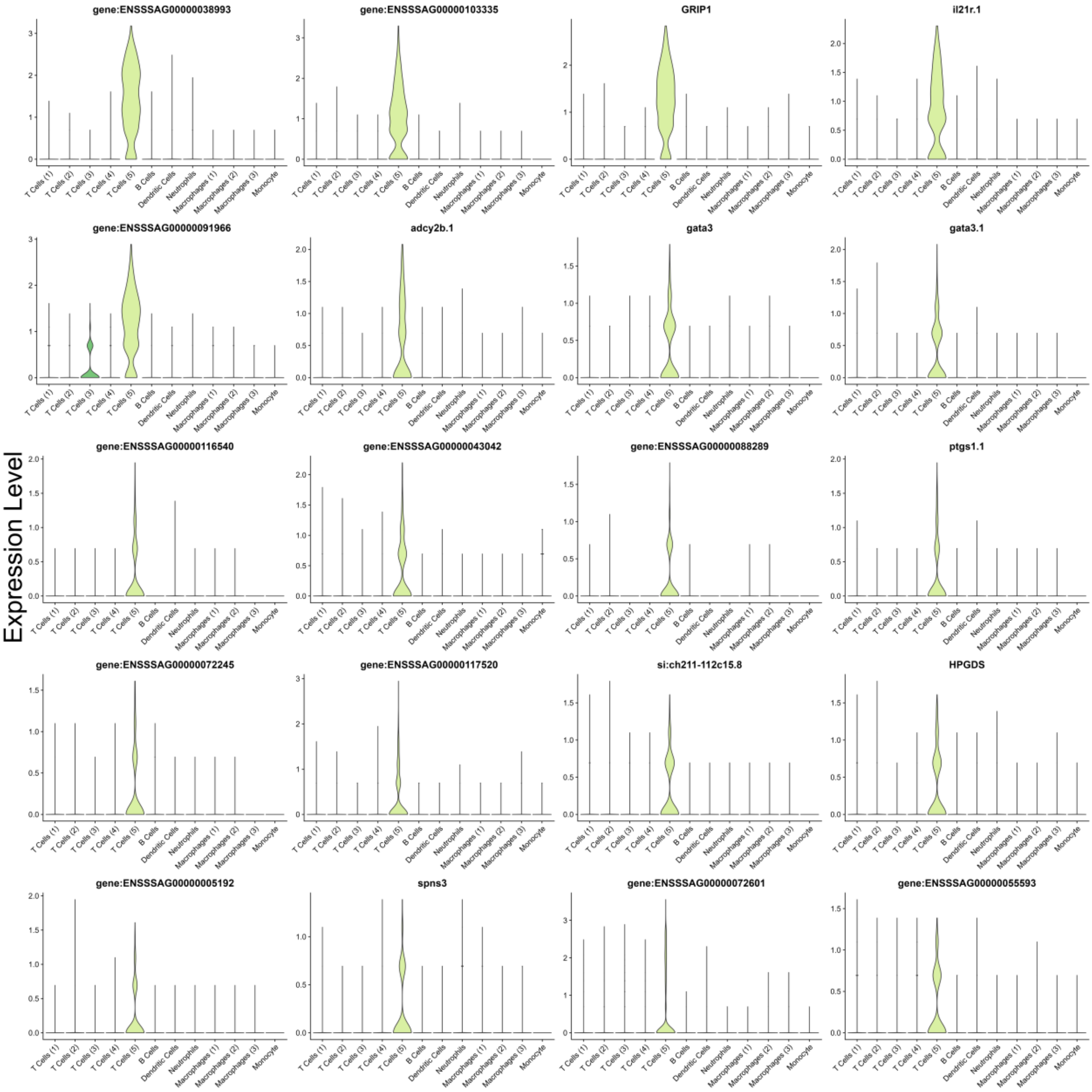

Fig.S13 Violin plots of expression levels (based on the SCT assay) for the top 20 significant (adjusted p-value < 0.05) marker genes for the T cells (5) cluster (based on log-scale two-fold change in expression) of the Atlantic salmon immune cells only data subset (for UMAP see Fig.3a).

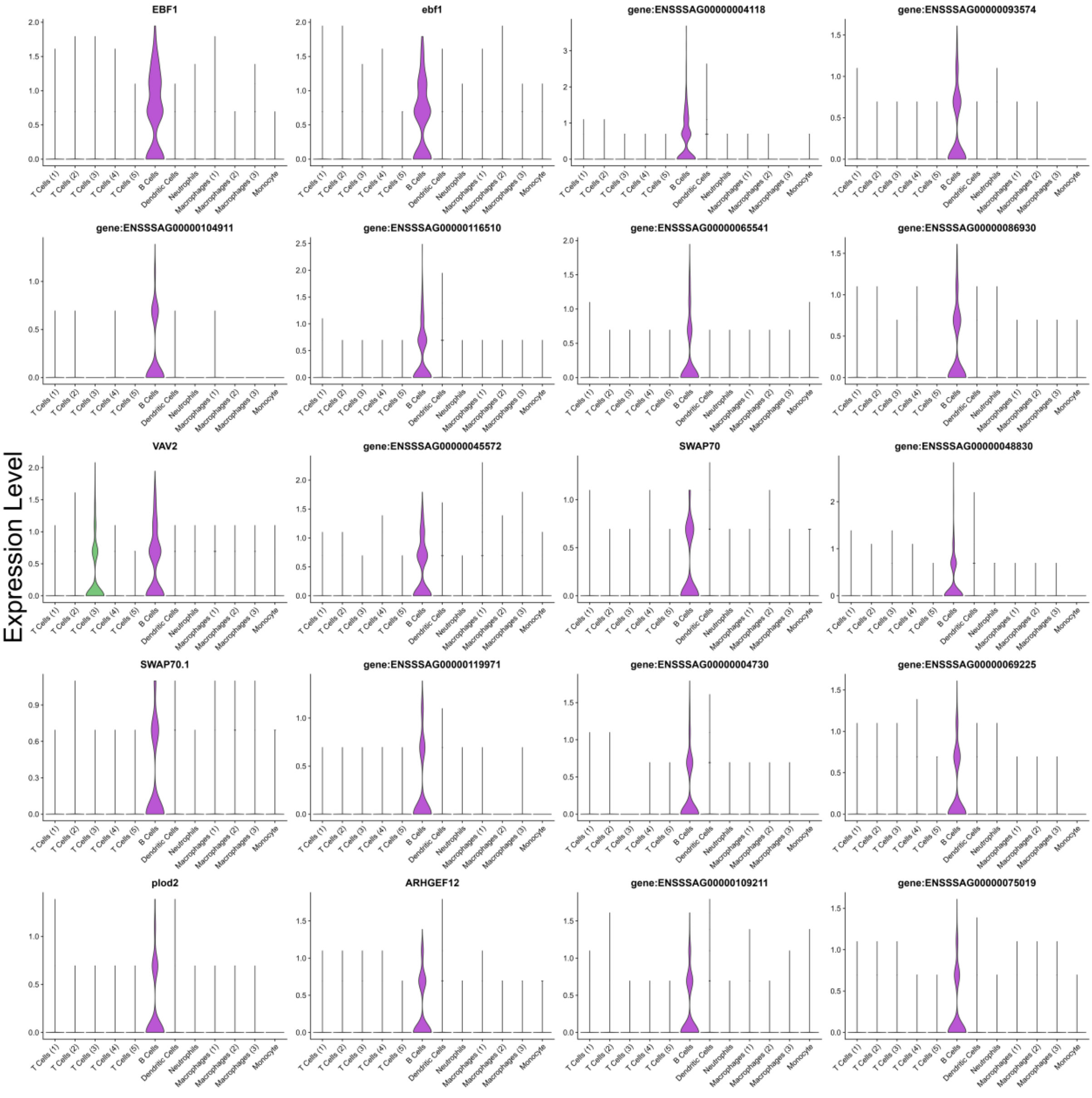

Fig.S14 Violin plots of expression levels (based on the SCT assay) for the top 20 significant (adjusted p-value < 0.05) marker genes for B cells (based on log-scale two-fold change in expression) of the Atlantic salmon immune cells only data subset (for UMAP see Fig.3a).

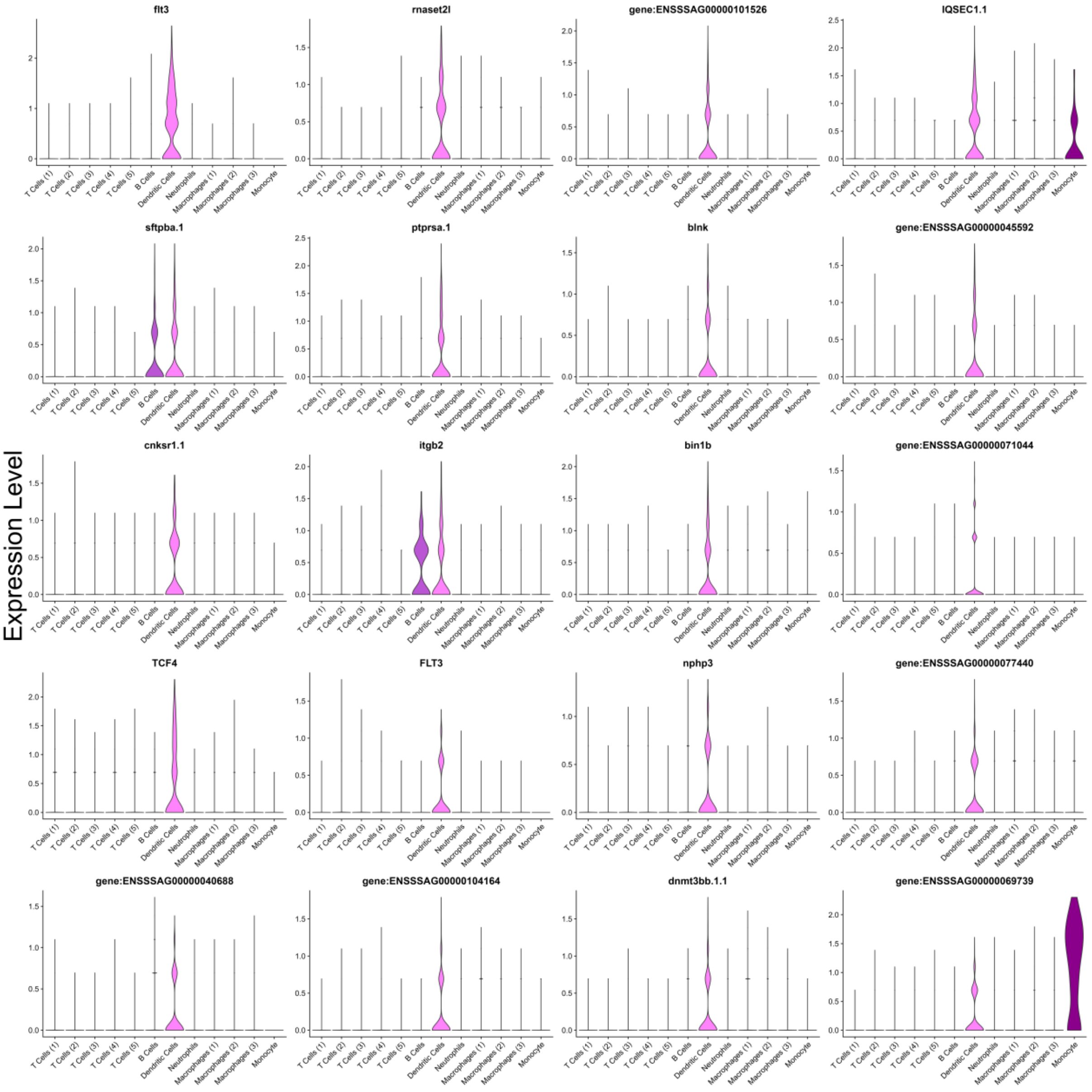

Fig.S15 Violin plots of expression levels (based on the SCT assay) for the top 20 significant (adjusted p-value < 0.05) marker genes for dendritic cells (based on log-scale two-fold change in expression) of the Atlantic salmon immune cells only data subset (for UMAP see Fig.3a).

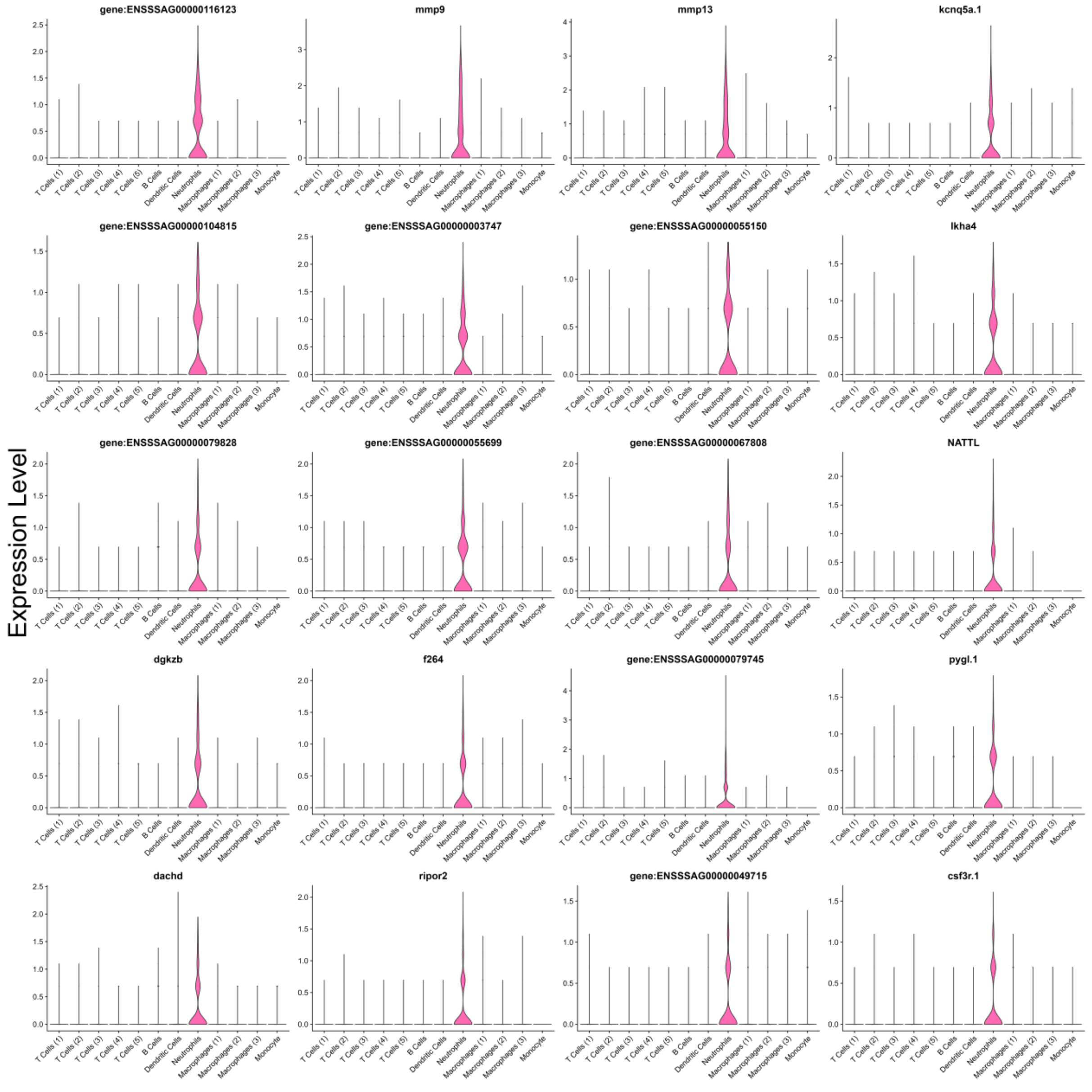

Fig.S16 Violin plots of expression levels (based on the SCT assay) for the top 20 significant (adjusted p-value < 0.05) marker genes for neutrophils (based on log-scale two-fold change in expression) of the Atlantic salmon immune cells only data subset (for UMAP see Fig.3a).

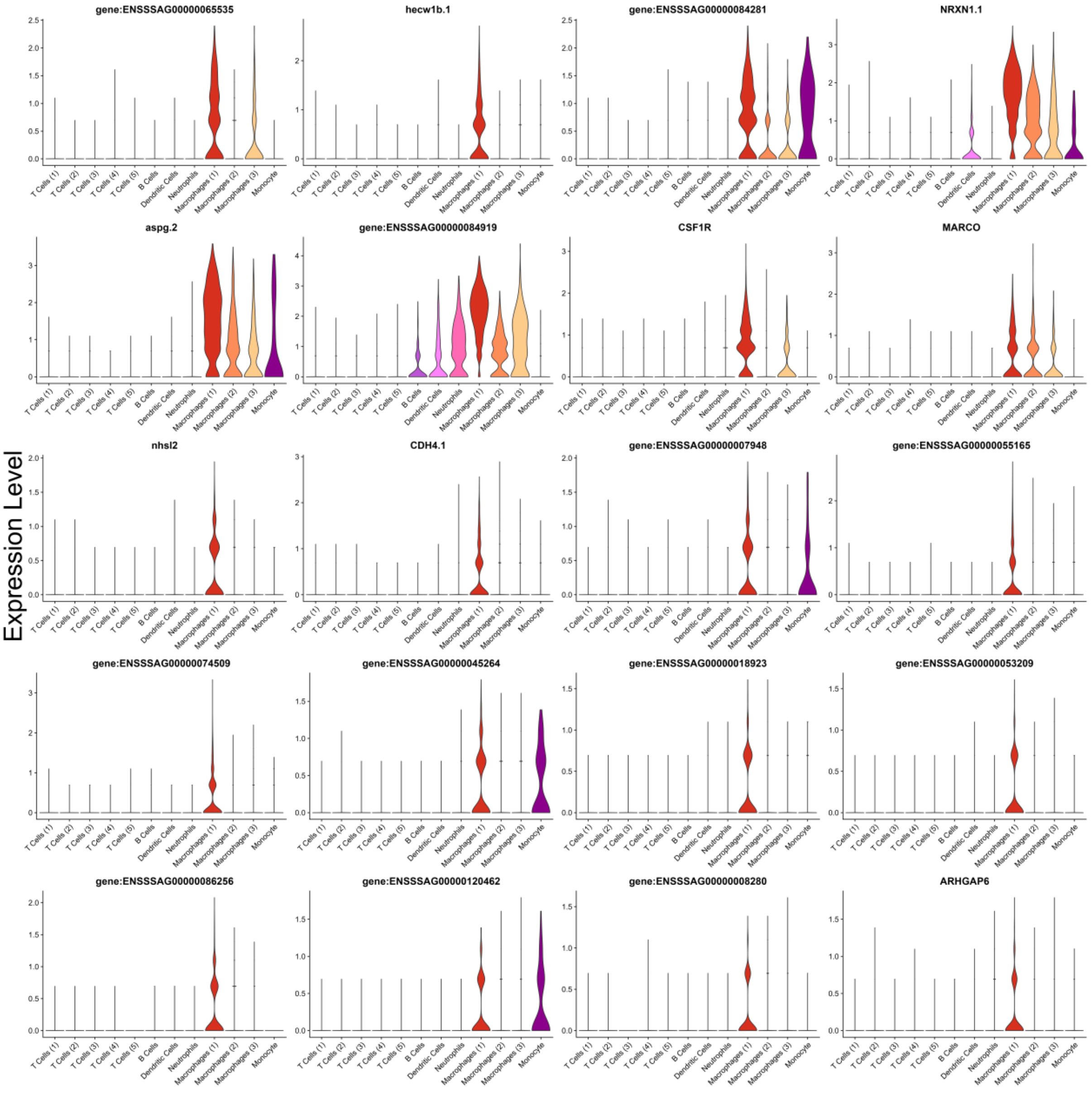

Fig.S17 Violin plots of expression levels (based on the SCT assay) for the top 20 significant (adjusted p-value < 0.05) marker genes for the macrophages (1) cluster (based on log-scale two-fold change in expression) of the Atlantic salmon immune cells only data subset (for UMAP see Fig.3a).

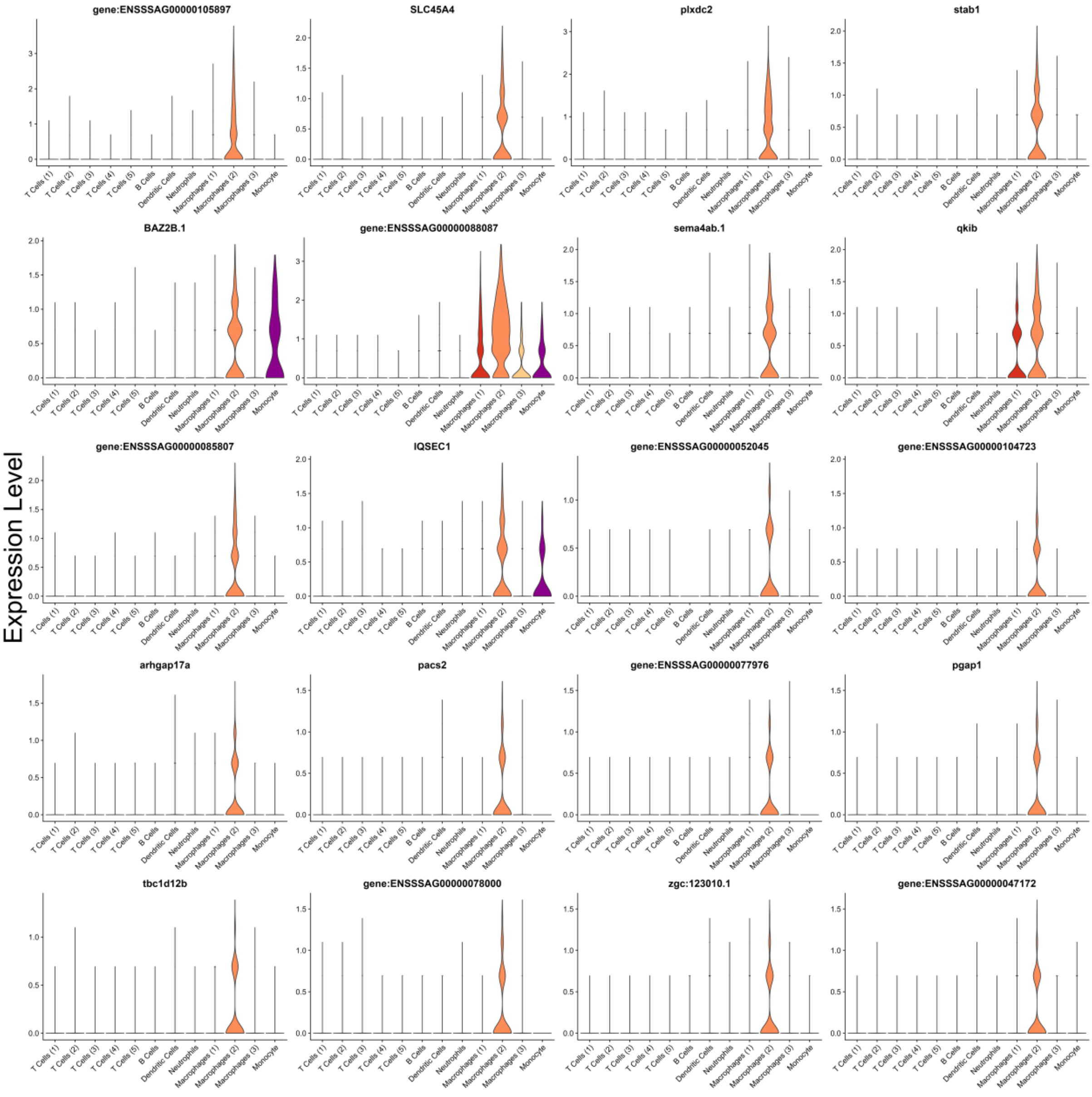

Fig.S18 Violin plots of expression levels (based on the SCT assay) for the top 20 significant (adjusted p-value < 0.05) marker genes for the macrophages (2) cluster (based on log-scale two-fold change in expression) of the Atlantic salmon immune cells only data subset (for UMAP see Fig.3a).

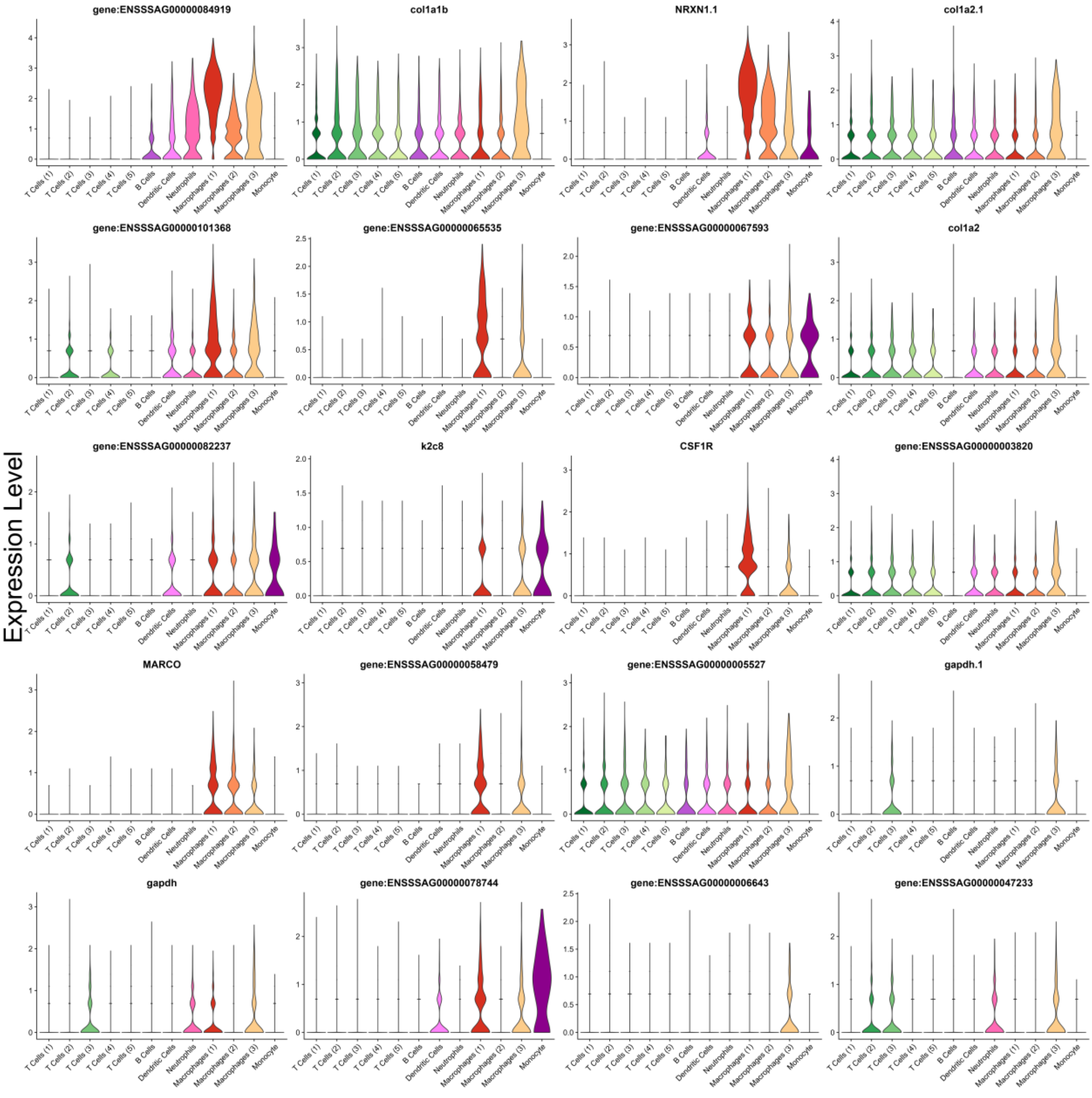

Fig.S19 Violin plots of expression levels (based on the SCT assay) for the top 20 significant (adjusted p-value < 0.05) marker genes for the macrophages (3) cluster (based on log-scale two-fold change in expression) of the Atlantic salmon immune cells only data subset (for UMAP see Fig.3a).

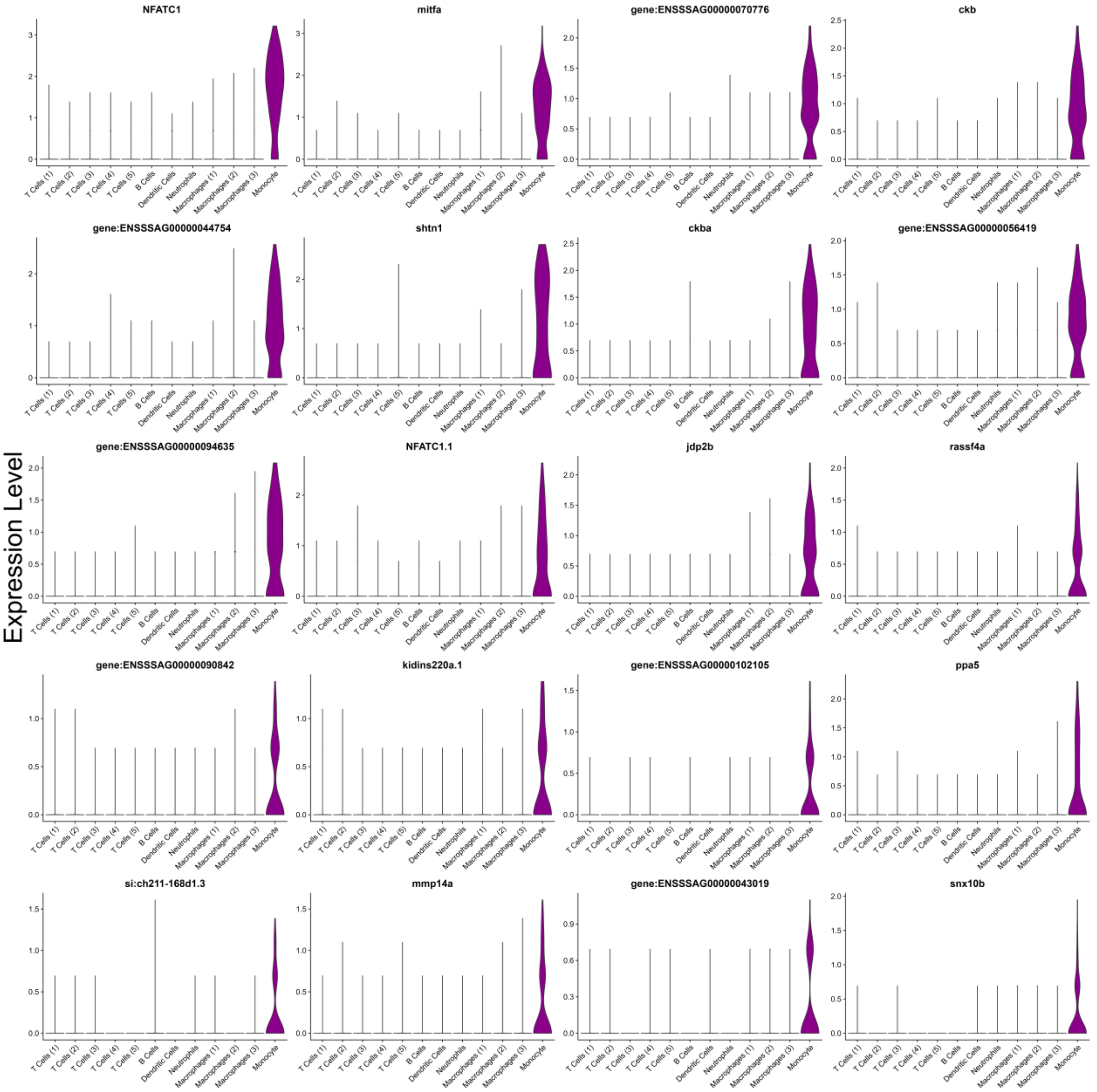

Fig.S20 Violin plots of expression levels (based on the SCT assay) for the top 20 significant (adjusted p-value < 0.05) marker genes for monocytes (based on log-scale two-fold change in expression) of the Atlantic salmon immune cells only data subset (for UMAP see Fig.3a).

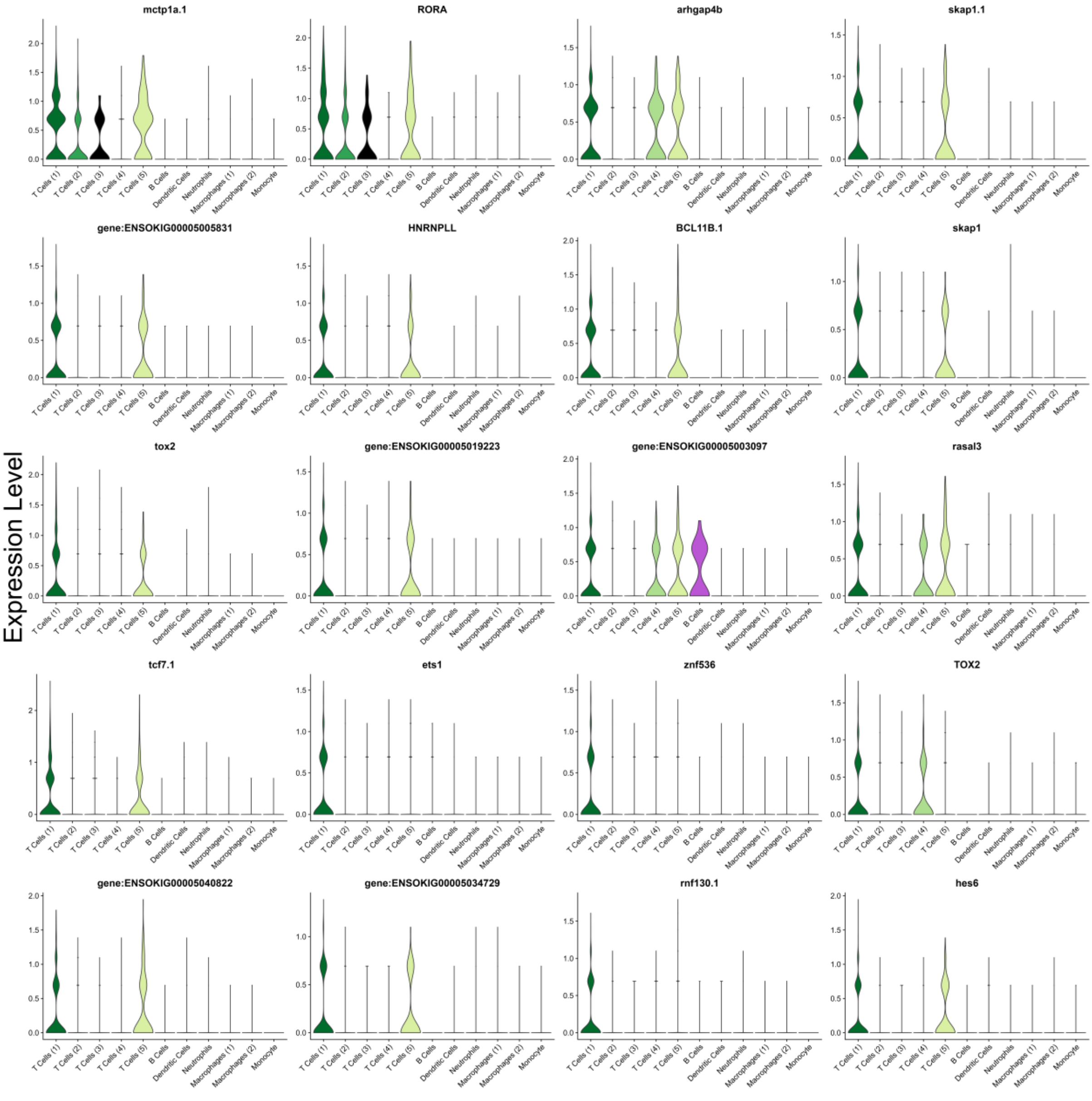

Fig.S21 Violin plots of expression levels (based on the SCT assay) for the top 20 significant (adjusted p-value < 0.05) marker genes for the T cells (1) cluster (based on log-scale two-fold change in expression) of the coho salmon immune cells only data subset (for UMAP see Fig.3d).

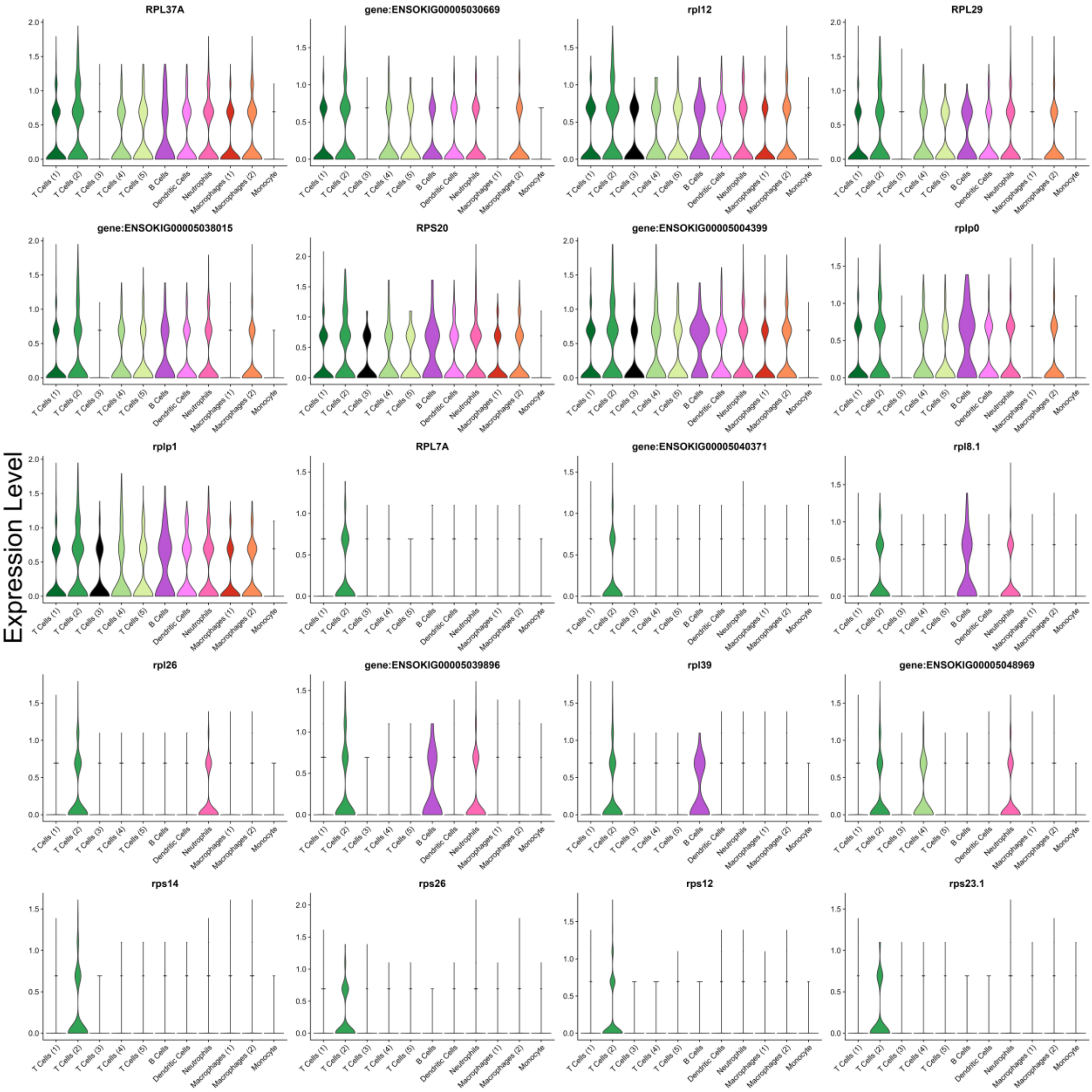

Fig.S22 Violin plots of expression levels (based on the SCT assay) for the top 20 significant (adjusted p-value < 0.05) marker genes for the T cells (2) cluster (based on log-scale two-fold change in expression) of the coho salmon immune cells only data subset (for UMAP see Fig.3d).

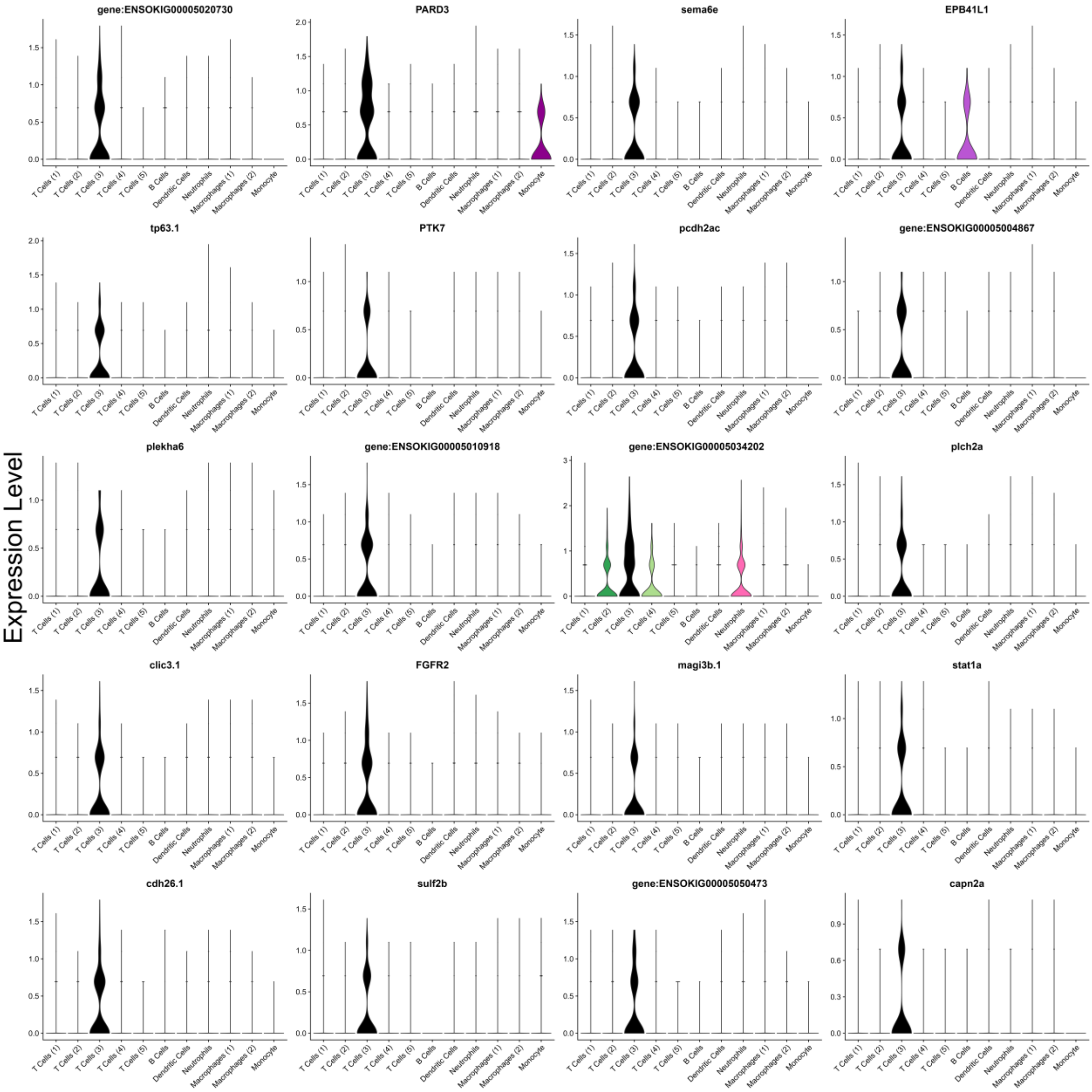

Fig.S23 Violin plots of expression levels (based on the SCT assay) for the top 20 significant (adjusted p-value < 0.05) marker genes for the T cells (3) cluster (based on log-scale two-fold change in expression) of the coho salmon immune cells only data subset (for UMAP see Fig.3d).

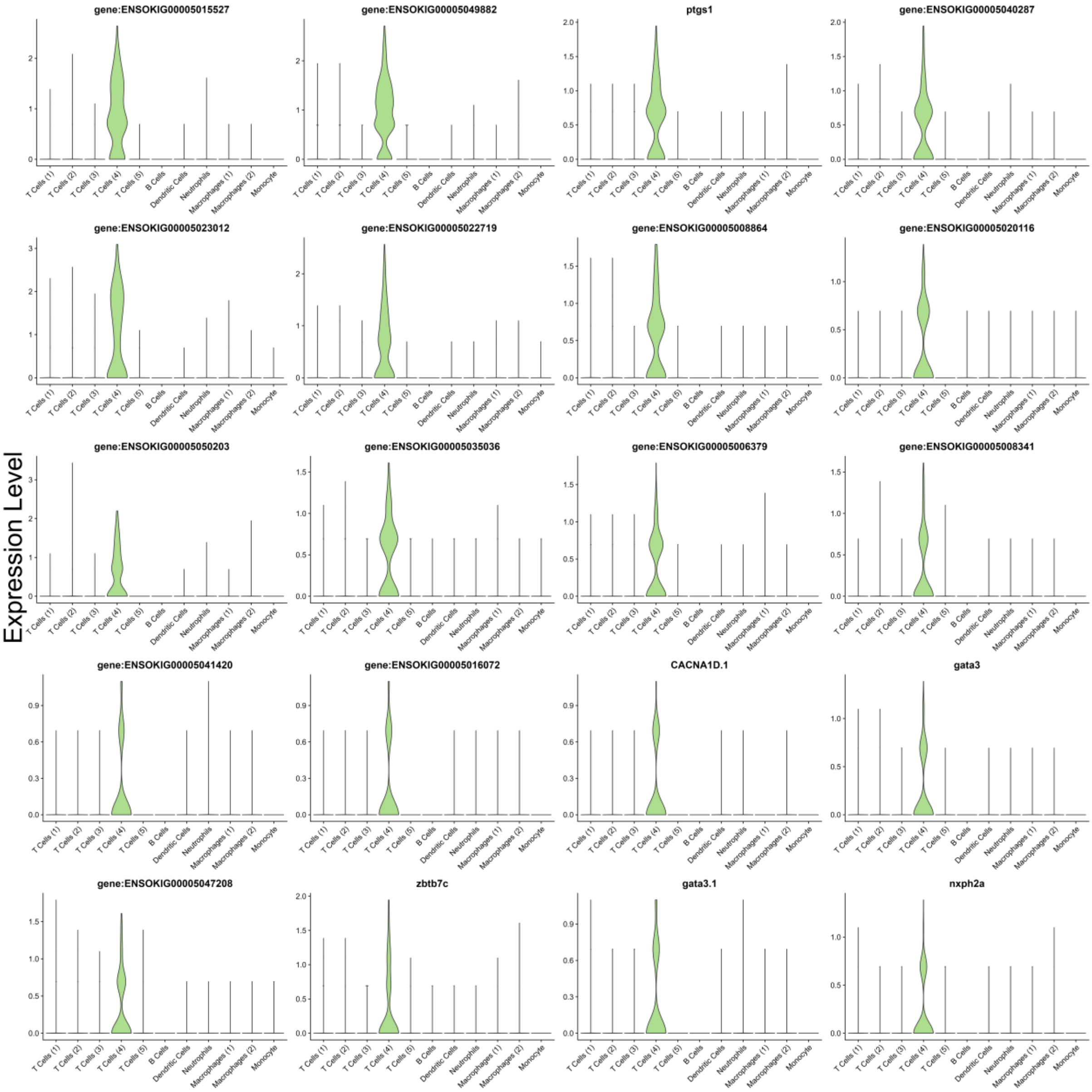

Fig.S24 Violin plots of expression levels (based on the SCT assay) for the top 20 significant (adjusted p-value < 0.05) marker genes for the T cells (4) cluster (based on log-scale two-fold change in expression) of the coho salmon immune cells only data subset (for UMAP see Fig.3d).

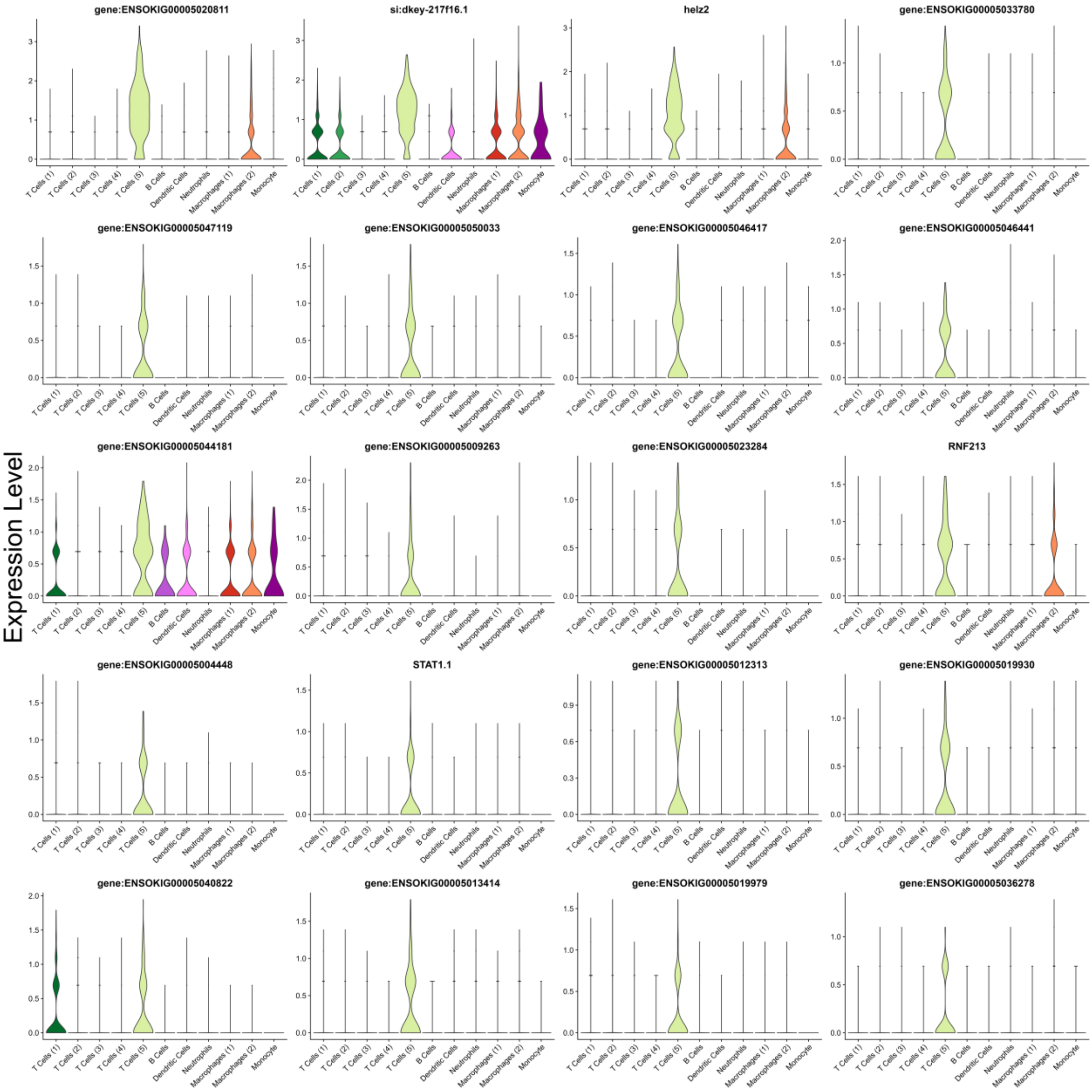

Fig.S25 Violin plots of expression levels (based on the SCT assay) for the top 20 significant (adjusted p-value < 0.05) marker genes for the T cells (5) cluster (based on log-scale two-fold change in expression) of the coho salmon immune cells only data subset (for UMAP see Fig.3d).

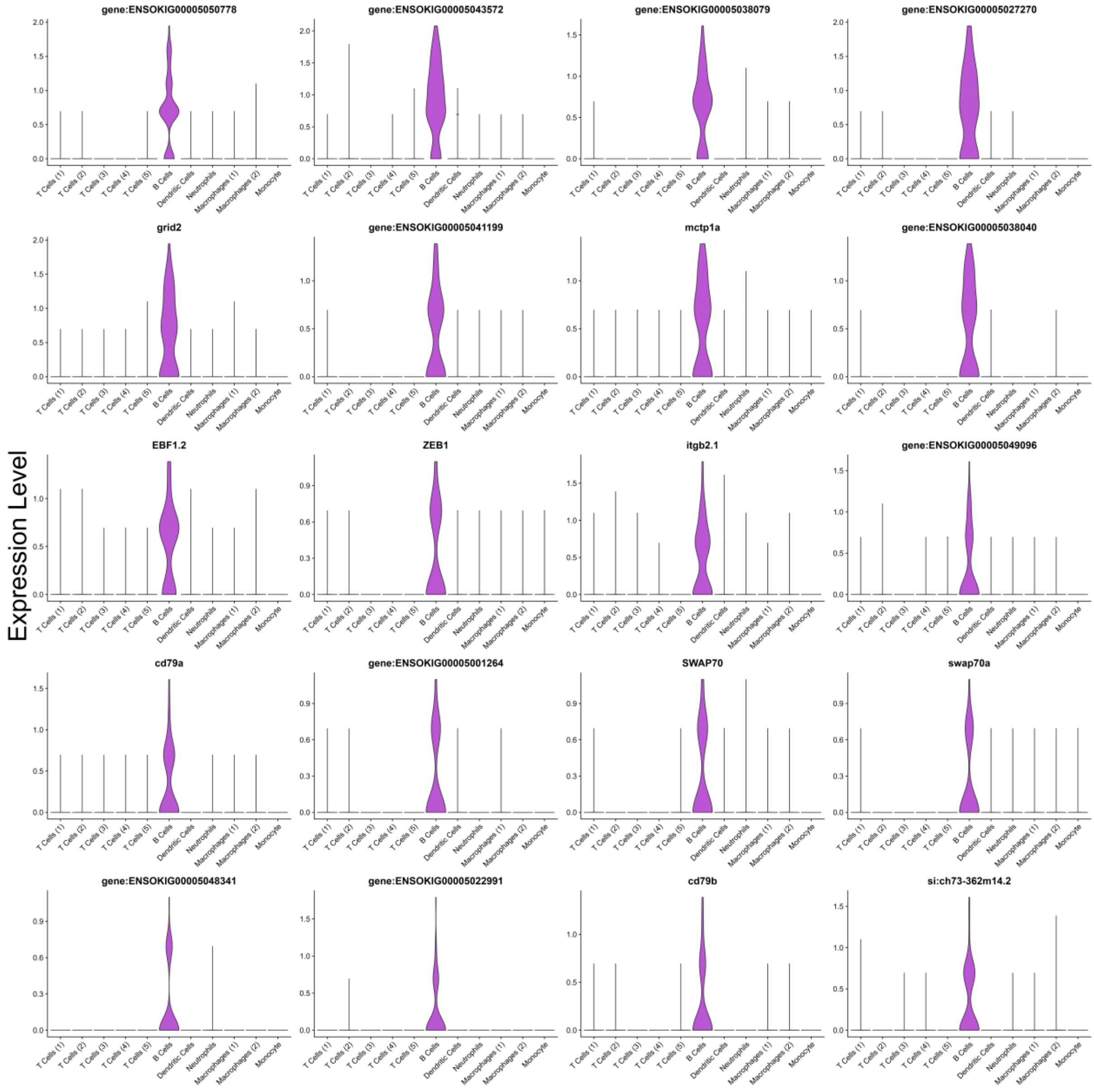

Fig.S26 Violin plots of expression levels (based on the SCT assay) for the top 20 significant (adjusted p-value < 0.05) marker genes for B cells (based on log-scale two-fold change in expression) of the coho salmon immune cells only data subset (for UMAP see Fig.3d).

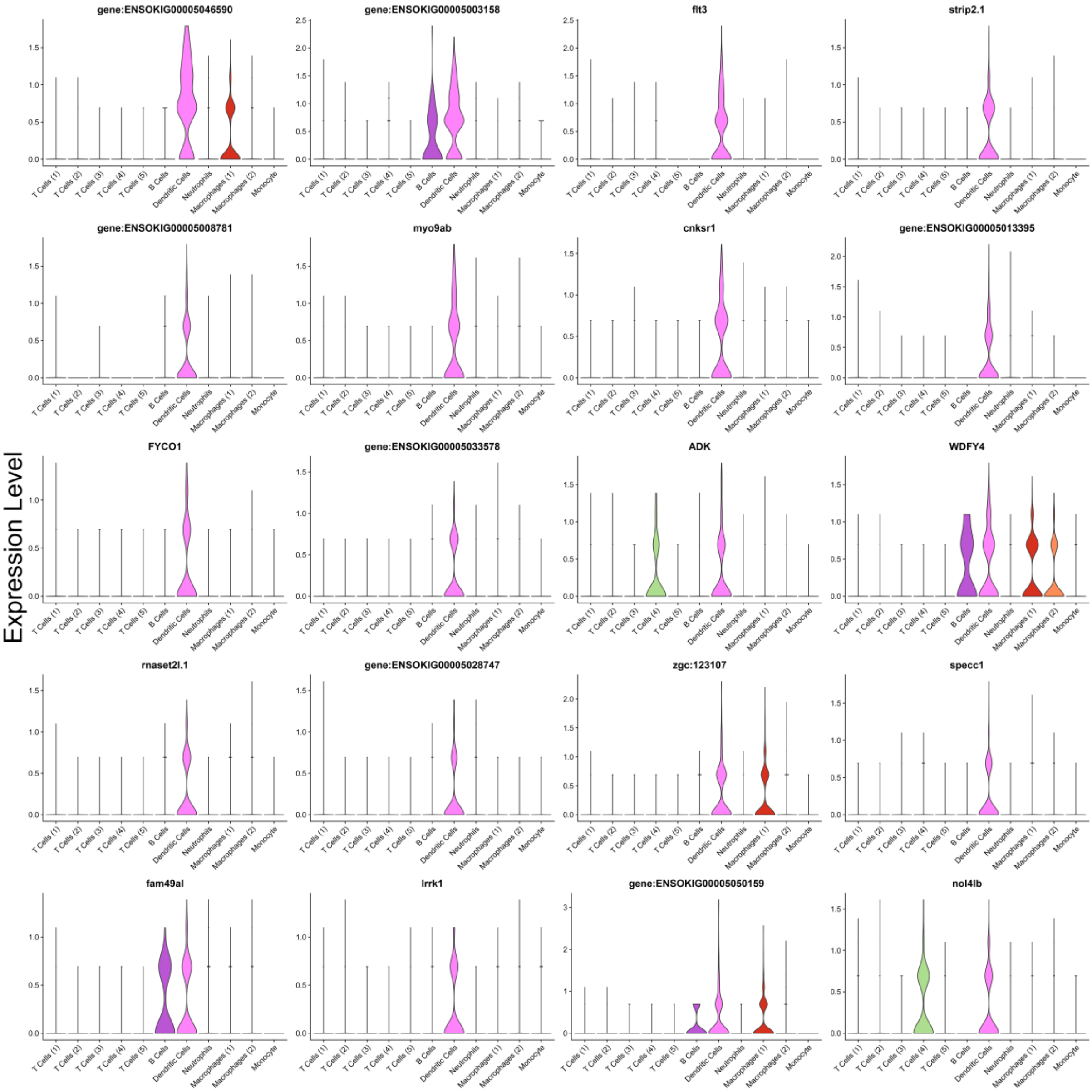

Fig.S27 Violin plots of expression levels (based on the SCT assay) for the top 20 significant (adjusted p-value < 0.05) marker genes for dendritic cells (based on log-scale two-fold change in expression) of the coho salmon immune cells only data subset (for UMAP see Fig.3d).

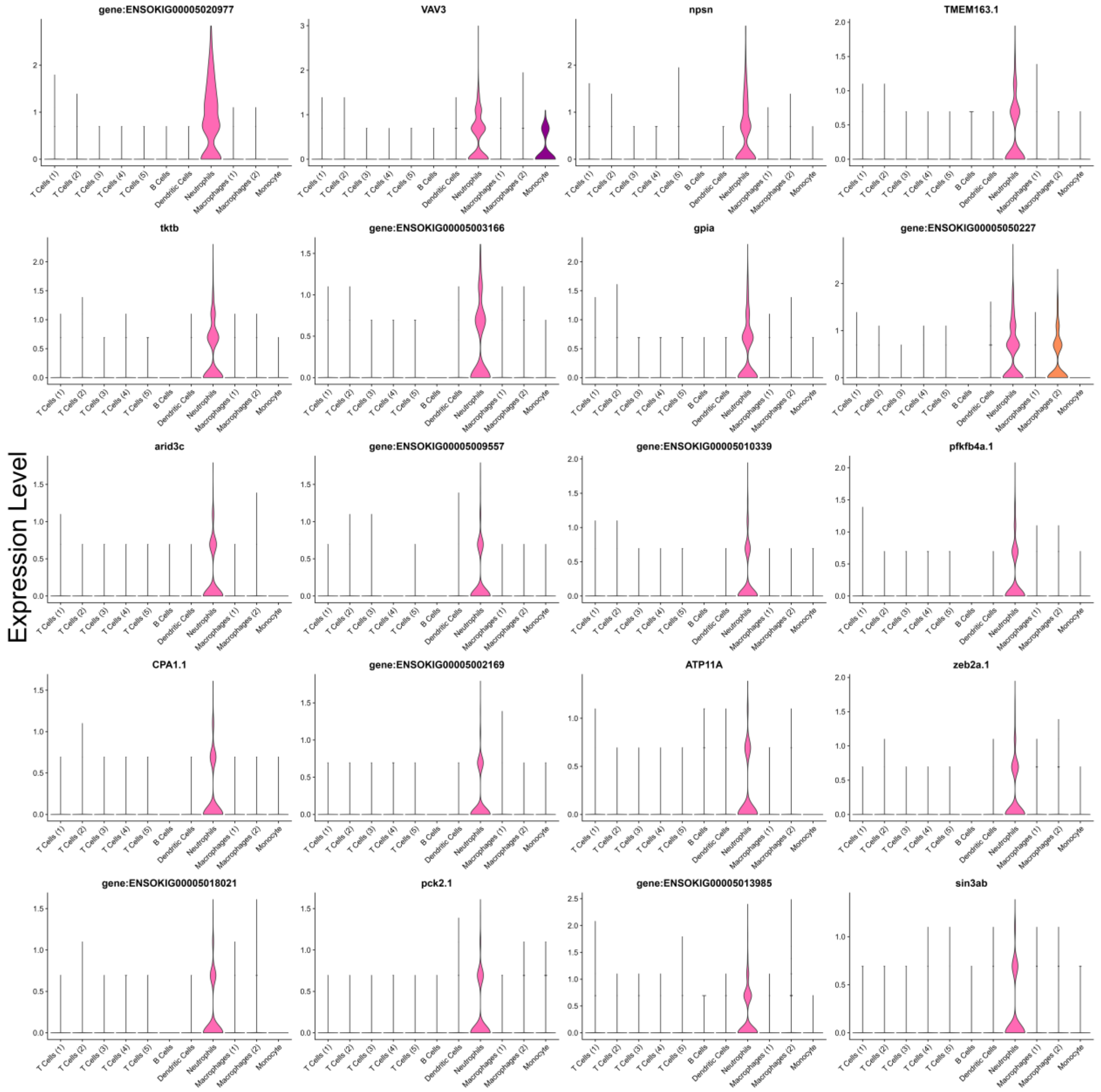

Fig.S28 Violin plots of expression levels (based on the SCT assay) for the top 20 significant (adjusted p-value < 0.05) marker genes for neutrophils (based on log-scale two-fold change in expression) of the coho salmon immune cells only data subset (for UMAP see Fig.3d).

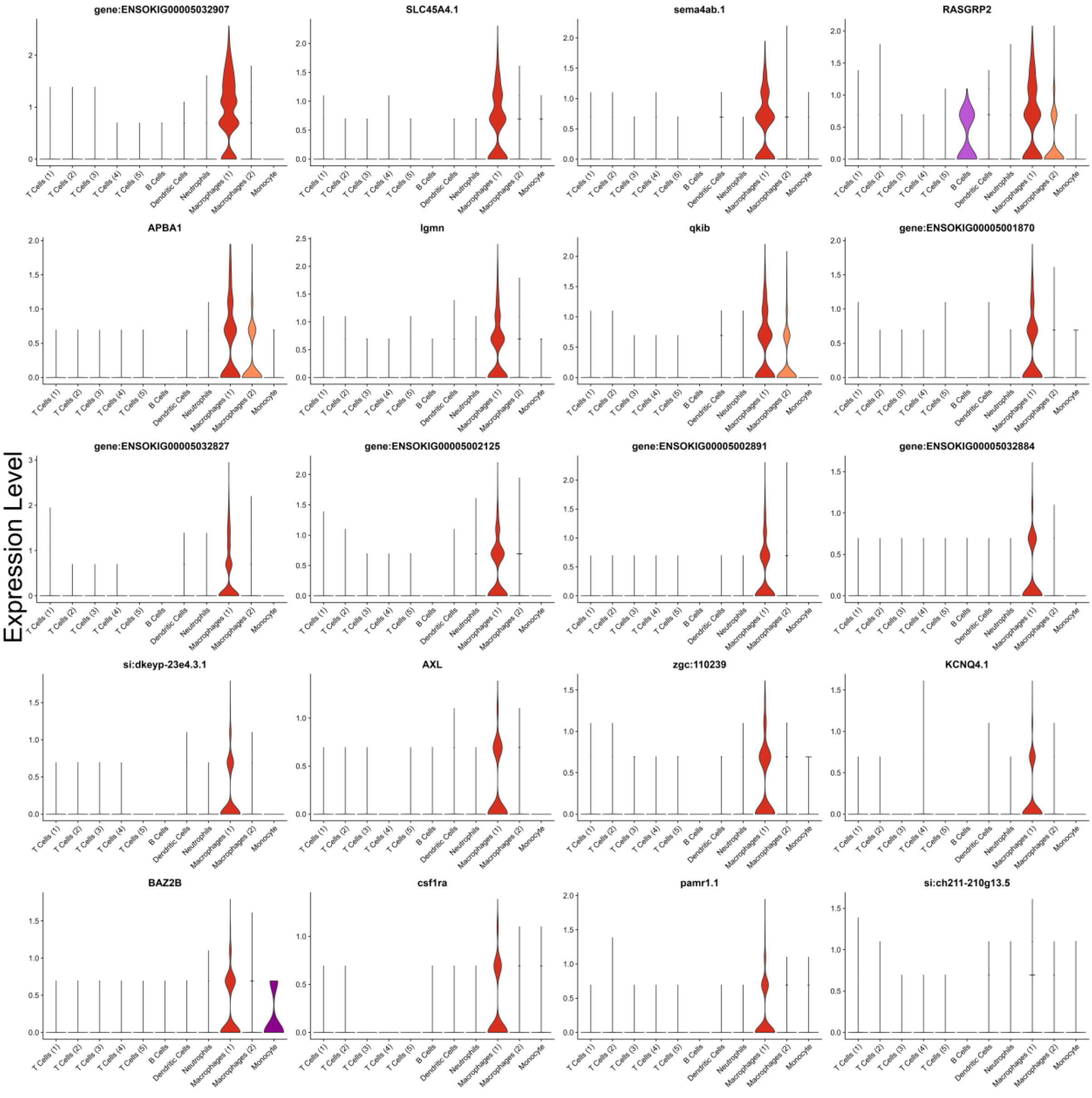

Fig.S29 Violin plots of expression levels (based on the SCT assay) for the top 20 significant (adjusted p-value < 0.05) marker genes for the macrophages (1) cluster (based on log-scale two-fold change in expression) of the coho salmon immune cells only data subset (for UMAP see Fig.3d).

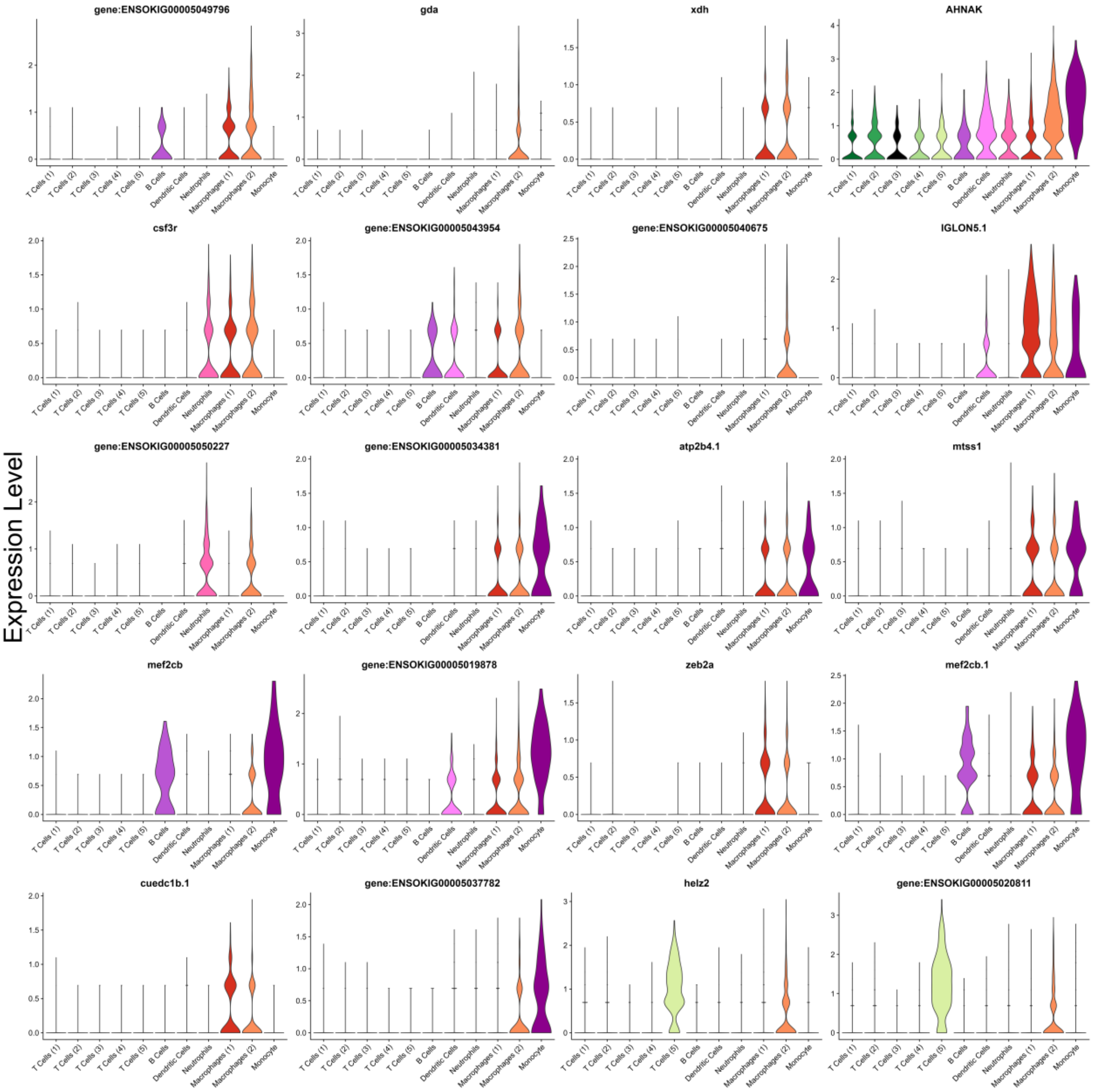

Fig.S30 Violin plots of expression levels (based on the SCT assay) for the top 20 significant (adjusted p-value < 0.05) marker genes for the macrophages (2) cluster (based on log-scale two-fold change in expression) of the coho salmon immune cells only data subset (for UMAP see Fig.3d).

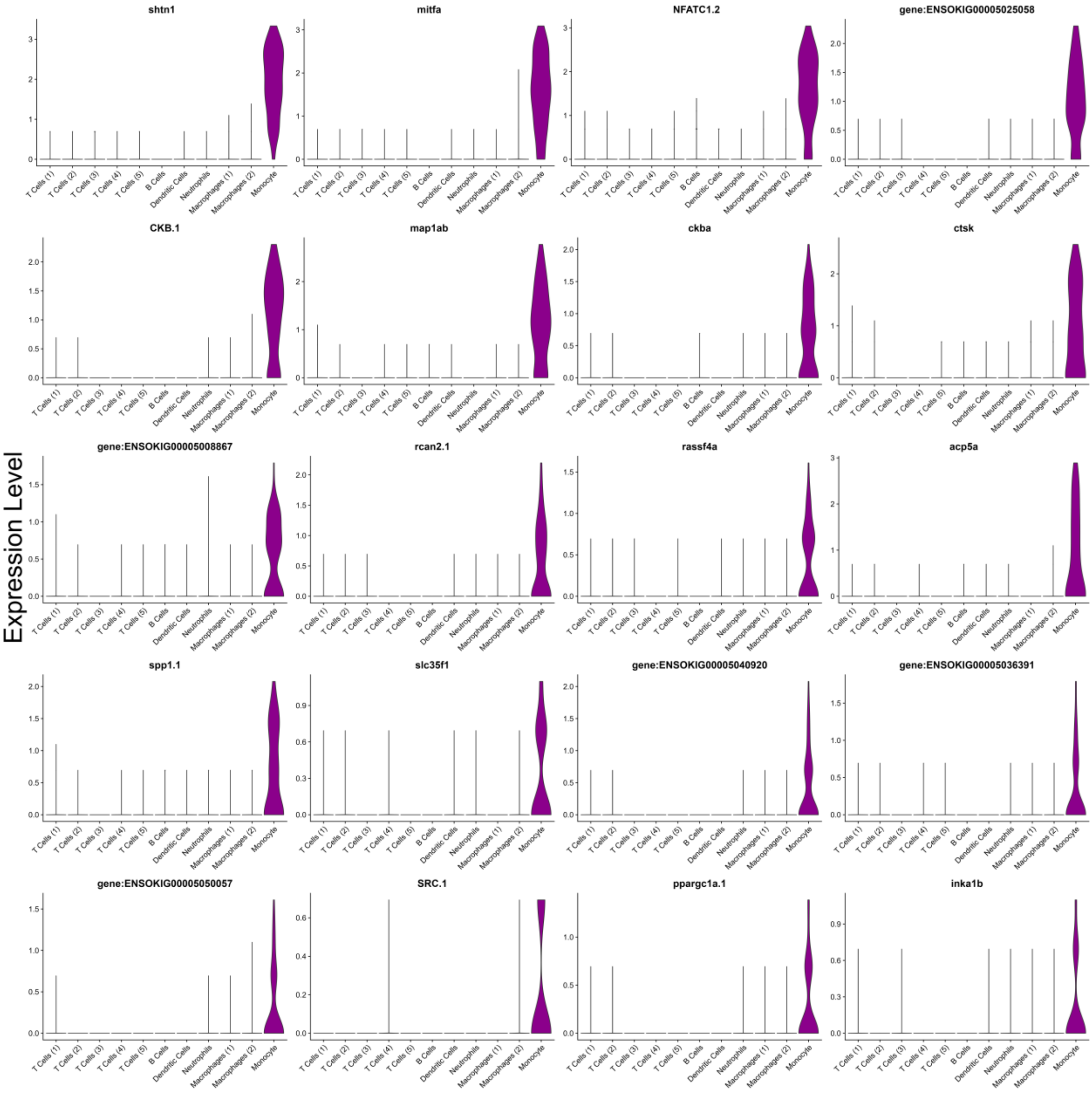

Fig.S31 Violin plots of expression levels (based on the SCT assay) for the top 20 significant (adjusted p-value < 0.05) marker genes for monocytes (based on log-scale two-fold change in expression) of the coho salmon immune cells only data subset (for UMAP see Fig.3d).

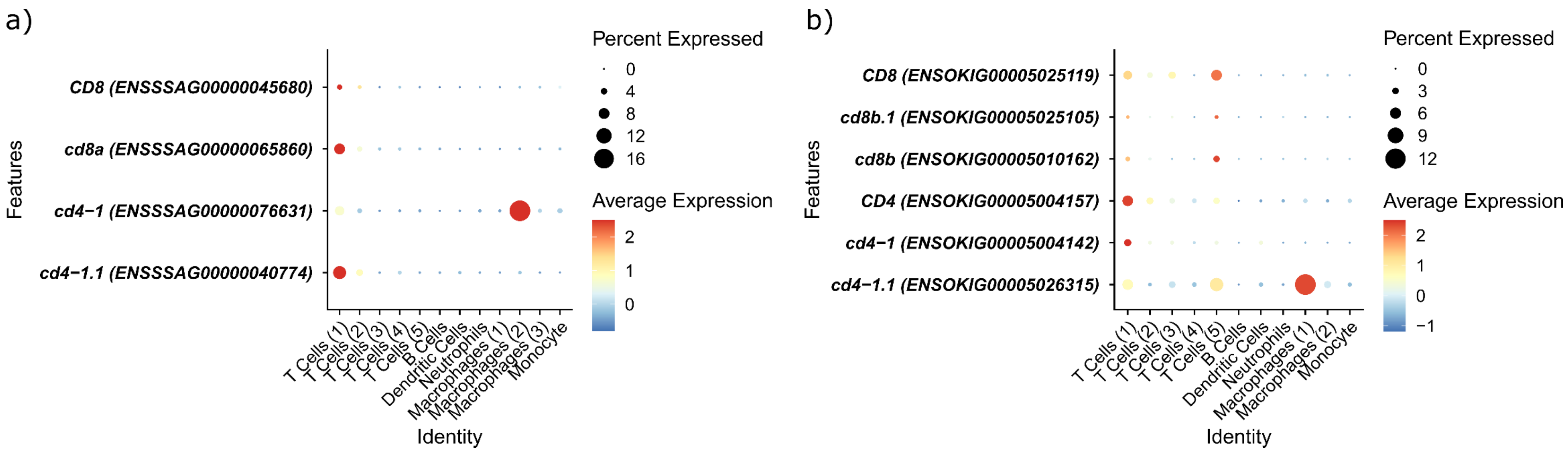

Fig.S32 Expression of CD4 and CD8 paralogs in the immune cell subclusters identified within a) Atlantic salmon and b) coho salmon.

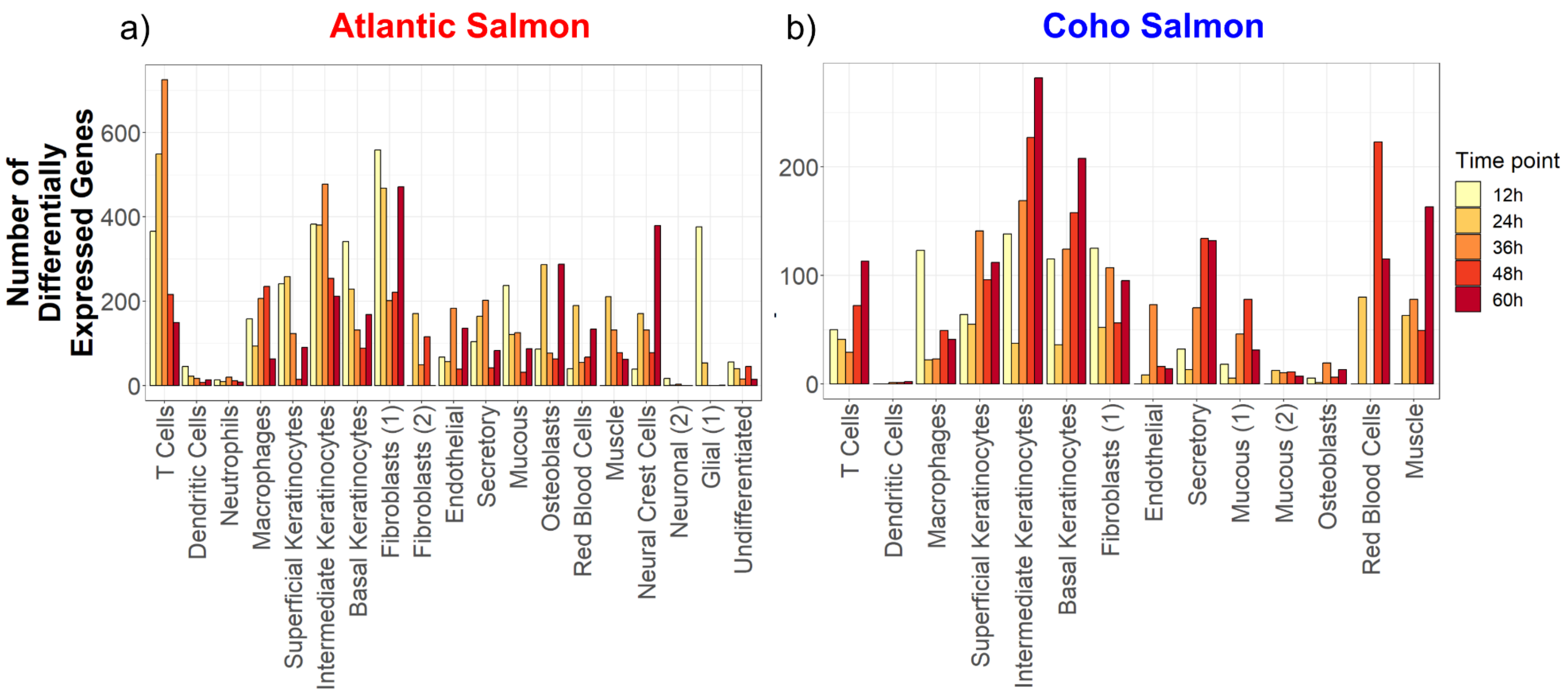

Fig.S33 Number of genes detected as differentially expressed between the control samples and each of the infected time points (12h, 24h, 36h, 48h, 60h) for each cell type for a) Atlantic salmon and b) coho salmon.

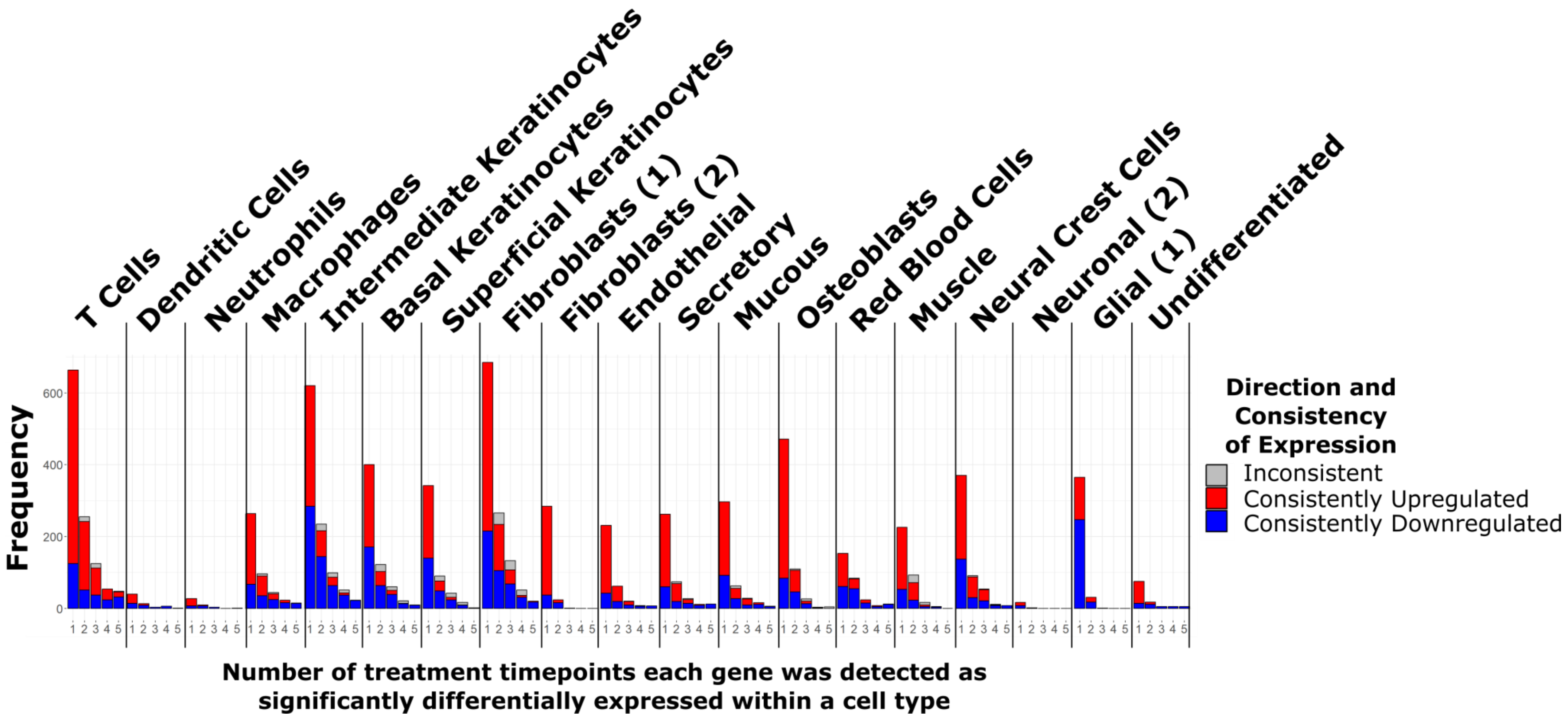

Fig.S34 Number of times each gene was detected as differentially expressed (1-5 time points) between any of the treatment time points and the control sample for a given cell type detected within the Atlantic salmon samples. Counts are coloured by whether differentially expressed genes were either consistently upregulated (red), downregulated (blue), or were detected within the same cell type as being significantly upregulated and downregulated at different time points (grey).

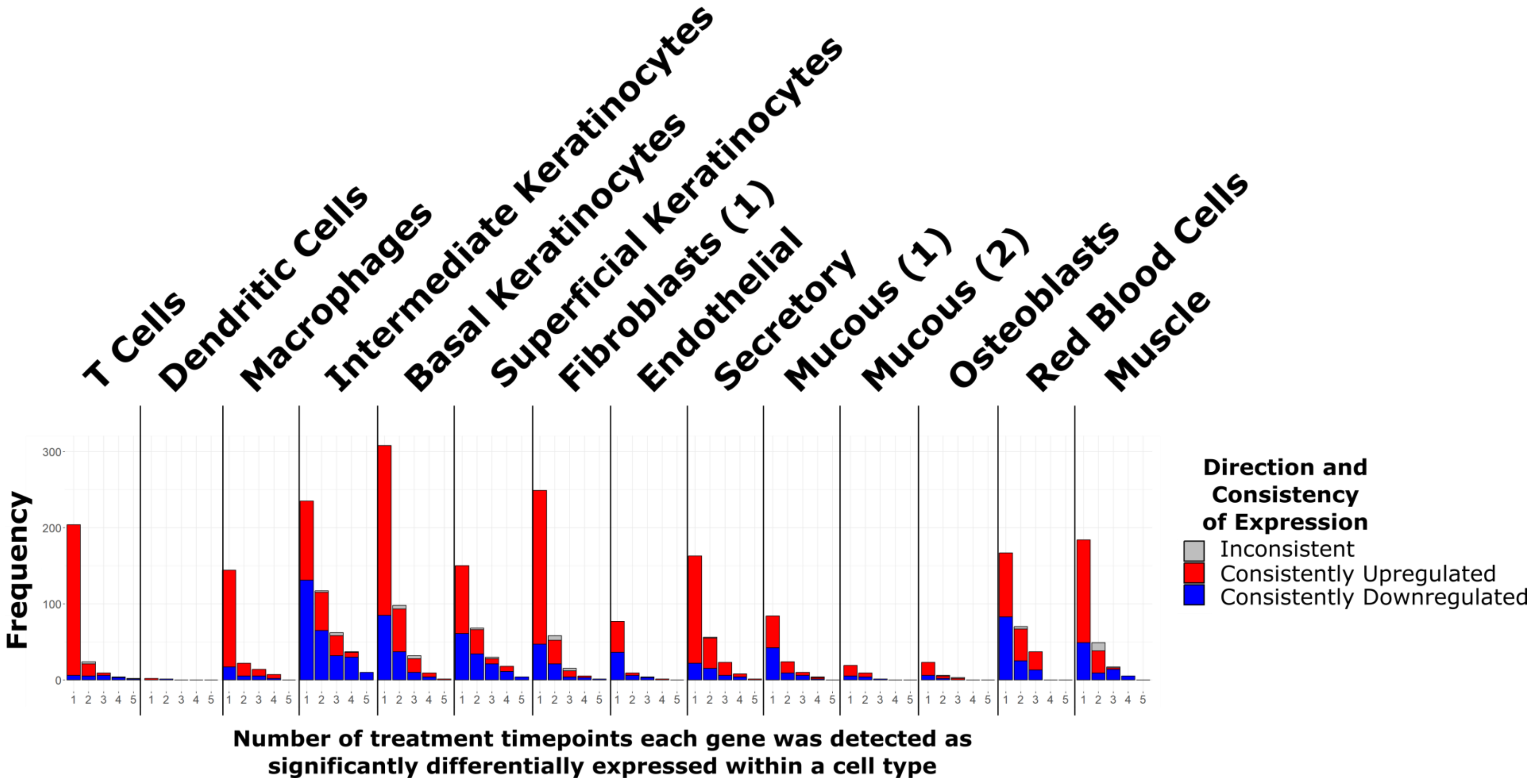

Fig.S35 Number of times each gene was detected as differentially expressed (1-5 time points) between any of the treatment time points and the control sample for a given cell type detected within the coho salmon samples. Counts are coloured by whether differentially expressed genes were either consistently upregulated (red), downregulated (blue), or were detected within the same cell type as being significantly upregulated and downregulated at different time points (grey).

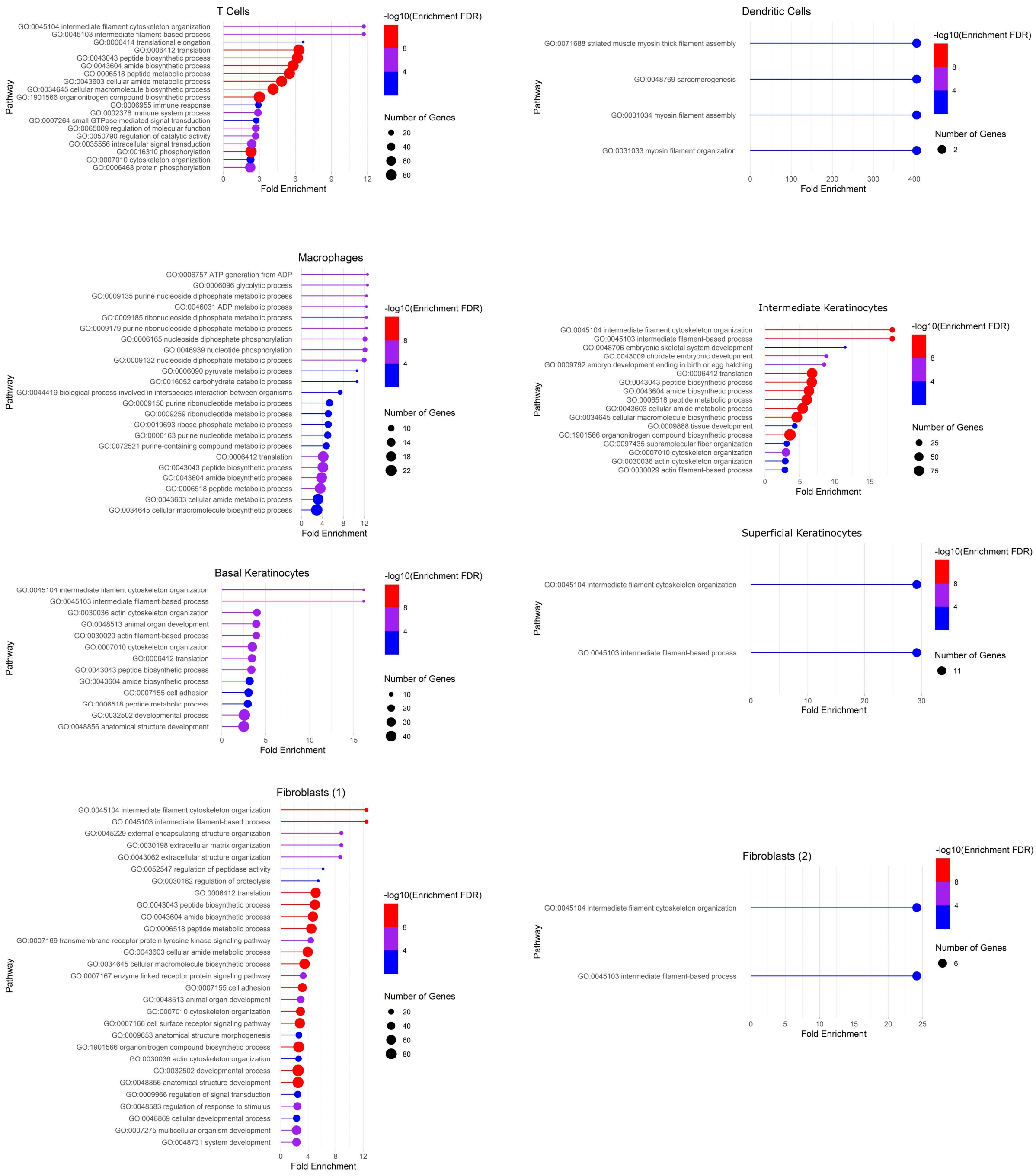

Fig.S36 Significantly (FDR-adjusted p-value < 0.001) enriched Biological GO terms for each Atlantic salmon cell type based on the significantly differentially expressed genes detected between the control samples and any of the five treatment time points (12h, 24h, 36h, 48h, 60h). Please note that no significant GO terms were detected for Endothelial, Glial (1), Mucous, Neuronal (2), and Neutrophils. (Continued on next page.)

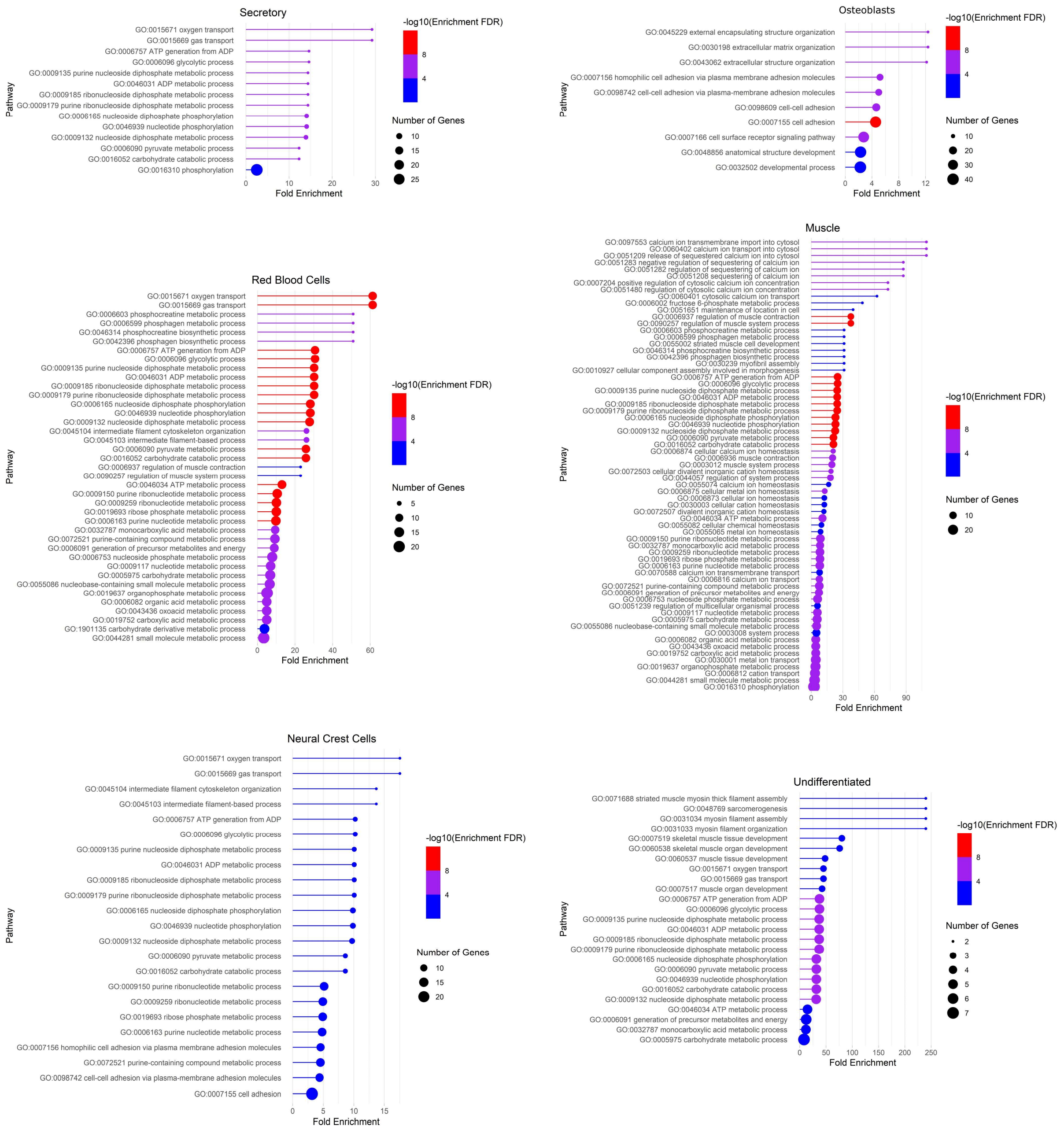

Fig.S36 Continued.

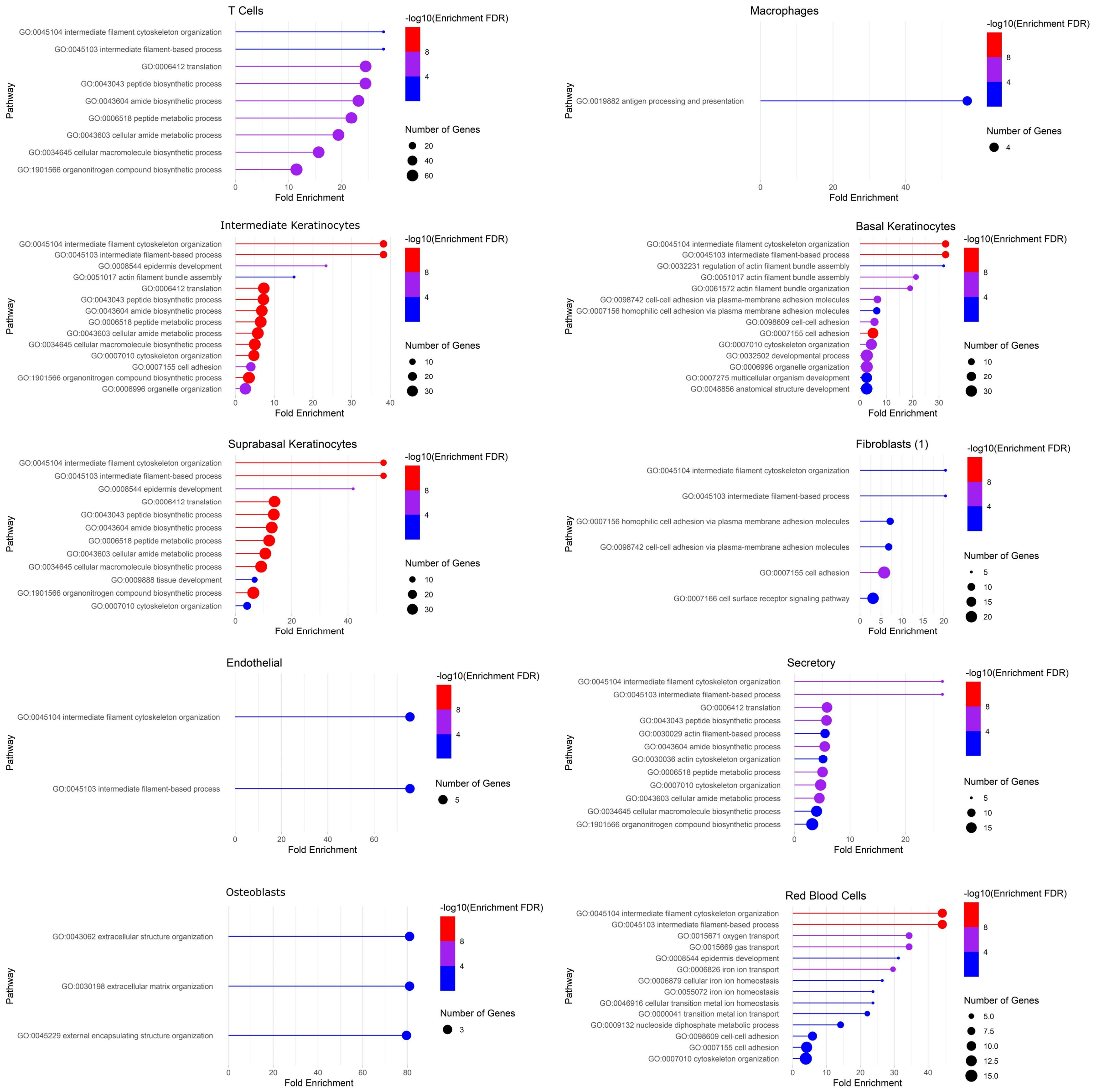

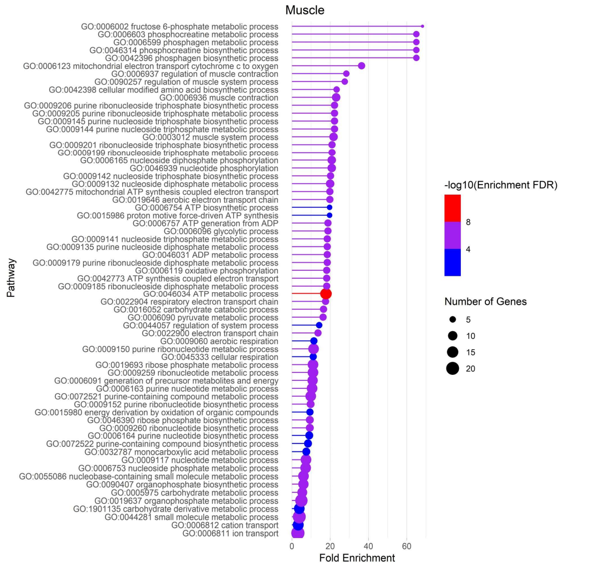

Fig.S37 Significantly (FDR-adjusted p-value < 0.001) enriched Biological GO terms for each coho salmon cell type based on the significantly differentially expressed genes detected between the control samples and any of the five treatment time points (12h, 24h, 36h, 48h, 60h). Please note that no significant GO terms were detected for Mucous (1), Mucous (2), and Dendritic Cells.

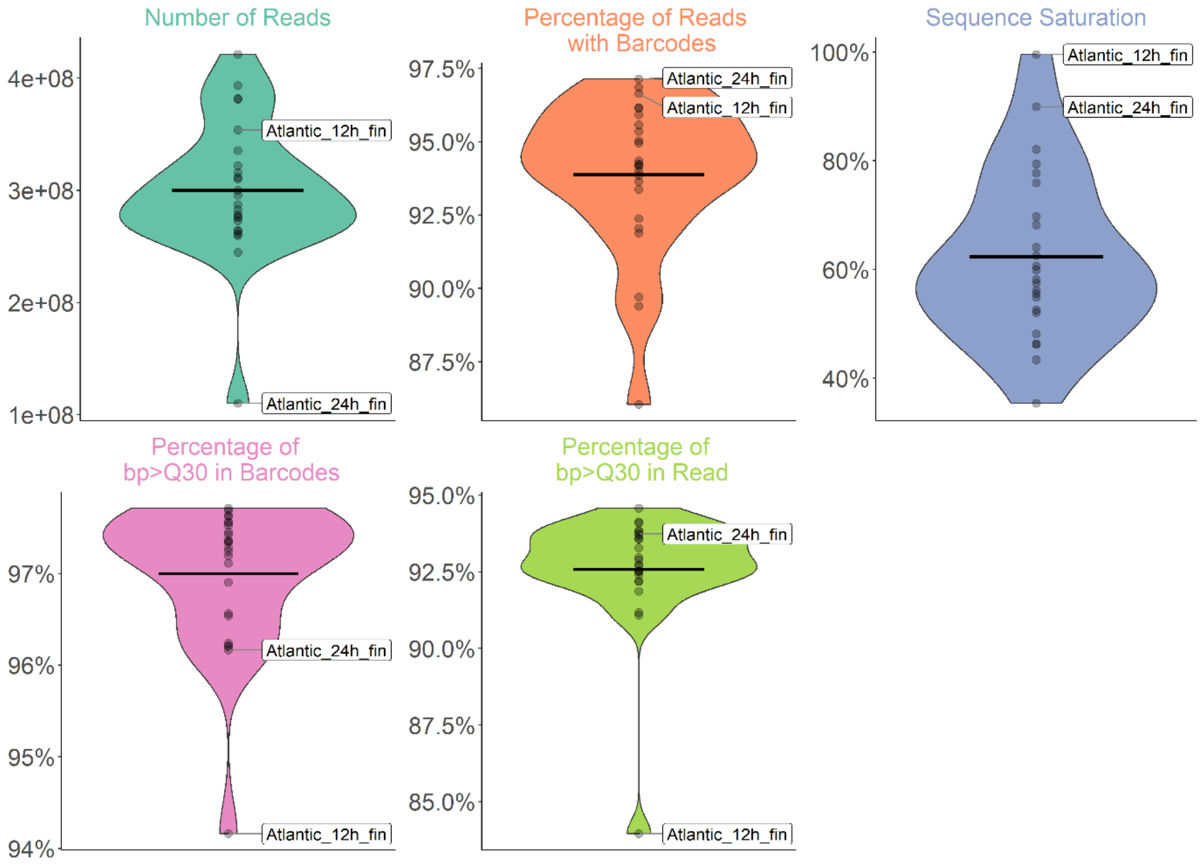

Fig.S38 Summary statistics for all Atlantic and coho salmon libraries. Samples which were removed in downstream quality control filtering in Seurat are highlighted (i.e., Atlantic_12h_fin, Atlantic_24_fin).

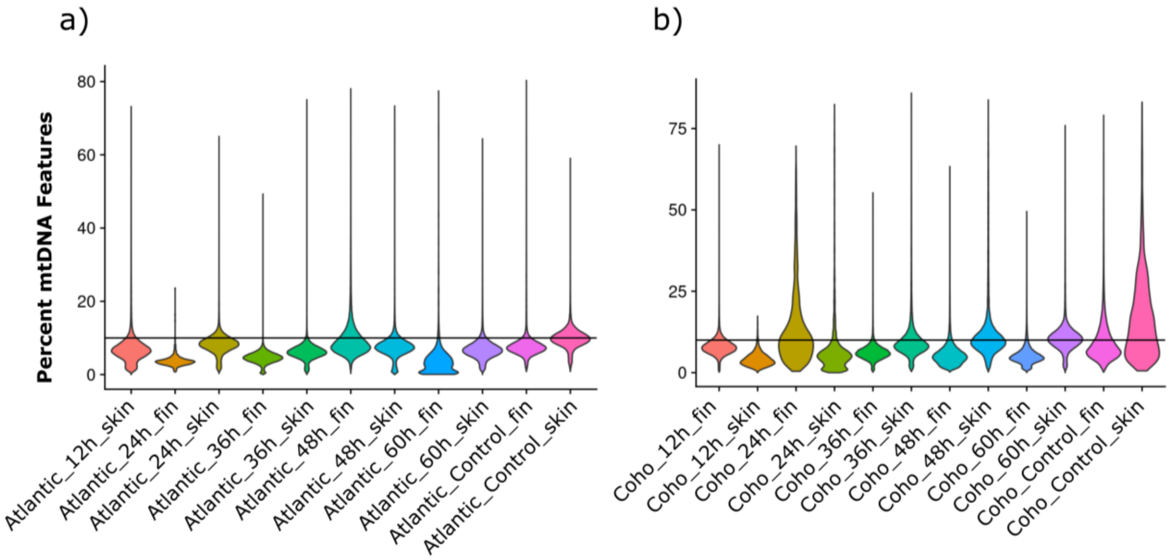

Fig.S39 Percent of UMIs identified as mtDNA features for a) Atlantic salmon and b) coho salmon samples, with the 10% maximum threshold used for subsequent filtering indicated by a horizontal black line.

Atlantic_Control_fin

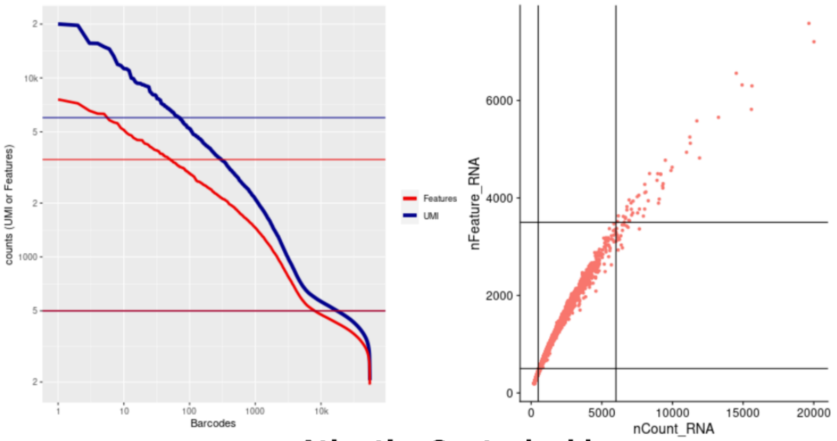

Atlantic_Control_skin

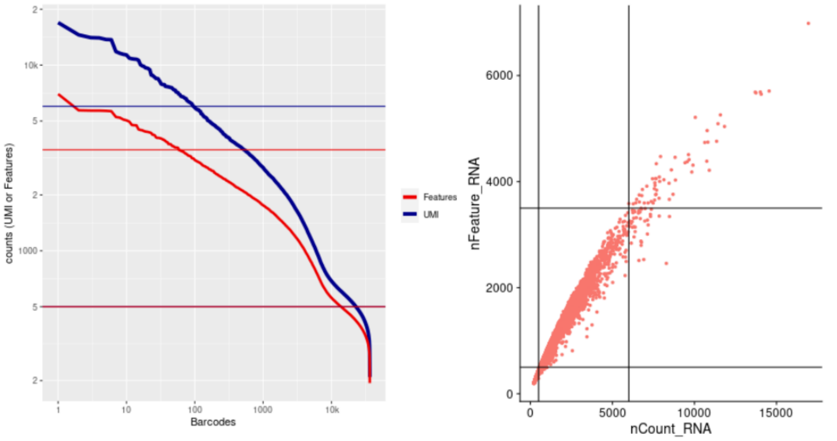

Atlantic_12h_skin

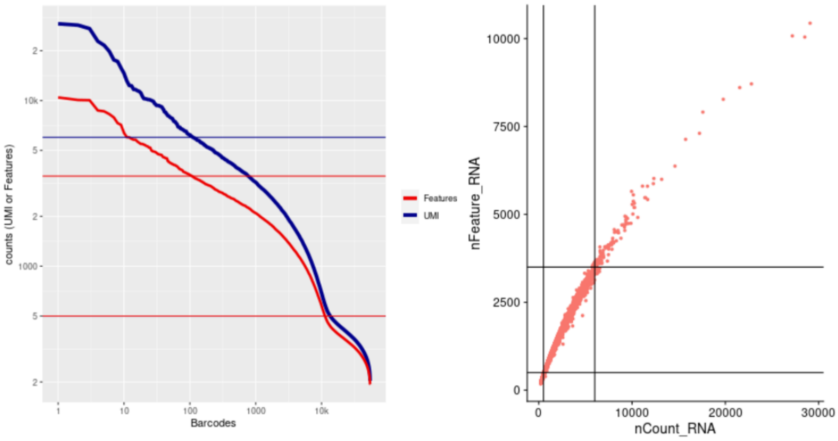

Atlantic_24h_fin

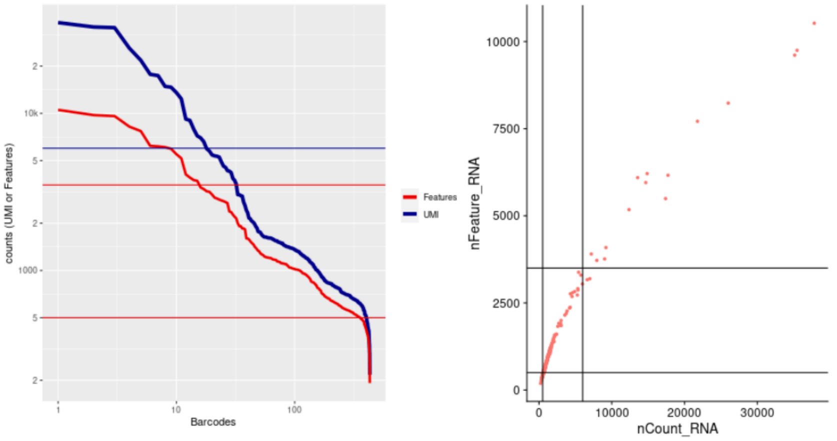

Atlantic_24h_skin

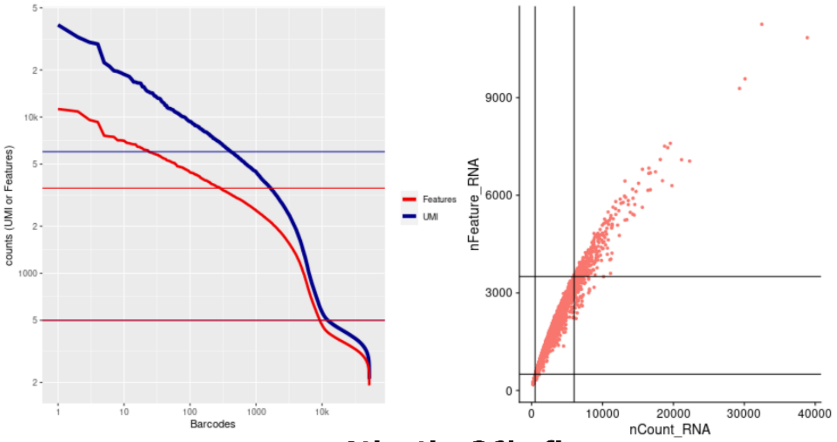

Atlantic_36h_fin

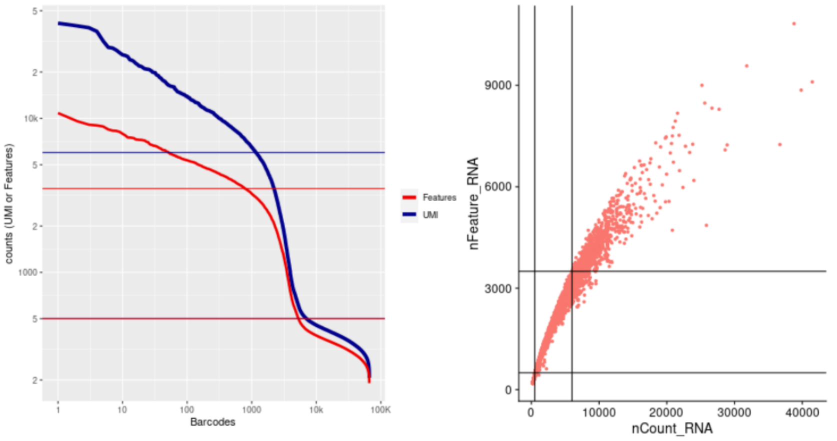

Atlantic_36h_skin

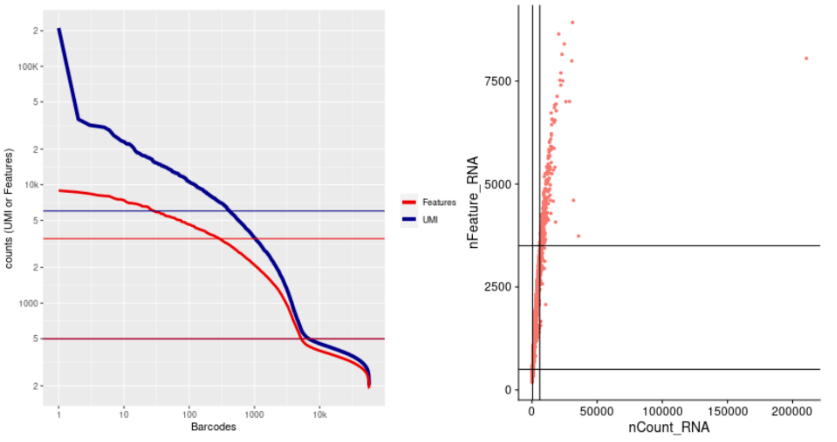

Atlantic_48h_fin

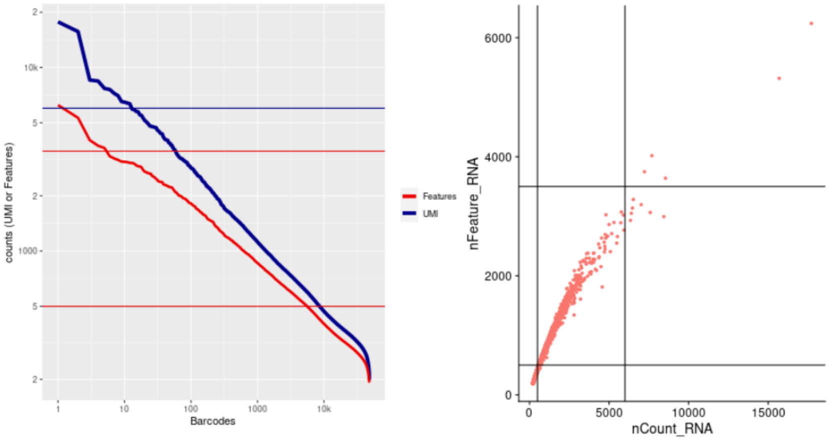

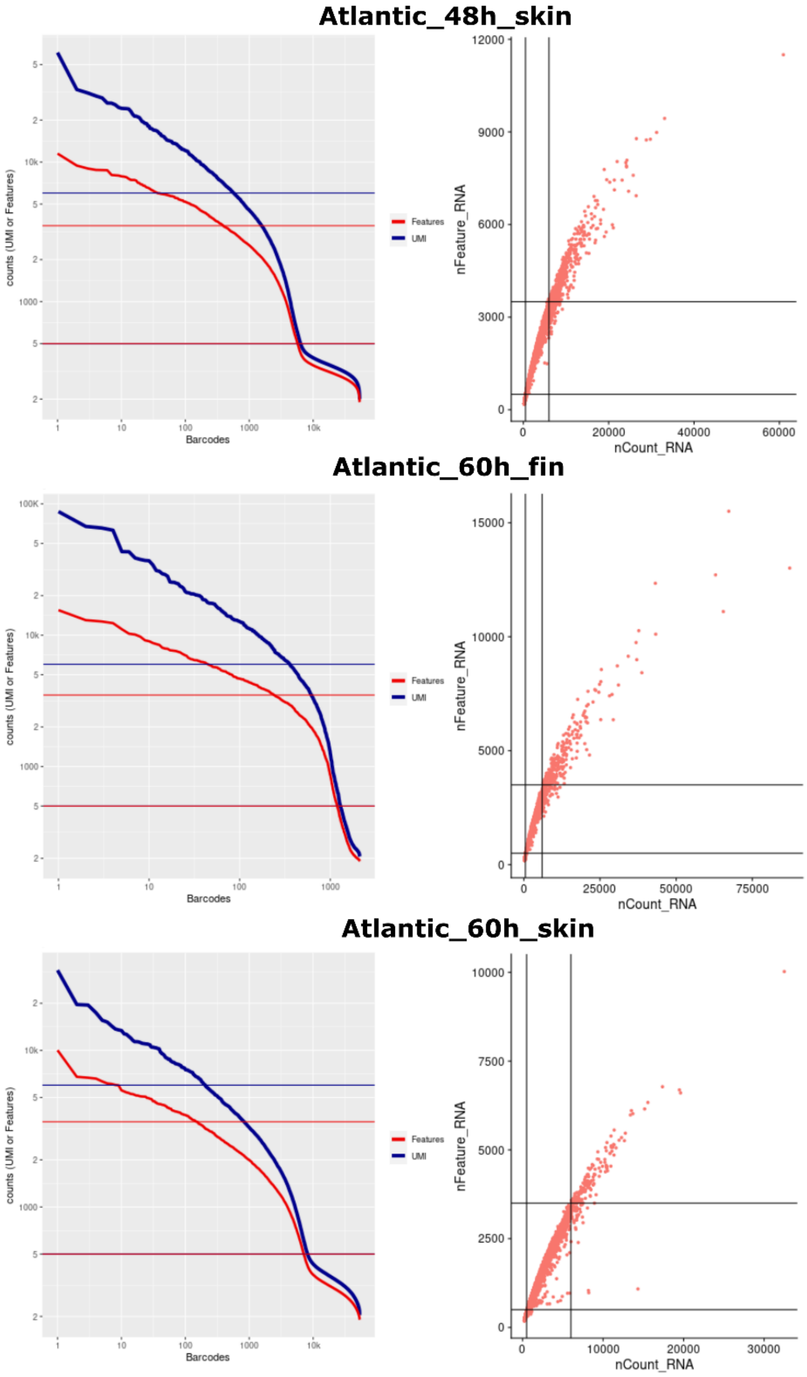

Fig.S40 UMI and feature counts per cell barcode and feature counts vs. UMI counts for each Atlantic salmon sample.

Coho_Control_fin

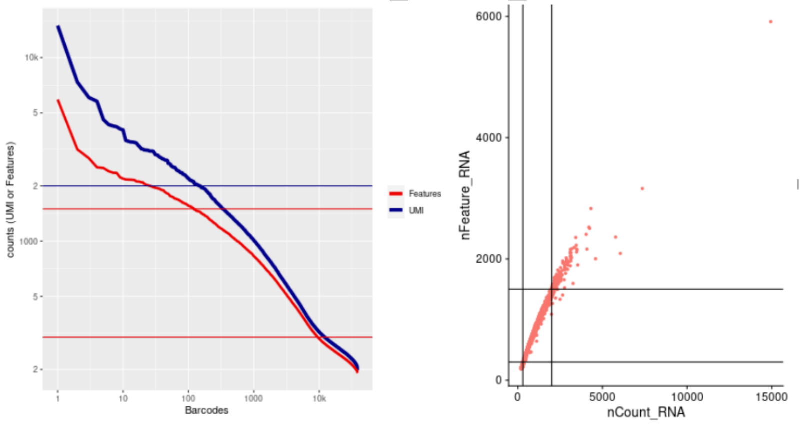

Coho_Control_skin

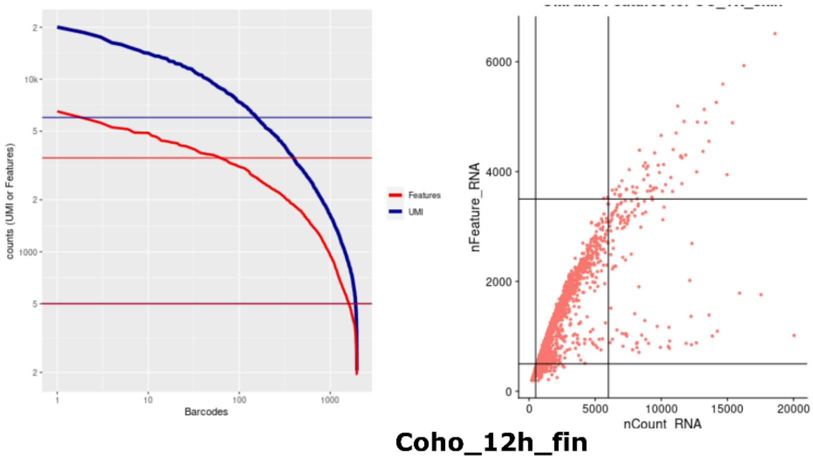

Coho_12h_fin

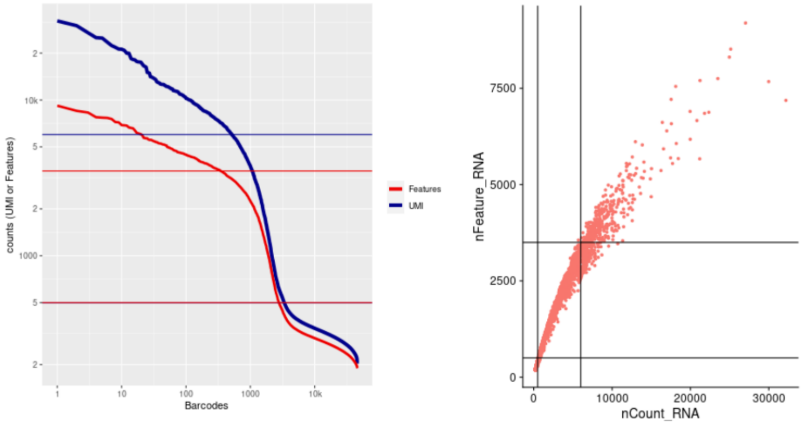

Coho_12h_skin

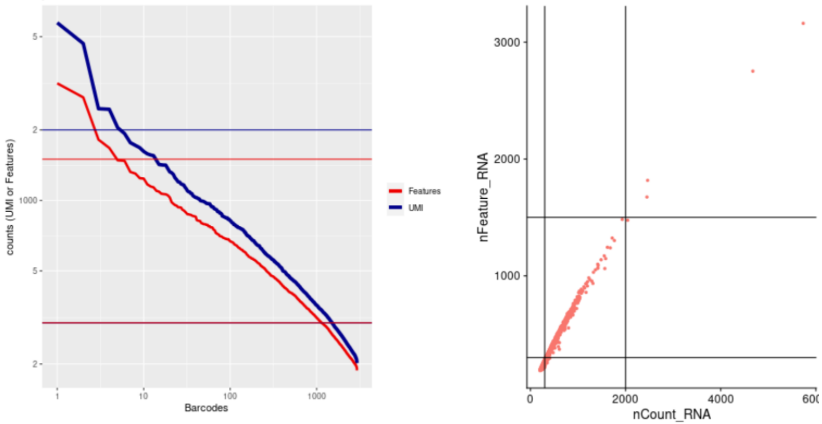

Coho_24h_fin

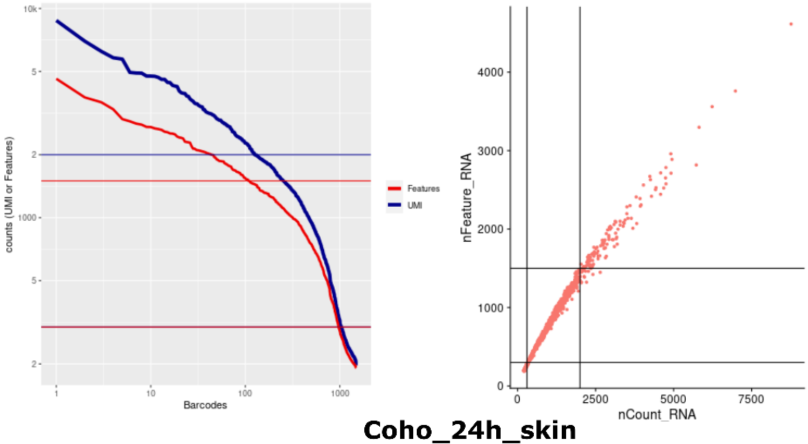

Coho_24h_skin

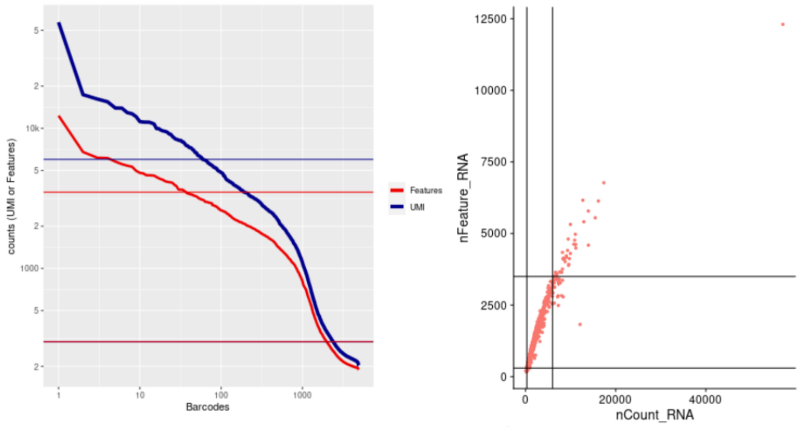

Coho_36h_fin

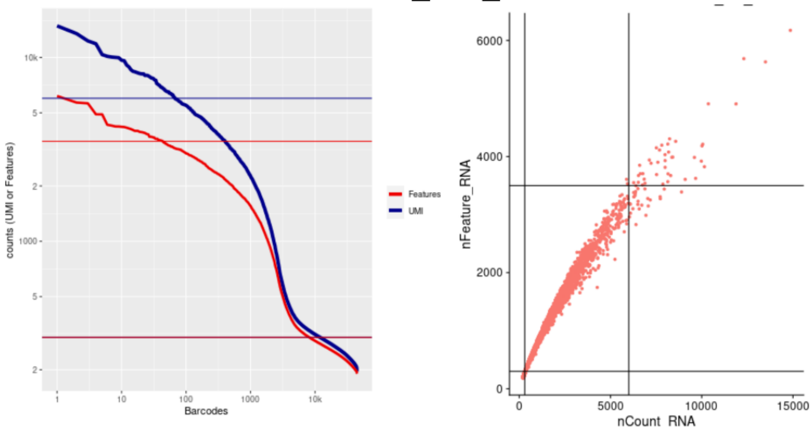

Coho_36h_skin

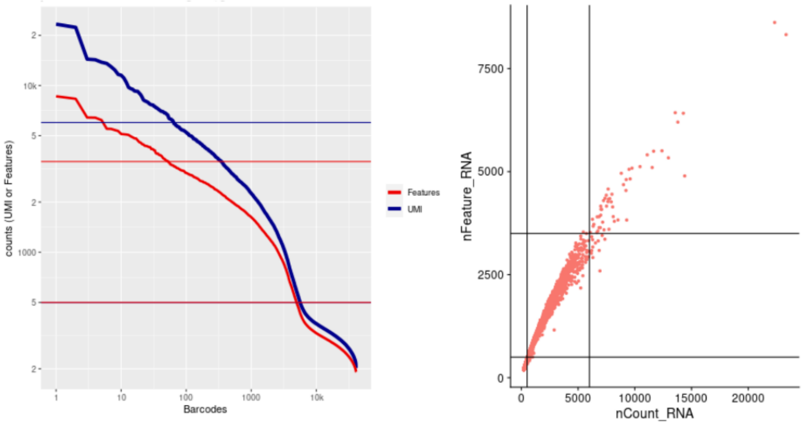

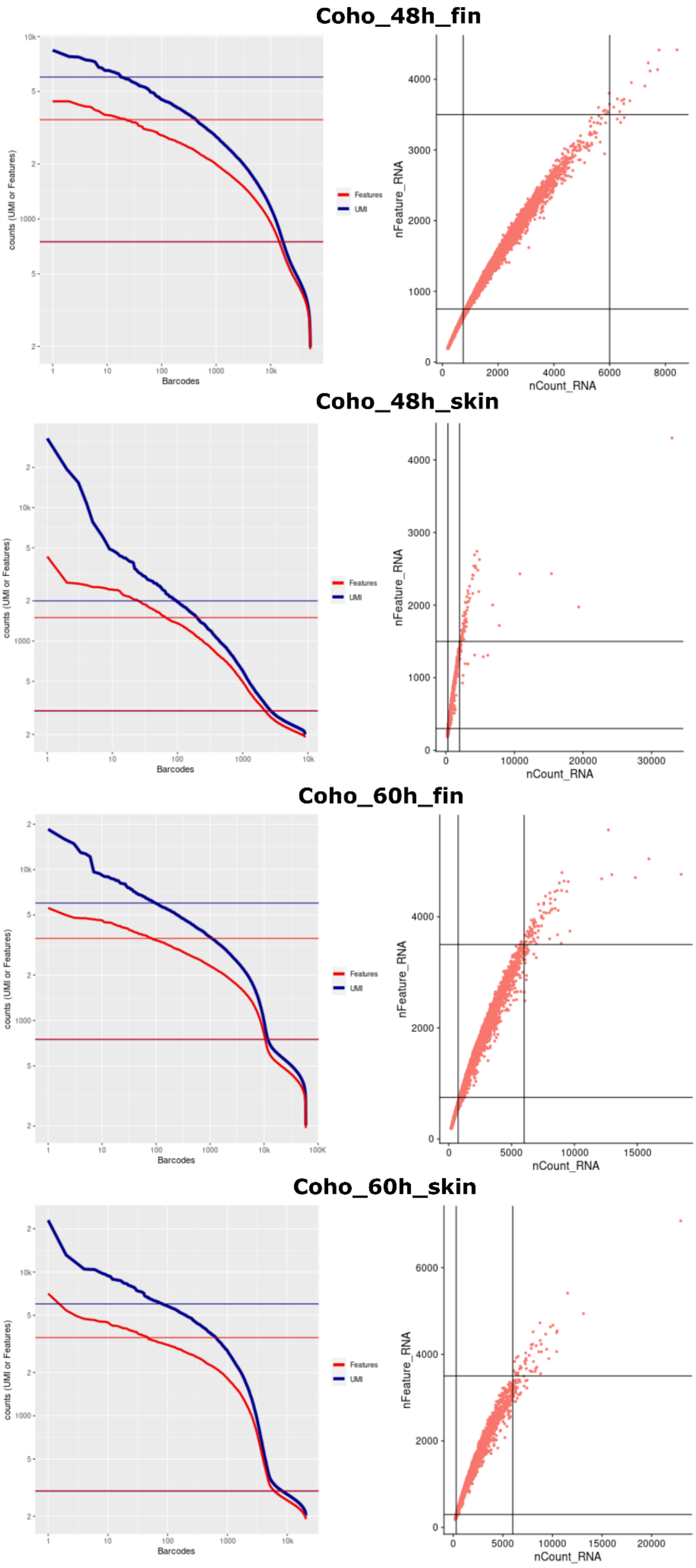

Fig.S41 UMI and feature counts per cell barcode and feature counts vs. UMI counts for each coho salmon sample.

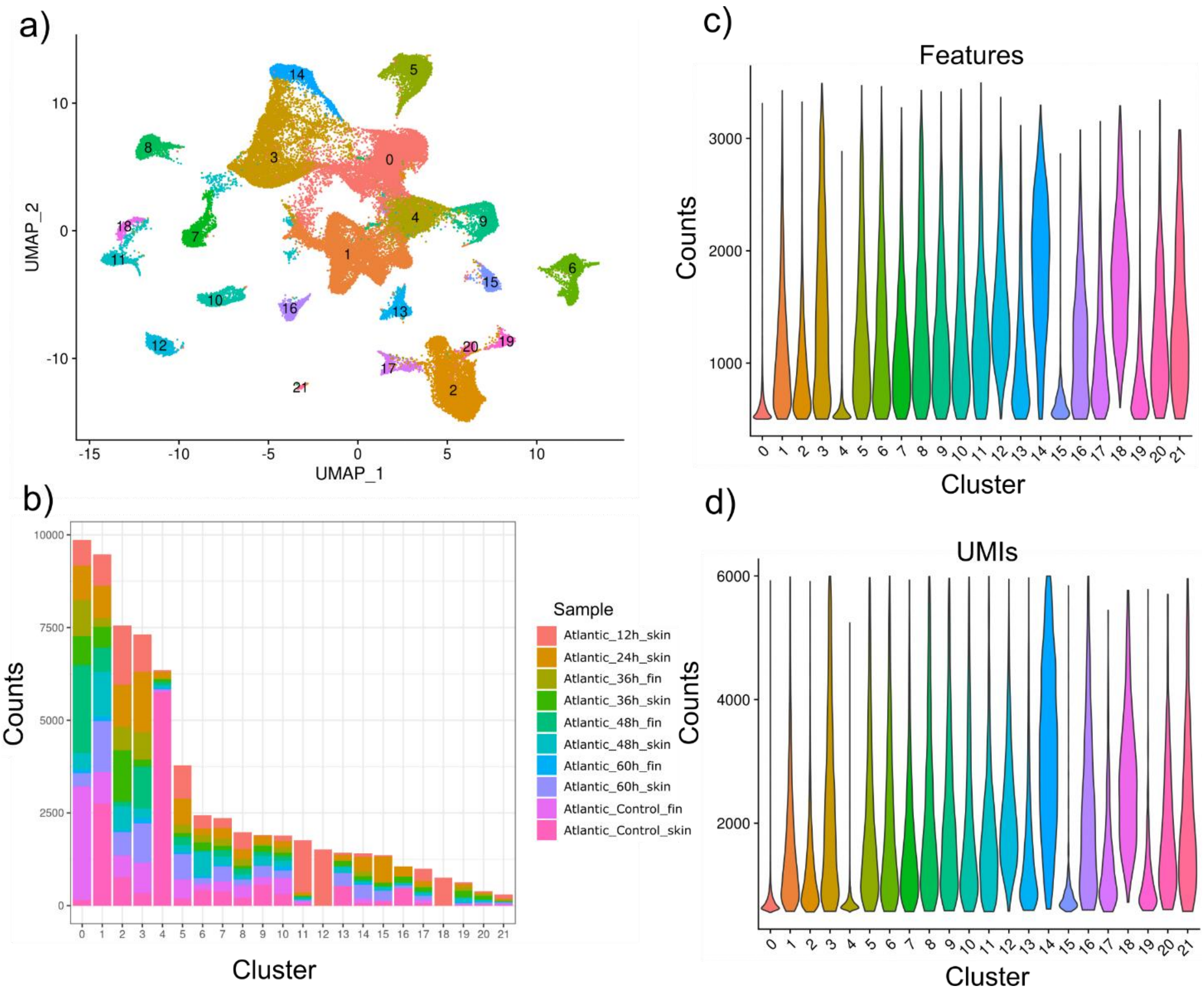

Fig.S42 Cell clusters after initial integration of Atlantic salmon samples: a) UMAP, b) number of cells per cluster per sample, c) Violin plot of the distribution of feature counts per cluster, d) Violin plot of the distribution of UMI counts per cluster. Note that clusters 0 and 4 were removed from subsequent analysis due to low average UMI/feature counts. Cluster 4 was also found almost exclusively in a single sample (Atlantic_Control_skin).

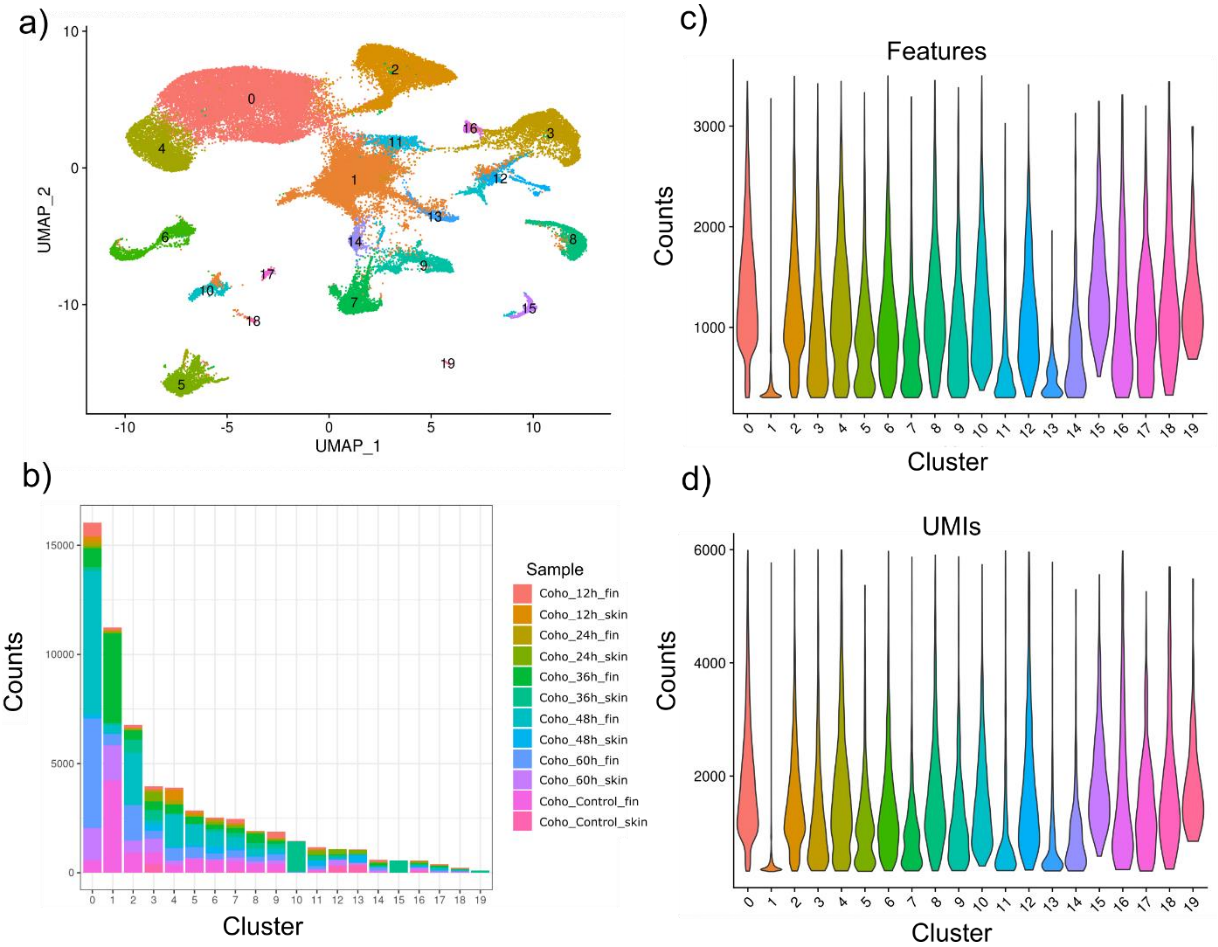

Fig.S43 Cell clusters after initial integration of coho salmon samples: a) UMAP, b) number of cells per cluster per sample, c) Violin plot of the distribution of feature counts per cluster, d) Violin plot of the distribution of UMI counts per cluster. Note that cluster 1 was removed from subsequent analysis due to low average UMI/feature counts.

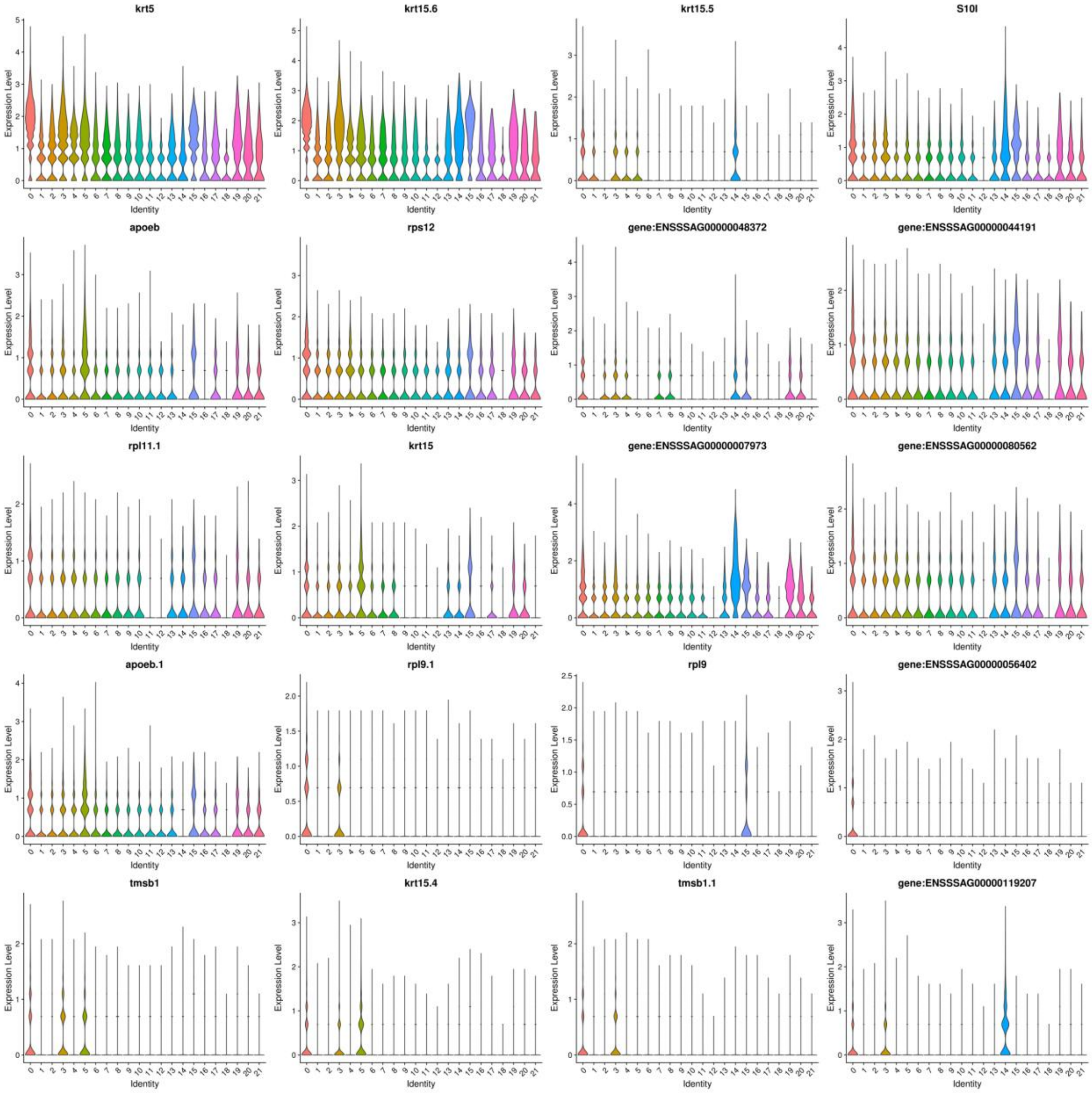

Fig.S44 Violin plots of expression levels (based on the SCT assay) for the top 20 significant (p-adjusted < 0.05) marker genes for cluster 0 (based on log-scale two-fold change in expression) after the initial integration of Atlantic salmon samples (see Fig.S42 for UMAP). Note the abundance of ribosomal proteins.

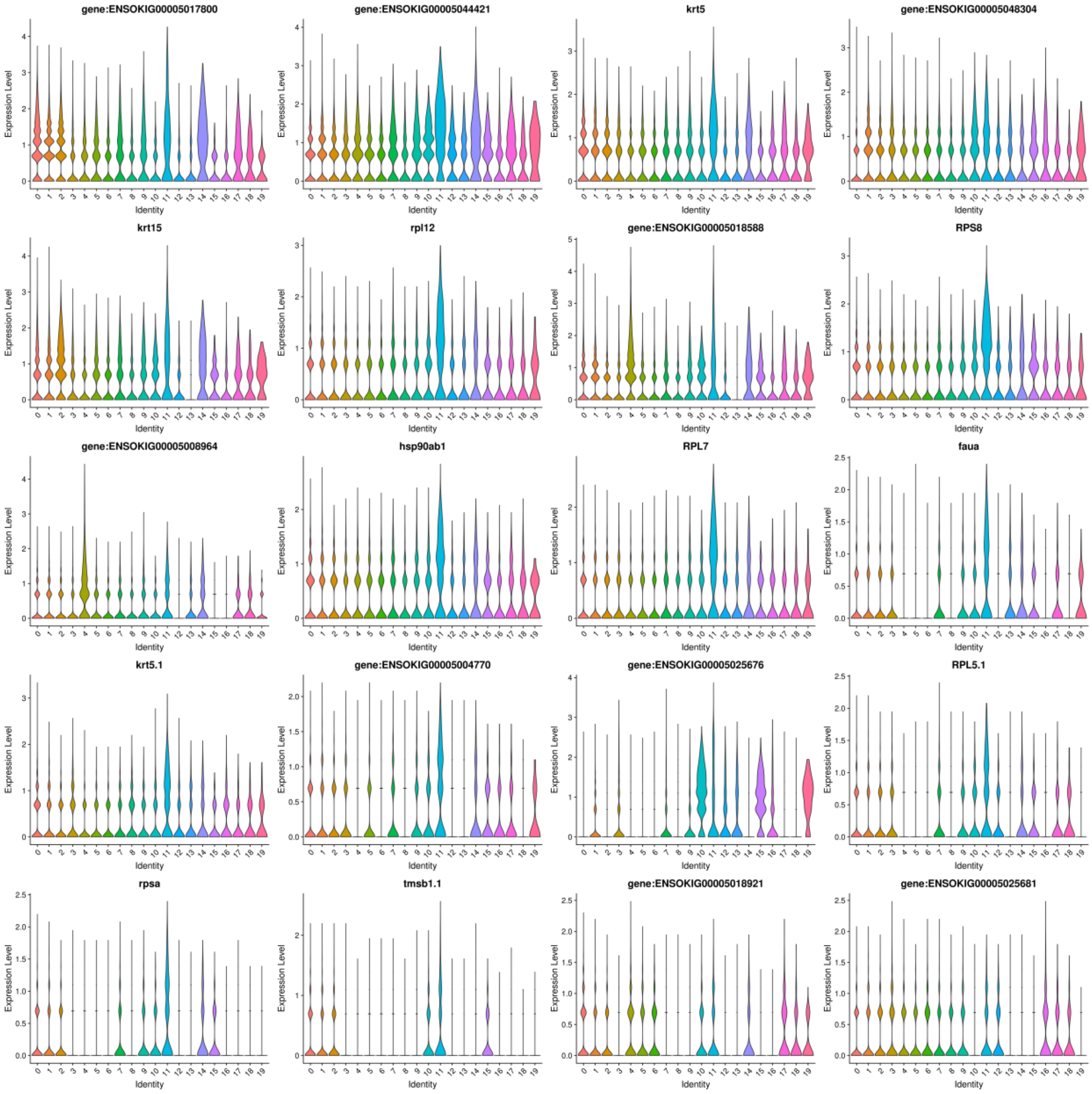

Fig.S45 Violin plots of expression levels (based on the SCT assay) for the top 20 significant (p-adjusted < 0.05) marker genes for cluster 1 (based on log-scale two-fold change in expression) after the initial integration of coho salmon samples (see Fig.S43 for UMAP). Note the abundance of ribosomal proteins.

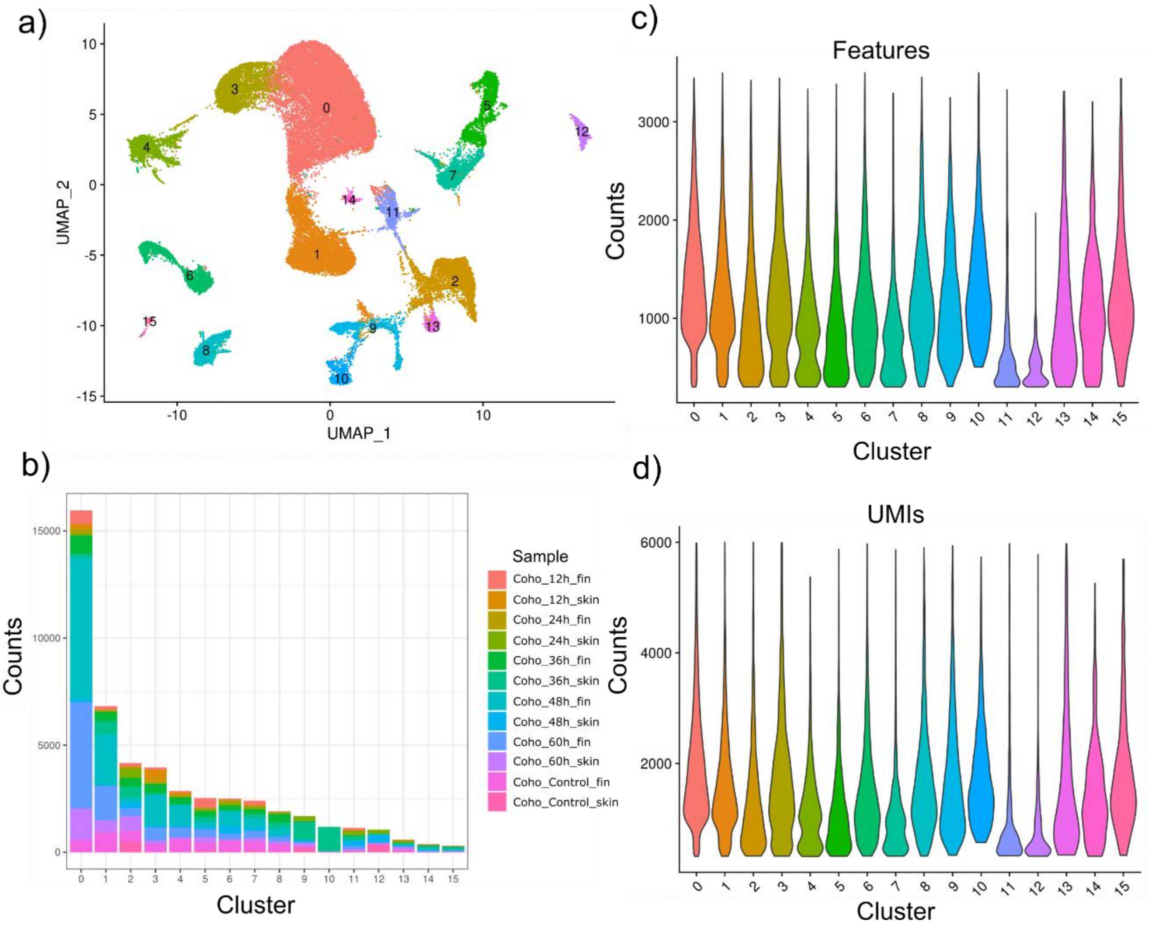

Fig.S46 Cell clusters after removing one cluster (cluster 1 from Fig.S43) and re-integrating/clustering coho salmon samples: a) UMAP, b) number of cells per cluster per sample, c) Violin plot of the distribution of feature counts per cluster, d) Violin plot of the distribution of UMI counts per cluster.

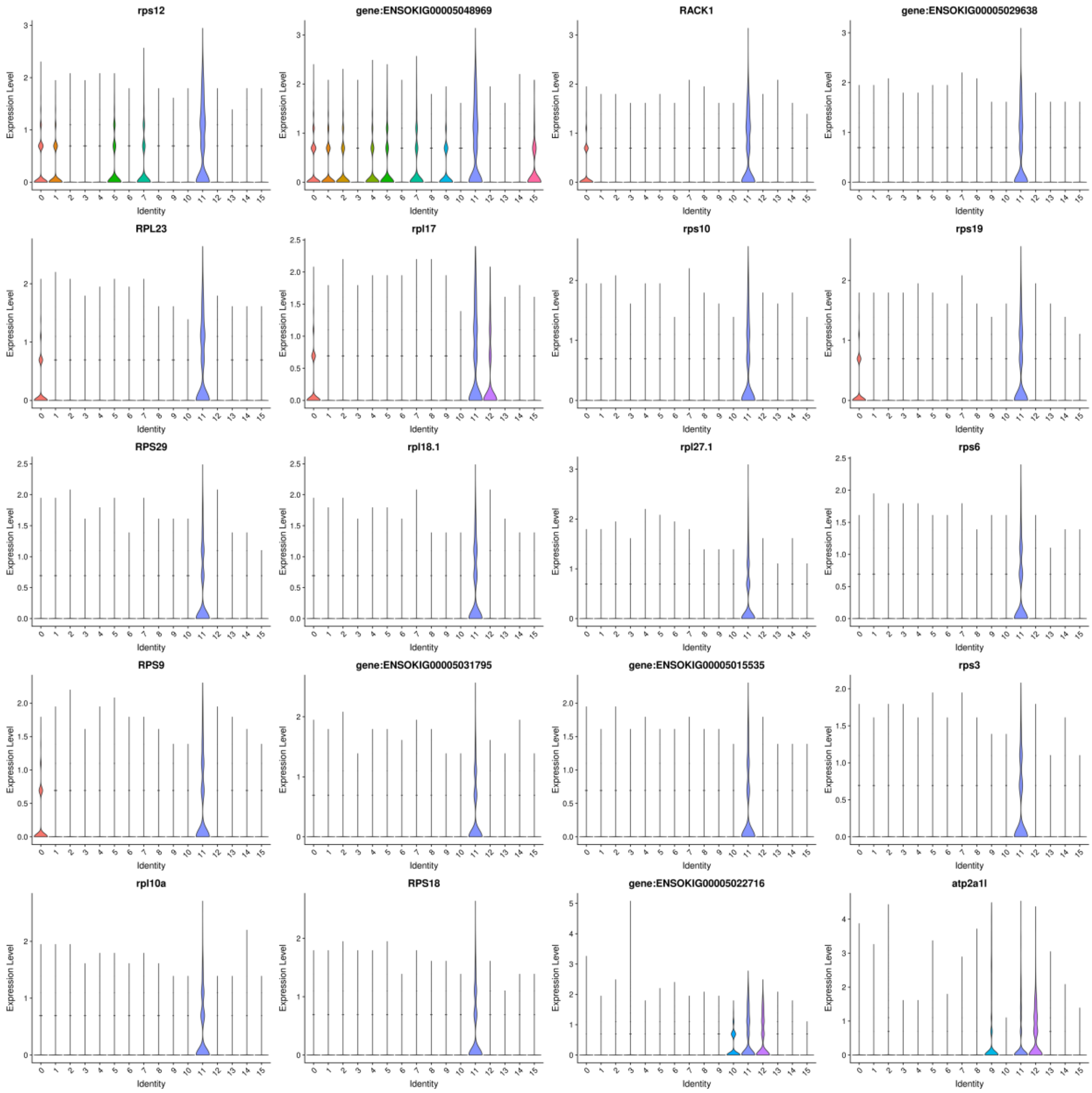

Fig.S47 Violin plots of expression levels (based on the SCT assay) for the top 20 significant (p-adjusted < 0.05) marker genes for cluster 11 (based on log-scale two-fold change in expression) after removing one cluster (cluster 1 from Fig.S43) and re-integrating/clustering coho salmon samples (see Fig.S46 for UMAP). Note the abundance of ribosomal proteins.

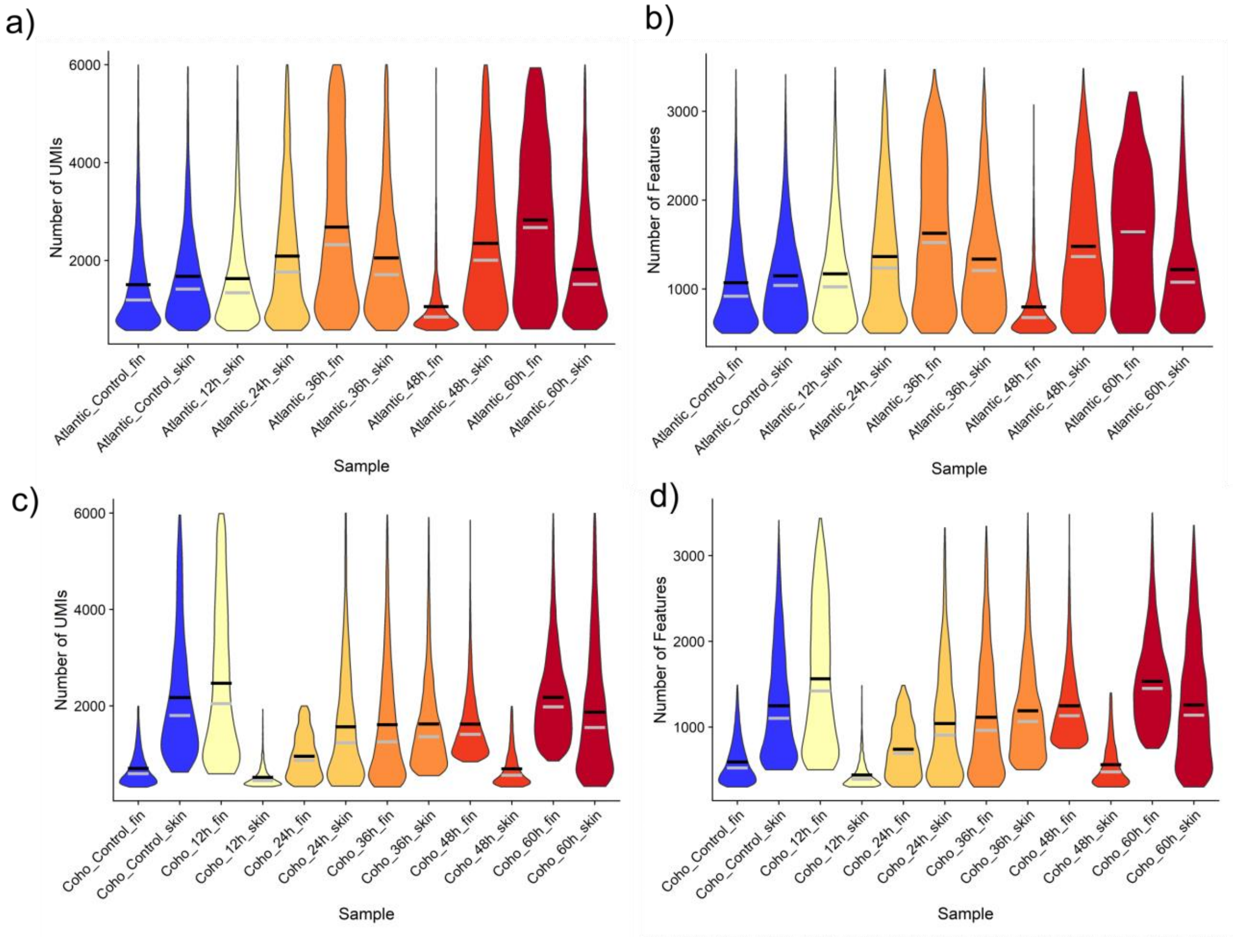

Fig.S48 Distribution of UMIs and features for Atlantic salmon (a, b) and coho salmon (c, d) samples. Horizontal black/grey bars indicate the mean/median value for each sample, respectively.

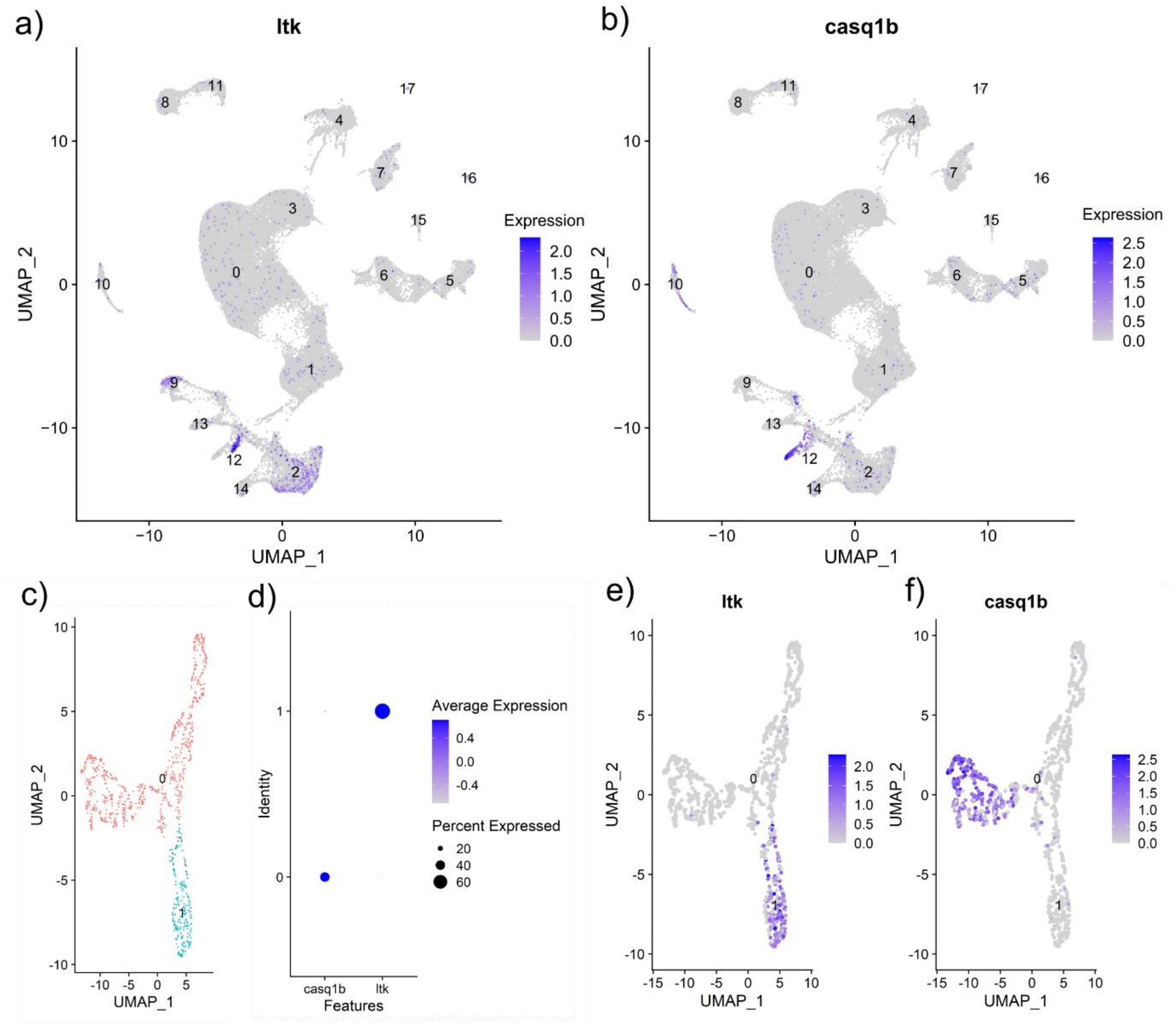

Fig.S49 Subclustering of cluster 12 within coho salmon. Feature Plots indicate expression (based on the SCT assay) of *ltk* (a) and *casq1b* (b) in different cells within cluster 12. Re-clustering those cells within cluster 12 using 3 PCs and a resolution of 0.02 revealed two clusters as visualized in a UMAP (c). One cluster expressed *casq1b*, the other expressed *ltk* as shown in a Dot Plot (d) and Feature Plots (e, f).

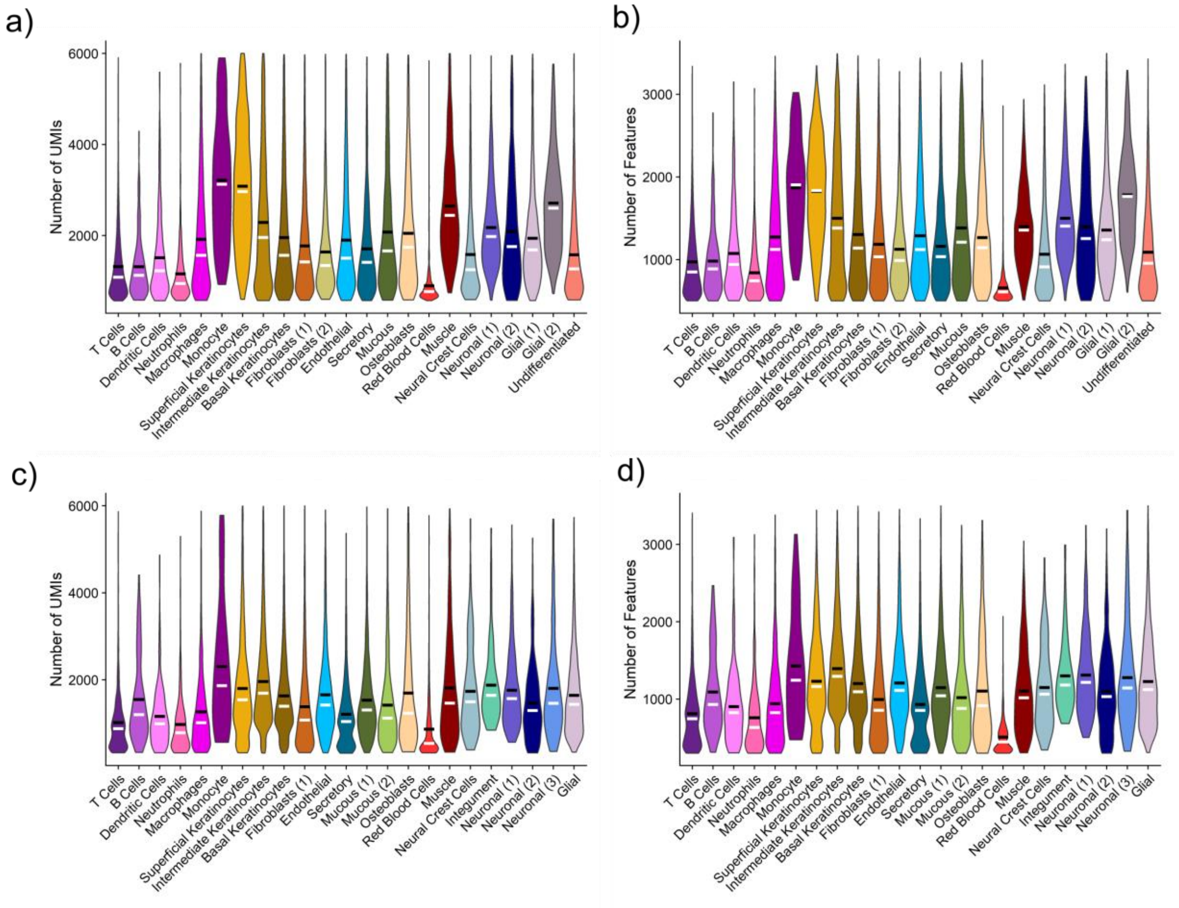

Fig.S50 Distribution of UMIs and features for each cluster identified in Atlantic salmon (a, b) and coho salmon (c, d). Horizontal black/white bars indicate the mean/median value for each cell type, respectively.

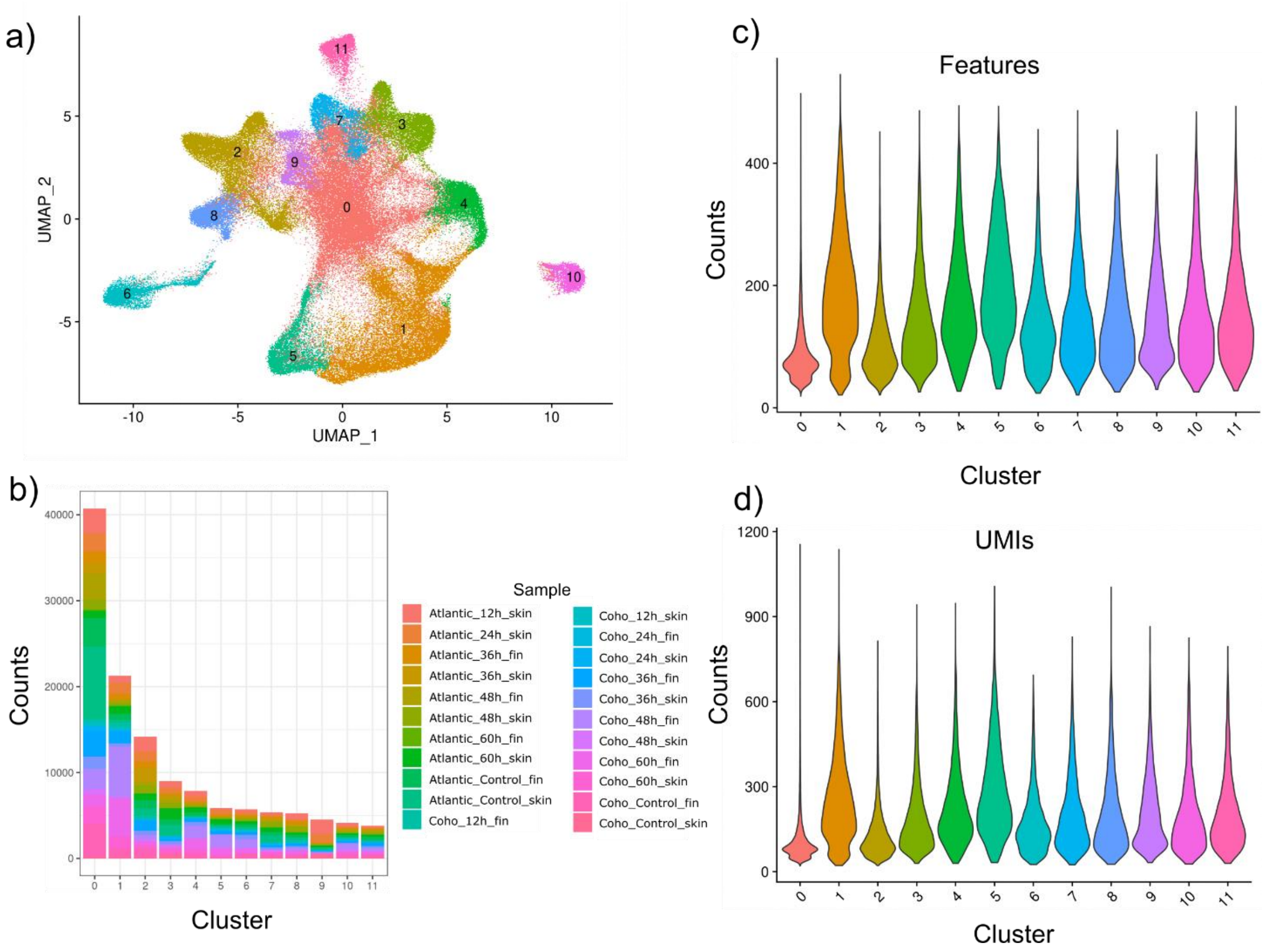

Fig.S51 Cell clusters after initial integration of Atlantic and coho salmon samples: a) UMAP, b) number of cells per cluster per sample, c) Violin plot of the distribution of feature counts per cluster, d) Violin plot of the distribution of UMI counts per cluster. Note that cluster 0 was removed from subsequent analysis.

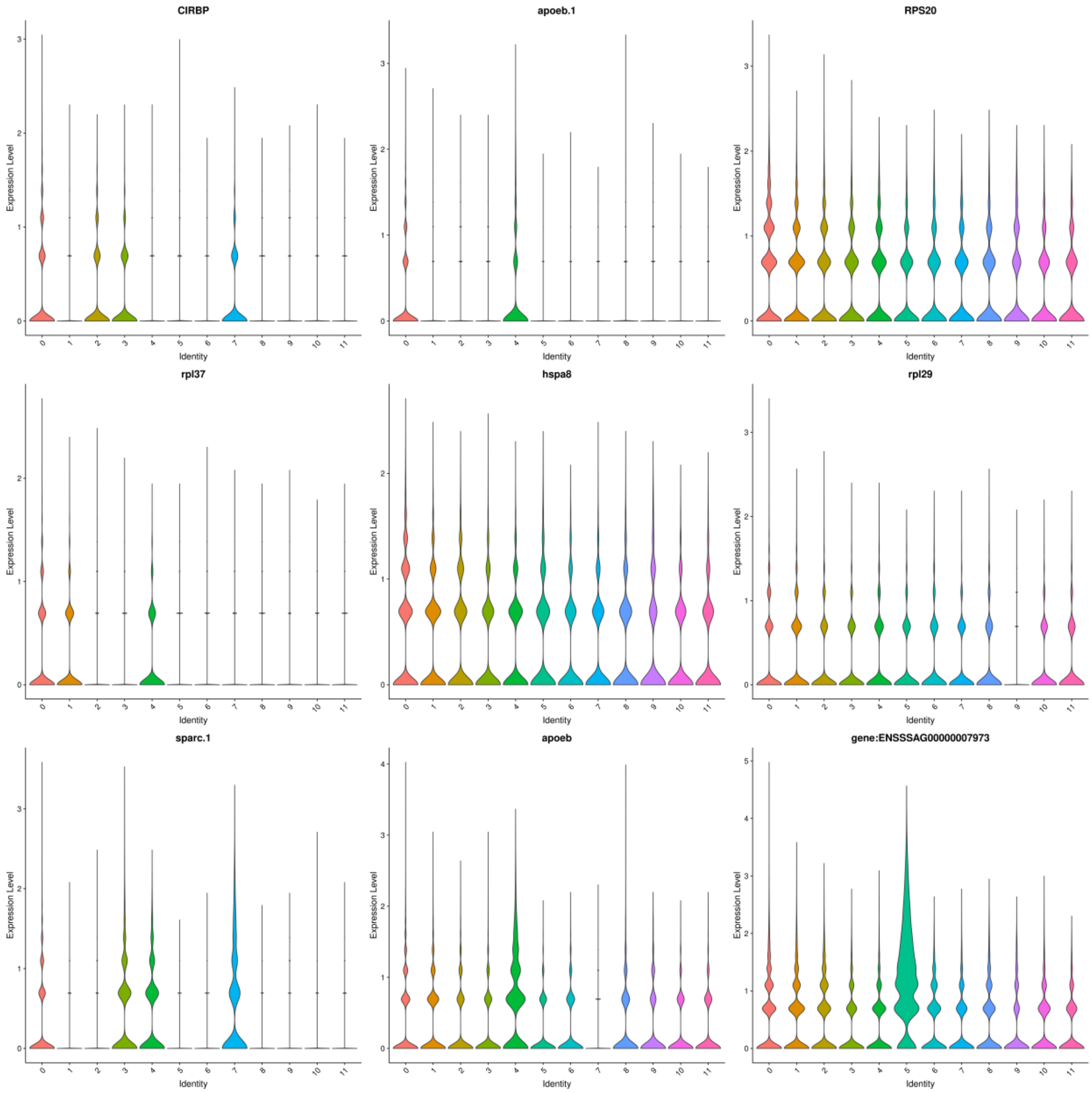

Fig.S52 Violin plots of expression levels (based on the SCT assay) for the 9 significant (adjusted p-value < 0.05) marker genes for cluster 0 (based on log-scale two-fold change in expression) of the dataset integrating both Atlantic salmon and coho salmon samples (for UMAP see Fig.S51).

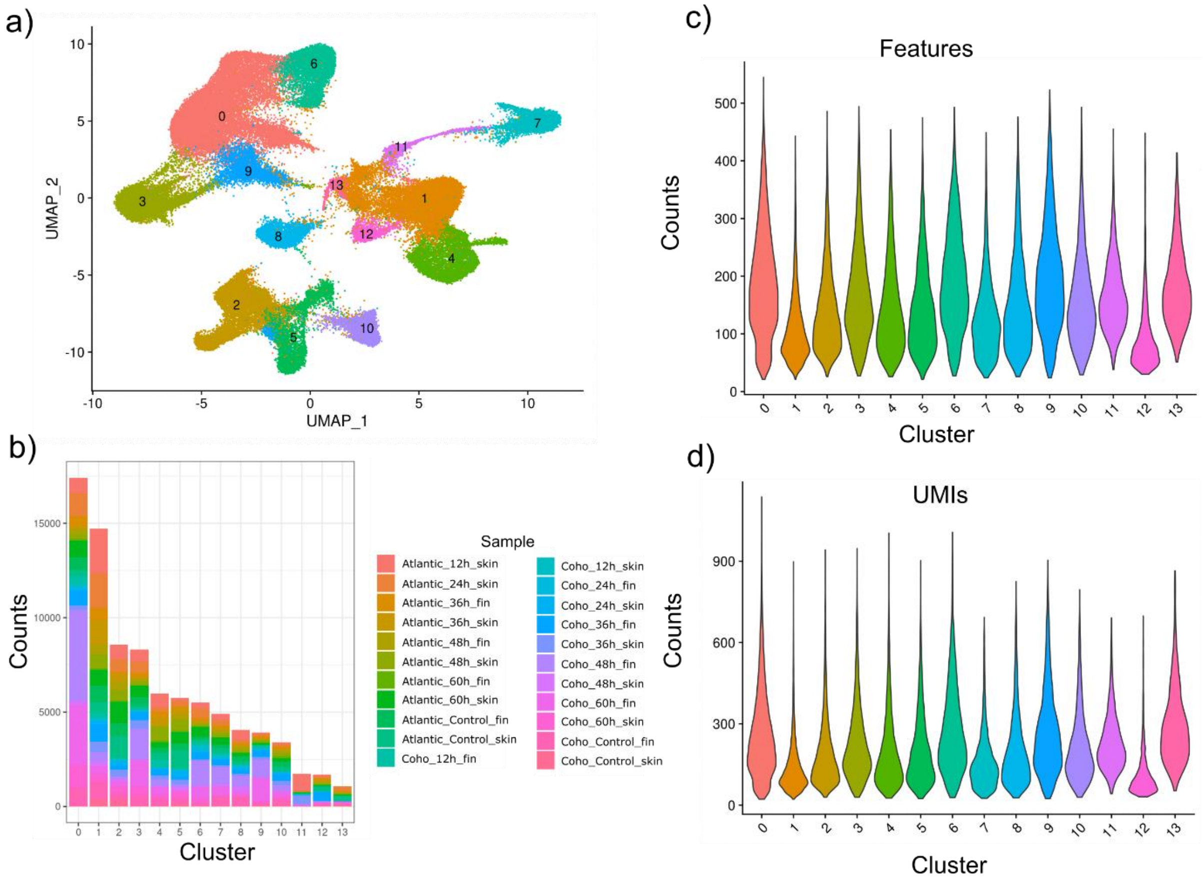

Fig.S53 Cell clusters after removing one cluster (cluster 0 from Fig.S51) and re-integrating/clustering: a) UMAP, b) number of cells per cluster per sample, c) Violin plot of the distribution of feature counts per cluster, d) Violin plot of the distribution of UMI counts per cluster. Note that cluster 1 was removed from subsequent analysis.

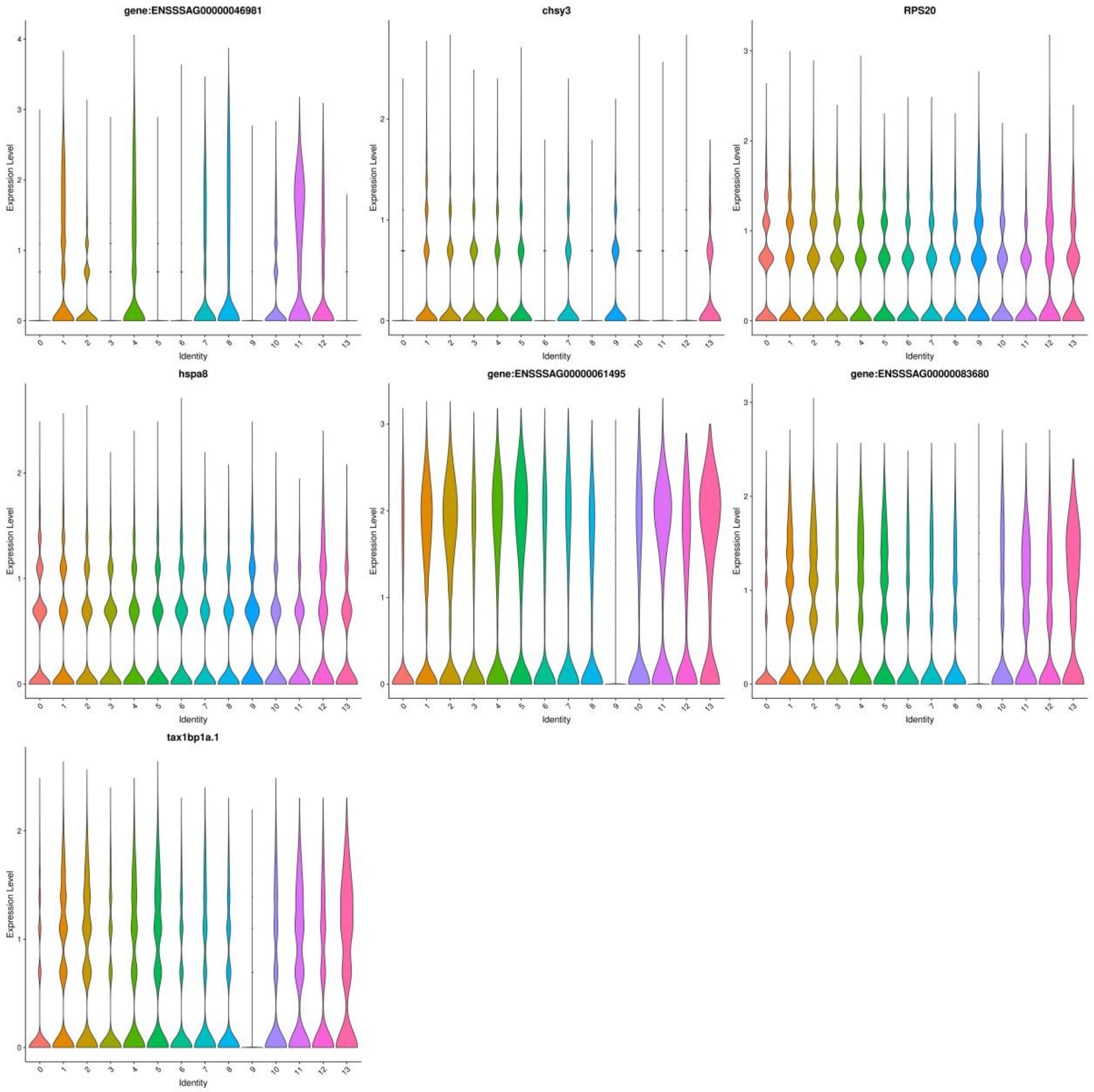

Fig.S54 Violin plots of expression levels (based on the SCT assay) for the 7 significant (adjusted p-value < 0.05) marker genes for cluster 1 (based on log-scale two-fold change in expression) of the dataset integrating both Atlantic and Coho samples (for UMAP see Fig.S53).

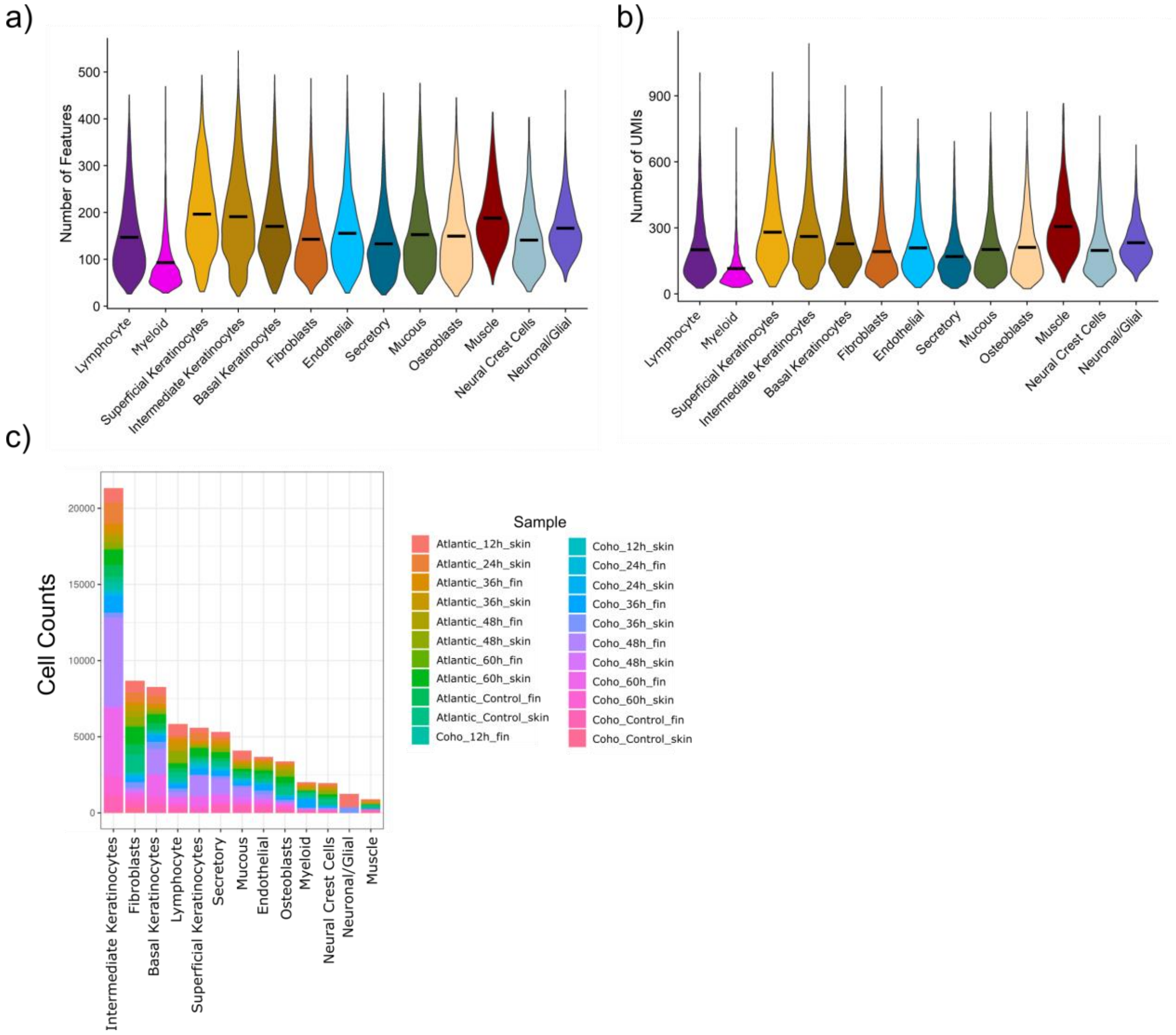

Fig.S55 Cell clusters after removing one cluster (cluster 1 from Fig.S53) and re-integrating/clustering: a) Violin plot of the distribution of feature counts per cluster, b) Violin plot of the distribution of UMI counts per cluster, c) number of cells per cluster per sample. Horizontal black bar indicates the mean value for each sample in a and b.

Table S1 Summary statistics for STAR outputs for each Atlantic salmon sample. Please note that raw files were used for downstream analysis (which includes all barcodes, not just those estimated to be viable cells by STAR). Therefore, the statistics relating to STAR-assigned cells are not necessarily relevant, but are provided for the reader's interest.

**Stats**	**Atlantic_ Control_ skin**	**Atlantic_ Control_ fin**	**Atlantic_ 12h_ skin**	**Atlantic_ 12h_ fin**	**Atlantic_ 24h_ skin**	**Atlantic_ 24h_ fin**	**Atlantic_ 36h_ skin**	**Atlantic_ 36h_ fin**	**Atlantic_ 48h_ skin**	**Atlantic_ 48h_ fin**	**Atlantic_ 60h_ skin**	**Atlantic_ 60h_ fin**
**Number of Reads**	321955411	295551337	287042718	353678800	282562462	110112712	315629173	393313790	264259178	310071740	263338701	381111089
**Reads With Valid Barcodes**	0.936286	0.93991	0.918735	0.966361	0.950211	0.971276	0.968462	0.955684	0.933665	0.938435	0.943417	0.961351
**Sequencing Saturation**	0.43391	0.481264	0.461568	0.995379	0.525175	0.899236	0.599346	0.697027	0.561006	0.54836	0.555422	0.759148
**Q30 Bases in CB+UMI**	0.965611	0.965352	0.977091	0.941605	0.969034	0.961646	0.976242	0.962104	0.976833	0.973577	0.97554	0.976305
**Q30 Bases in RNA read**	0.927049	0.925024	0.938372	0.839489	0.932749	0.937356	0.945648	0.921885	0.935666	0.928847	0.938162	0.941215
**Reads Mapped to Genome: Unique+Multiple**	0.456254	0.472516	0.469762	0.599451	0.598245	0.775026	0.47207	0.641069	0.564607	0.463942	0.501395	0.277742
**Reads Mapped to Genome: Unique**	0.352346	0.364219	0.360404	0.522241	0.483287	0.663975	0.368411	0.513612	0.459498	0.336774	0.391177	0.232388
**Reads Mapped to GeneFull: Unique+Multiple GeneFull**	0.349119	0.362229	0.344158	0.471235	0.449156	0.609792	0.360462	0.457549	0.419502	0.357637	0.386086	0.204369
**Reads Mapped to GeneFull: Unique GeneFull**	0.273161	0.286393	0.271067	0.414637	0.372626	0.532902	0.289934	0.378298	0.350904	0.262615	0.309893	0.173523
**Estimated Number of Cells**	9866	4708	8982	2081	6245	426	2795	3068	3313	6464	5732	878
**Unique Reads in Cells Mapped to GeneFull**	32658055	30082325	36733011	37934797	46115933	7627481	31262241	67110528	40149817	22545541	36527251	27350059
**Fraction of Unique Reads in Cells**	0.371344	0.355399	0.4721	0.258679	0.43799	0.129986	0.34162	0.451043	0.432977	0.276872	0.447601	0.41357
**Mean Reads per Cell**	3310	6389	4089	18229	7384	17904	11185	21874	12118	3487	6372	31150
**Median Reads per Cell**	2554	4893	3320	10845	6016	6720	8064	18625	9398	2513	5026	21397
**UMIs in Cells**	17226103	13155840	18750612	217030	20834285	775112	11320805	18310523	16177823	9416816	14685030	6378850
**Mean UMI per Cell**	1746	2794	2087	104	3336	1819	4050	5968	4883	1456	2561	7265
**Median UMI per Cell**	1373	2182	1701	77	2732	844	2908	5137	3803	1084	2052	5129
**Mean GeneFull per Cell**	1124	1543	1394	83	1906	1044	2135	2898	2480	898	1535	3021
**Median GeneFull per Cell**	958	1327	1211	68	1692	653	1786	2728	2155	752	1328	2577
**Total GeneFull Detected**	45581	43079	46794	21227	45651	30251	44217	44983	44453	42483	44293	40529

Table S2 Summary statistics for STAR outputs for each coho salmon sample. Please note that raw files were used for downstream analysis (which includes all barcodes, not just those estimated to be viable cells by STAR). Therefore, the statistics relating to STAR-assigned cells are not necessarily relevant, but are provided for the reader's interest.

Stats	Coho_ Control_ skin	Coho_ Control_ fin	Coho_ 12h_ skin	Coho_ 12h_ fin	Coho_ 24h_ skin	Coho_ 24h_ fin	Coho_ 36h_ skin	Coho_ 36h_ fin	Coho_ 48h_ skin	Coho_ 48h_ fin	Coho_ 60h_ skin	Coho_ 60h_ fin
**Number of Reads**	272572603	276464716	278541953	275427587	299891579	244684146	335320286	381640818	259657264	311390827	260830113	420924570
**Reads With Valid Barcodes**	0.941577	0.920432	0.860409	0.94946	0.953507	0.893923	0.941946	0.923695	0.942316	0.897022	0.961607	0.959087
**Sequencing Saturation**	0.776908	0.463412	0.820663	0.606011	0.793786	0.625526	0.520646	0.681679	0.64073	0.353565	0.581034	0.574321
**Q30 Bases in CB+UMI**	0.974244	0.973359	0.972798	0.975214	0.974505	0.971095	0.973501	0.962014	0.971916	0.975593	0.972391	0.962382
**Q30 Bases in RNA read**	0.940897	0.925045	0.921822	0.936799	0.935628	0.92402	0.925214	0.911675	0.927226	0.929605	0.918603	0.910774
**Reads Mapped to Genome: Unique+Multiple**	0.535037	0.318965	0.234634	0.516494	0.486003	0.243276	0.374254	0.379116	0.371447	0.46242	0.414026	0.565078
**Reads Mapped to Genome: Unique**	0.473984	0.282777	0.202723	0.457842	0.428401	0.209409	0.331012	0.335661	0.31976	0.414474	0.367817	0.503185
**Reads Mapped to GeneFull: Unique+Multiple GeneFull**	0.383204	0.198197	0.128836	0.32536	0.296861	0.141313	0.229477	0.223146	0.216348	0.267375	0.26304	0.338743
**Reads Mapped to GeneFull: Unique GeneFull**	0.33425	0.17752	0.111288	0.289536	0.261617	0.123318	0.205097	0.19756	0.18971	0.241075	0.236002	0.30292
**Estimated Number of Cells**	1851	9314	8364	2424	1351	1997	5178	2937	2676	27719	4562	12757
**Unique Reads in Cells Mapped to GeneFull**	28060242	17302048	12889879	32016959	24601862	7203141	25099571	24465648	11997431	52586806	26501624	63480222
**Fraction of Unique Reads in Cells**	0.307991	0.352541	0.415825	0.401486	0.313572	0.23872	0.364961	0.324492	0.243555	0.700518	0.430525	0.497859
**Mean Reads per Cell**	15159	1857	1541	13208	18210	3606	4847	8330	4483	1897	5809	4976
**Median Reads per Cell**	10737	1378	1130	10670	13369	2832	3809	6559	3223	1503	4709	4358
**UMIs in Cells**	5979997	8830040	1977143	11436790	4554764	2510949	11232707	6661804	3817768	33617687	10393018	26132182
**Mean UMI per Cell**	3230	948	236	4718	3371	1257	2169	2268	1426	1212	2278	2048
**Median UMI per Cell**	2291	717	181	3877	2492	992	1725	1842	1026	972	1847	1803
**Mean GeneFull per Cell**	1465	667	205	2401	1780	819	1376	1441	805	940	1413	1404
**Median GeneFull per Cell**	1248	554	162	2178	1550	689	1209	1274	663	797	1247	1312
**Total GeneFull Detected**	35062	34812	35919	35011	34321	30685	37853	34492	32828	38580	36130	38153

Table S3 Number of nuclei detected per sample after several filtering stages: 1) initial filtering removing nuclei with fewer than 200 features, 2) removing nuclei where mtDNA features account for 10% or more of all UMIs, 3) removing nuclei that exceeded UMI/feature upper and lower bounds, 4) removing doublet cells identified with DoubletFinder, 5) removing cells in poor quality clusters. Note that sample Atlantic_12h_fin was removed from subsequent analysis after the initial filtering due to a low cell count (highlighted). Sample Atlantic_24h_fin was removed from subsequent analysis after removing nuclei based on mtDNA features and upper and lower UMI/feature counts due to a low cell count (highlighted). Note that because only 1:1 orthologs were used for SCTransformation prior to running DoubletFinder for the species-integrated dataset, cell counts differ for stage 4 (after removing doublet cells identified with DoubletFinder) and 5 (removing cells in poor quality clusters) between the species-integrated and species-specific datasets and are noted in separate columns. However, filtering for stages 1-3 were identical for the species-specific and species-integrated datasets.

	**Cell Count**						
	**All Datasets**			**Species-Specific Datasets**		**Species-Integrated Dataset**	
**Sample ID**	**1) Nuclei with > 200 features**	**2) Nuclei with < 10% of UMIs as mtDNA features**	**3) Nuclei passing upper and lower UMI/feature thresholds**	**4) Nuclei after removing doublets**	**5) Nuclei after removing poor-quality clusters**	**4) Nuclei after removing doublets**	**5) Nuclei after removing poor-quality clusters**
**Atlantic_Control_fin**	61866	55206	7896	7647	4555	7683	3531
**Atlantic_Control_skin**	66543	36325	13648	13217	7290	13242	4263
**Atlantic_12h_fin**	60						
**Atlantic_12h_skin**	60811	54419	11139	10730	9962	10765	5515
**Atlantic_24h_fin**	434	431	338				
**Atlantic_24h_skin**	64345	51918	8804	8488	7398	8514	4544
**Atlantic_36h_fin**	66375	66193	4136	3995	2981	4001	2063
**Atlantic_36h_skin**	58441	57179	4817	4648	3820	4656	2076
**Atlantic_48h_fin**	69318	47909	5547	5383	2924	5484	1916
**Atlantic_48h_skin**	60731	53298	5140	4961	4443	4959	3084
**Atlantic_60h_fin**	2190	2106	831	804	671	807	572
**Atlantic_60h_skin**	57553	53337	6928	6683	6284	6691	4987
**Coho_Control_fin**	57310	38435	9344	9078	4811	9145	4348
**Coho_Control_skin**	5427	1961	1444	1394	1341	1394	840
**Coho_12h_fin**	54254	45595	2269	2192	1969	2193	1592
**Coho_12h_skin**	2969	2920	1128	1096	1020	118	557
**Coho_24h_fin**	3040	1481	844	819	799	826	741
**Coho_24h_skin**	5642	4875	1920	1854	1579	1859	1074
**Coho_36h_fin**	46548	44540	8025	7803	3642	7820	3987
**Coho_36h_skin**	59963	40633	4901	4727	4438	4731	2682
**Coho_48h_fin**	57552	53458	14364	13921	13524	13967	11153
**Coho_48h_skin**	17454	8991	2097	2025	1755	2042	1150
**Coho_60h_fin**	62865	59425	10397	10051	9380	10122	8246
**Coho_60h_skin**	45109	20449	5952	5751	4083	5779	3418

Table S4 Number of cells per cell type in each Atlantic salmon sample. Note that cell types with fewer than 50 cells in all control samples and time points with fewer than 50 cells were excluded from differential expression analyses. These excluded cell types and times are highlighted in yellow.

	Samples									
**Cell Type**	**Atlantic_60h_fin**	**Atlantic_60h_skin**	**Atlantic_48h_fin**	**Atlantic_48h_skin**	**Atlantic_36h_fin**	**Atlantic_36h_skin**	**Atlantic_24h_skin**	**Atlantic_12h_skin**	**Atlantic_Control_fin**	**Atlantic_Control_skin**
Monocyte	1	2	0	80	1	8	4	3	2	4
Neuronal (2)	2	33	14	11	41	14	40	118	37	16
B Cells	0	27	1	26	3	22	66	163	3	34
Neutrophils	28	14	7	123	87	180	70	50	43	17
Muscle	0	64	0	53	0	154	167	0	0	312
Dendritic Cells	9	94	37	53	26	97	86	191	72	95
Glial (2)	0	0	0	0	0	0	1	771	0	0
Undifferentiated	16	52	114	138	32	51	80	97	44	274
Fibroblasts (2)	0	0	3	200	1	107	3	1	6	927
Neural Crest Cells	0	344	0	178	0	139	170	47	2	506
Neuronal (1)	0	0	0	0	0	0	2	1510	0	0
Red Blood Cells	2	266	0	127	8	114	785	33	30	219
Superficial Keratinocytes	21	372	67	66	131	110	401	223	112	154
Glial (1)	8	51	8	30	25	6	89	1475	91	15
Endothelial	115	201	115	155	152	96	162	166	438	318
Osteoblasts	12	306	117	254	37	168	229	29	198	602
Mucous	19	165	161	124	183	85	274	458	332	212
Secretory	16	370	180	216	174	114	278	224	224	357
Macrophages	54	164	33	575	210	211	168	340	163	401
Basal Keratinocytes	52	680	357	195	235	100	714	926	534	191
Intermediate Keratinocytes	145	1075	1054	237	740	179	1618	795	806	256
Fibroblasts (1)	99	1342	498	912	215	420	794	746	811	1655
T Cells	72	662	158	690	680	1445	1197	1596	607	725

Table S5 Number of cells per cell type in each coho salmon sample. Note that cell types with fewer than 50 cells in all control samples and time points with fewer than 50 cells were excluded from differential expression analyses. These excluded cell types and times are highlighted in yellow.

	Samples											
**Cell Type**	**Coho_60h_fin**	**Coho_60h_skin**	**Coho_48h_fin**	**Coho_48h_skin**	**Coho_36h_fin**	**Coho_36h_skin**	**Coho_24h_fin**	**Coho_24h_skin**	**Coho_12h_fin**	**Coho_12h_skin**	**Coho_Control_fin**	**Coho_Control_skin**
B Cells	0	4	0	13	0	9	1	5	0	0	1	30
Monocyte	0	30	1	2	0	34	0	0	3	0	0	0
Integument	0	6	0	0	0	87	0	0	0	0	0	0
Neural Crest Cells	2	4	0	30	2	5	0	117	5	0	1	44
Neuronal (3)	26	9	61	13	22	9	3	5	24	11	31	0
Dendritic Cells	52	30	51	7	18	45	2	18	43	6	50	5
Neuronal (2)	49	18	118	19	64	27	7	10	56	3	17	1
Osteoblasts	20	132	8	65	25	83	9	74	15	37	50	53
Neutrophils	134	80	39	11	120	27	2	51	116	1	34	6
Neuronal (1)	1	6	1	0	0	654	0	0	0	0	0	0
Muscle	18	181	9	61	21	81	0	108	1	1	48	191
Mucous (2)	74	45	136	345	26	91	43	115	23	2	116	16
Red Blood Cells	0	94	0	376	0	32	1	197	4	0	1	364
Glial	3	29	7	0	9	1254	0	17	3	0	15	21
Macrophages	217	132	185	26	103	117	9	67	269	14	282	19
Mucous (1)	187	17	544	0	177	5	65	5	54	15	418	1
Endothelial	234	122	330	44	399	265	34	70	26	1	362	19
T Cells	321	191	455	127	225	190	25	115	209	63	457	53
Secretory	484	63	945	91	328	43	61	23	64	132	602	18
Superficial Keratinocytes	585	204	1358	59	413	57	195	21	105	418	222	5
Fibroblasts (1)	356	655	191	276	405	502	83	428	156	22	518	466
Basal Keratinocytes	1620	589	2422	15	424	631	59	32	146	27	904	11
Intermediate Keratinocytes	4997	1442	6663	175	861	190	200	101	647	267	682	18

Table S6 Number of cells per cluster and sampling time point after reclustering only the immune cells within the Atlantic salmon samples.

	Sample Time Point					
	**12h**	**24h**	**36h**	**48h**	**60h**	**Control**
T Cells (1)	909	528	976	410	371	491
T Cells (2)	499	432	467	206	113	486
Macrophages (1)	121	30	168	429	97	268
Macrophages (2)	183	89	181	110	111	155
T Cells (3)	57	61	367	57	99	133
Dendritic Cells	191	86	123	90	103	167
T Cells (4)	92	121	157	90	108	153
Neutrophils	50	70	267	130	42	60
T Cells (5)	39	55	158	85	43	69
Macrophages (3)	36	49	72	69	10	141
B Cells	163	66	25	27	27	37
Monocyte	3	4	9	80	3	6

Table S7 Number of cells per cluster after reclustering only the immune cells within the coho salmon samples.

	Sample Time Point					
**Cluster**	**12h**	**24h**	**36h**	**48h**	**60h**	**Control**
T Cells (1)	155	101	213	271	239	236
Macrophages (1)	200	17	118	181	188	61
Macrophages (2)	83	59	102	30	161	240
Neutrophils	117	53	147	50	214	40
T Cells (2)	36	31	124	30	173	209
Dendritic Cells	49	20	63	58	82	55
T Cells (3)	1	0	7	229	22	2
T Cells (4)	17	5	41	36	61	17
T Cells (5)	63	3	30	16	17	46
Monocyte	3	0	34	3	30	0
B Cells	0	6	9	13	4	31

Table S8 Genes used for cell cycle scoring for Atlantic salmon.

Human_Symbol	Ensembl_Symbol	Ensembl_ID	Cycle
MCM5	mcm5	ENSSSAG00000040979	S.gene
MCM5	mcm5.1	ENSSSAG00000005359	S.gene
PCNA	pcna	ENSSSAG00000009453	S.gene
TYMS	tyms	ENSSSAG00000073844	S.gene
TYMS	TYMS	ENSSSAG00000070708	S.gene
MCM2	mcm2	ENSSSAG00000042717	S.gene
MCM4	mcm4	ENSSSAG00000081704	S.gene
MCM4	mcm4.1	ENSSSAG00000070777	S.gene
GINS2	psf2	ENSSSAG00000117735	S.gene
CDCA7	cdca7a	ENSSSAG00000071616	S.gene
DTL	DTL	ENSSSAG00000074405	S.gene
DTL	DTL.1	ENSSSAG00000058407	S.gene
UHRF1	uhrf1	ENSSSAG00000072643	S.gene
UHRF1	uhrf1.1	ENSSSAG00000046494	S.gene
RFC2	rfc2	ENSSSAG00000066661	S.gene
NASP	nasp	ENSSSAG00000049742	S.gene
RAD51AP1	rad51ap1	ENSSSAG00000046281	S.gene
RAD51AP1	rad51ap1.1	ENSSSAG00000002591	S.gene
GMNN	gene:ENSSSAG00000052775	ENSSSAG00000052775	S.gene
WDR76	gene:ENSSSAG00000052378	ENSSSAG00000052378	S.gene
SLBP	slbp	ENSSSAG00000063884	S.gene
CCNE2	ccne2	ENSSSAG00000059043	S.gene
CCNE2	ccne2.1	ENSSSAG00000057367	S.gene
UBR7	ubr7	ENSSSAG00000040956	S.gene
POLD3	gene:ENSSSAG00000005314	ENSSSAG00000005314	S.gene
MSH2	msh2	ENSSSAG00000074238	S.gene
ATAD2	ATAD2	ENSSSAG00000059621	S.gene
ATAD2	ATAD2.1	ENSSSAG00000003726	S.gene
RAD51	rad51	ENSSSAG00000045336	S.gene
RRM2	RRM2	ENSSSAG00000083858	S.gene
CDC45	cdc45	ENSSSAG00000047205	S.gene
CDC45	cdc45.1	ENSSSAG00000047454	S.gene
CDC6	cdc6	ENSSSAG00000041769	S.gene
EXO1	exo1	ENSSSAG00000103371	S.gene
TIPIN	tipin	ENSSSAG00000082602	S.gene
TIPIN	tipin.1	ENSSSAG00000043439	S.gene
DSCC1	dscc1	ENSSSAG00000088925	S.gene
DSCC1	dscc1.1	ENSSSAG00000104335	S.gene
DSCC1	DSCC1	ENSSSAG00000098087	S.gene
BLM	blm	ENSSSAG00000078058	S.gene
CASP8AP2	casp8ap2	ENSSSAG00000045001	S.gene
CASP8AP2	casp8ap2.1	ENSSSAG00000101584	S.gene
USP1	usp1	ENSSSAG00000044868	S.gene
CLSPN	CLSPN	ENSSSAG00000054174	S.gene

CLSPN	CLSPN.1	ENSSSAG00000078544	S.gene
POLA1	pola1	ENSSSAG00000078390	S.gene
CHAF1B	chaf1b	ENSSSAG00000042565	S.gene
BRIP1	brip1	ENSSSAG00000051666	S.gene
E2F8	e2f8	ENSSSAG00000065538	S.gene
E2F8	e2f8.1	ENSSSAG00000073983	S.gene
HMGB2	hmgb2a	ENSSSAG00000054548	G2M.gene
HMGB2	hmgb2a.1	ENSSSAG00000109524	G2M.gene
CDK1	cdk1	ENSSSAG00000006872	G2M.gene
NUSAP1	nusap1	ENSSSAG00000000078	G2M.gene
NUSAP1	nusap1.1	ENSSSAG00000063363	G2M.gene
BIRC5	birc5a	ENSSSAG00000001277	G2M.gene
BIRC5	birc5b	ENSSSAG00000068971	G2M.gene
BIRC5	birc5b.1	ENSSSAG00000095509	G2M.gene
TPX2	gene:ENSSSAG00000075691	NSSSAG00000075691	G2M.gene
TOP2A	gene:ENSSSAG00000066721	ENSSSAG00000066721	G2M.gene
NDC80	ndc80	ENSSSAG00000075612	G2M.gene
CKS2	cks2	ENSSSAG00000063439	G2M.gene
CKS2	CKS2	ENSSSAG00000053543	G2M.gene
NUF2	nuf2	ENSSSAG00000091386	G2M.gene
TMPO	tmpoa	ENSSSAG00000097715	G2M.gene
TMPO	lap2	ENSSSAG00000070216	G2M.gene
TMPO	tmpob	ENSSSAG00000079549	G2M.gene
TMPO	tmpob.1	ENSSSAG00000071470	G2M.gene
CENPF	gene:ENSSSAG00000002402	ENSSSAG00000002402	G2M.gene
TACC3	gene:ENSSSAG00000043775	ENSSSAG00000043775	G2M.gene
SMC4	smc4	ENSSSAG00000074267	G2M.gene
CCNB2	ccnb2	ENSSSAG00000072975	G2M.gene
CCNB2	ccnb2.1	ENSSSAG00000080343	G2M.gene
CKAP2L	ckap2l	ENSSSAG00000045445	G2M.gene
KIF11	kif11	ENSSSAG00000006907	G2M.gene
KIF11	kif11.1	ENSSSAG00000071263	G2M.gene
ANP32E	ANP32E	ENSSSAG00000068348	G2M.gene
ANP32E	ANP32E.1	ENSSSAG00000041648	G2M.gene
TUBB4B	tubb4b	ENSSSAG00000101622	G2M.gene
KIF20B	kif20bb	ENSSSAG00000079056	G2M.gene
KIF20B	kif20bb.1	ENSSSAG00000068302	G2M.gene
KIF20B	kif20ba	ENSSSAG00000078645	G2M.gene
KIF20B	kif20ba.1	ENSSSAG00000054281	G2M.gene
CDCA3	si:ch211-69g19.2	ENSSSAG00000040417	G2M.gene
CDC20	cdc20	ENSSSAG00000075816	G2M.gene
CDC20	cdc20.1	ENSSSAG00000075603	G2M.gene
TTK	ttk	ENSSSAG00000049671	G2M.gene
TTK	ttk.1	ENSSSAG00000009759	G2M.gene
CDC25C	CDC25C	ENSSSAG00000006736	G2M.gene
KIF2C	kif2c	ENSSSAG00000053000	G2M.gene

KIF2C	kif2c.1	ENSSSAG00000080527	G2M.gene
RANGAP1	RANGAP1	ENSSSAG00000002274	G2M.gene
RANGAP1	RANGAP1.1	ENSSSAG00000067438	G2M.gene
RANGAP1	rangap1b	ENSSSAG00000002137	G2M.gene
DLGAP5	dlgap5	ENSSSAG00000000679	G2M.gene
DLGAP5	dlgap5.1	ENSSSAG00000057319	G2M.gene
CDCA8	cdca8.1	ENSSSAG00000040950	G2M.gene
CDCA8	cdca8.1	ENSSSAG00000078302	G2M.gene
ECT2	ect2	ENSSSAG00000070771	G2M.gene
ECT2	ect2.1	ENSSSAG00000068867	G2M.gene
KIF23	KIF23	ENSSSAG00000120697	G2M.gene
KIF23	KIF23.1	ENSSSAG00000095510	G2M.gene
HMMR	hmmr	ENSSSAG00000041733	G2M.gene
AURKA	gene:ENSSSAG00000031237	ENSSSAG00000031237	G2M.gene
ANLN	ANLN	ENSSSAG00000073106	G2M.gene
ANLN	ANLN.1	ENSSSAG00000086325	G2M.gene
LBR	lbr	ENSSSAG00000044615	G2M.gene
LBR	lbr.1	ENSSSAG00000076862	G2M.gene
CKAP5	ckap5	ENSSSAG00000008344	G2M.gene
CTCF	ctcf	ENSSSAG00000064848	G2M.gene
CTCF	ctcf.1	ENSSSAG00000067932	G2M.gene
NEK2	nek2	ENSSSAG00000106767	G2M.gene
NEK2	nek2.1	ENSSSAG00000056109	G2M.gene
G2E3	G2E3	ENSSSAG00000064280	G2M.gene
G2E3	g2e3	ENSSSAG00000071501	G2M.gene
CBX5	cbx5	ENSSSAG00000096644	G2M.gene
CBX5	cbx5.1	ENSSSAG00000110153	G2M.gene
CENPA	gene:ENSSSAG00000078816	ENSSSAG00000078816	G2M.gene

Table S9 Genes used for cell cycle scoring for coho salmon.

Human_Symbol	Ensembl_Symbol	Ensembl_ID	Cycle
MCM5	mcm5	ENSOKIG00005014560	S.gene
MCM5	mcm5.1	ENSOKIG00005018292	S.gene
PCNA	pcna	ENSOKIG00005011301	S.gene
PCNA	PCNA.1	ENSOKIG00005018627	S.gene
PCNA	PCNA	ENSOKIG00005029428	S.gene
FEN1	FEN1	ENSOKIG00005024494	S.gene
FEN1	FEN1.1	ENSOKIG00005024547	S.gene
FEN1	FEN1.2	ENSOKIG00005024619	S.gene
MCM2	mcm2	ENSOKIG00005004073	S.gene
MCM2	MCM2	ENSOKIG00005017128	S.gene
MCM4	mcm4	ENSOKIG00005012623	S.gene
MCM4	mcm4.1	ENSOKIG00005042602	S.gene
RRM1	rrm1.1	ENSOKIG00005024782	S.gene
RRM1	rrm1	ENSOKIG00005046893	S.gene
UNG	unga	ENSOKIG00005050249	S.gene
GINS2	gins2	ENSOKIG00005005401	S.gene
MCM6	MCM6	ENSOKIG00005040687	S.gene
CDCA7	cdca7a	ENSOKIG00005013961	S.gene
DTL	DTL	ENSOKIG00005026623	S.gene
DTL	dtl	ENSOKIG00005013017	S.gene
PRIM1	prim1	ENSOKIG00005010009	S.gene
UHRF1	uhrf1.1	ENSOKIG00005000817	S.gene
UHRF1	uhrf1	ENSOKIG00005015452	S.gene
MLF1IP	gene:ENSOKIG00005012480	ENSOKIG00005012480	S.gene
HELLS	hells.1	ENSOKIG00005009326	S.gene
HELLS	hells	ENSOKIG00005047721	S.gene
RFC2	rfc2	ENSOKIG00005029854	S.gene
NASP	NASP	ENSOKIG00005038416	S.gene
NASP	nasp	ENSOKIG00005015929	S.gene
RAD51AP1	rad51ap1	ENSOKIG00005006131	S.gene
RAD51AP1	rad51ap1.1	ENSOKIG00005031433	S.gene
GMNN	GMNN	ENSOKIG00005004827	S.gene
GMNN	gmnn	ENSOKIG00005010054	S.gene
WDR76	wdr76	ENSOKIG00005007563	S.gene
SLBP	SLBP	ENSOKIG00005007467	S.gene
SLBP	slbp	ENSOKIG00005019708	S.gene
CCNE2	ccne2	ENSOKIG00005032974	S.gene
CCNE2	ccne2.1	ENSOKIG00005011102	S.gene
UBR7	gene:ENSOKIG00005002397	ENSOKIG00005002397	S.gene
UBR7	ubr7	ENSOKIG00005004167	S.gene
POLD3	gene:ENSOKIG00005049692	ENSOKIG00005049692	S.gene
MSH2	msh2	ENSOKIG00005029611	S.gene
ATAD2	ATAD2.1	ENSOKIG00005045862	S.gene
ATAD2	ATAD2	ENSOKIG00005004525	S.gene

ATAD2	atad2	ENSOKIG00005047340	S.gene
RAD51	rad51	ENSOKIG00005006788	S.gene
RAD51	RAD51	ENSOKIG00005029092	S.gene
RRM2	RRM2	ENSOKIG00005002061	S.gene
RRM2	RRM2.1	ENSOKIG00005036867	S.gene
CDC45	cdc45	ENSOKIG00005023527	S.gene
CDC45	cdc45.1	ENSOKIG00005020780	S.gene
CDC6	cdc6.1	ENSOKIG00005005964	S.gene
CDC6	cdc6	ENSOKIG00005004472	S.gene
EXO1	exo1	ENSOKIG00005011806	S.gene
DSCC1	dscc1.1	ENSOKIG00005047842	S.gene
DSCC1	dscc1	ENSOKIG00005048820	S.gene
BLM	blm	ENSOKIG00005006801	S.gene
CASP8AP2	casp8ap2.1	ENSOKIG00005009010	S.gene
CASP8AP2	casp8ap2	ENSOKIG00005002883	S.gene
USP1	usp1	ENSOKIG00005045064	S.gene
CLSPN	CLSPN	ENSOKIG00005015558	S.gene
CLSPN	CLSPN.1	ENSOKIG00005048701	S.gene
POLA1	pola1	ENSOKIG00005036207	S.gene
CHAF1B	chaf1b.1	ENSOKIG00005013623	S.gene
CHAF1B	chaf1b	ENSOKIG00005003095	S.gene
BRIP1	brip1	ENSOKIG00005015939	S.gene
E2F8	e2f8	ENSOKIG00005005287	S.gene
E2F8	e2f8.1	ENSOKIG00005018575	S.gene
HMGB2	hmgb2a	ENSOKIG00005013022	G2M.gene
HMGB2	hmgb2b	ENSOKIG00005037789	G2M.gene
HMGB2	hmgb2a.1	ENSOKIG00005007373	G2M.gene
CDK1	cdk1.1	ENSOKIG00005046533	G2M.gene
CDK1	cdk1	ENSOKIG00005025794	G2M.gene
NUSAP1	nusap1	ENSOKIG00005007844	G2M.gene
NUSAP1	nusap1.1	ENSOKIG00005015733	G2M.gene
UBE2C	ube2c	ENSOKIG00005000462	G2M.gene
UBE2C	ube2c.1	ENSOKIG00005000467	G2M.gene
BIRC5	birc5a	ENSOKIG00005003843	G2M.gene
BIRC5	birc5b	ENSOKIG00005039805	G2M.gene
TPX2	tpx2	ENSOKIG00005017081	G2M.gene
TOP2A	top2a	ENSOKIG00005014247	G2M.gene
NDC80	ndc80	ENSOKIG00005001262	G2M.gene
CKS2	cks2.1	ENSOKIG00005039691	G2M.gene
CKS2	CKS2	ENSOKIG00005033377	G2M.gene
CKS2	cks2	ENSOKIG00005022273	G2M.gene
NUF2	nuf2	ENSOKIG00005000381	G2M.gene
CKS1B	cks1b	ENSOKIG00005020717	G2M.gene
CKS1B	CKS1B	ENSOKIG00005027388	G2M.gene
MKI67	mki67	ENSOKIG00005013958	G2M.gene
TMPO	tmpob.1	ENSOKIG00005045692	G2M.gene

TMPO	tmpoa	ENSOKIG00005012700	G2M.gene
TMPO	tmpob	ENSOKIG00005016372	G2M.gene
CENPF	cenpf	ENSOKIG00005011884	G2M.gene
TACC3	tacc3	ENSOKIG00005001785	G2M.gene
SMC4	smc4	ENSOKIG00005048554	G2M.gene
CCNB2	ccnb2.1	ENSOKIG00005049632	G2M.gene
CCNB2	ccnb2	ENSOKIG00005050238	G2M.gene
CKAP2L	ckap2l	ENSOKIG00005002616	G2M.gene
AURKB	AURKB	ENSOKIG00005021484	G2M.gene
AURKB	aurkb	ENSOKIG00005050742	G2M.gene
BUB1	bub1	ENSOKIG00005002111	G2M.gene
KIF11	kif11.1	ENSOKIG00005046642	G2M.gene
KIF11	kif11	ENSOKIG00005023299	G2M.gene
ANP32E	ANP32E.2	ENSOKIG00005000095	G2M.gene
ANP32E	ANP32E	ENSOKIG00005013394	G2M.gene
ANP32E	ANP32E.3	ENSOKIG00005029241	G2M.gene
ANP32E	ANP32E.1	ENSOKIG00005034814	G2M.gene
GTSE1	gtse1	ENSOKIG00005022873	G2M.gene
KIF20B	kif20bb	ENSOKIG00005007330	G2M.gene
KIF20B	kif20bb.1	ENSOKIG00005022101	G2M.gene
KIF20B	kif20ba	ENSOKIG00005041578	G2M.gene
KIF20B	kif20ba.1	ENSOKIG00005039441	G2M.gene
CDCA3	si:ch211-69g19.2	ENSOKIG00005005809	G2M.gene
CDC20	cdc20	ENSOKIG00005017478	G2M.gene
TTK	ttk.1	ENSOKIG00005000616	G2M.gene
TTK	ttk.1	ENSOKIG00005000616	G2M.gene
CDC25C	ccdc25	ENSOKIG00005044242	G2M.gene
KIF2C	kif2c.1	ENSOKIG00005038836	G2M.gene
KIF2C	kif2c	ENSOKIG00005026504	G2M.gene
RANGAP1	RANGAP1	ENSOKIG00005007651	G2M.gene
RANGAP1	rangap1a	ENSOKIG00005012910	G2M.gene
RANGAP1	rangap1b	ENSOKIG00005004716	G2M.gene
NCAPD2	ncapd2	ENSOKIG00005019394	G2M.gene
DLGAP5	dlgap5	ENSOKIG00005009969	G2M.gene
CDCA2	gene:ENSOKIG00005000746	ENSOKIG00005000746	G2M.gene
CDCA8	cdca8	ENSOKIG00005014820	G2M.gene
CDCA8	cdca8.1	ENSOKIG00005048853	G2M.gene
ECT2	ect2.1	ENSOKIG00005015498	G2M.gene
ECT2	ect2	ENSOKIG00005012999	G2M.gene
KIF23	KIF23.1	ENSOKIG00005024942	G2M.gene
KIF23	KIF23	ENSOKIG00005038931	G2M.gene
HMMR	hmmr	ENSOKIG00005028767	G2M.gene
AURKA	aurka	ENSOKIG00005026781	G2M.gene
ANLN	ANLN	ENSOKIG00005016220	G2M.gene
ANLN	anln	ENSOKIG00005012931	G2M.gene
LBR	lbr	ENSOKIG00005028443	G2M.gene

LBR	lbr.1	ENSOKIG00005049627	G2M.gene
CKAP5	ckap5.1	ENSOKIG00005021670	G2M.gene
CKAP5	ckap5	ENSOKIG00005006293	G2M.gene
CTCF	CTCF	ENSOKIG00005020058	G2M.gene
CTCF	CTCF.1	ENSOKIG00005050634	G2M.gene
CTCF	ctcf.1	ENSOKIG00005001291	G2M.gene
CTCF	ctcf	ENSOKIG00005031329	G2M.gene
NEK2	nek2	ENSOKIG00005012940	G2M.gene
NEK2	nek2.1	ENSOKIG00005025708	G2M.gene
G2E3	g2e3	ENSOKIG00005017163	G2M.gene
G2E3	g2e3.1	ENSOKIG00005013265	G2M.gene
GAS2L3	gas2l3	ENSOKIG00005003841	G2M.gene
CBX5	cbx5.1	ENSOKIG00005012086	G2M.gene
CBX5	cbx5	ENSOKIG00005031647	G2M.gene
